# Anlotinib plus chemotherapy as a first-line treatment for gastrointestinal cancer patients with unresectable liver metastases: a multicohort, multicenter, exploratory trial

**DOI:** 10.1038/s41392-024-02051-4

**Published:** 2024-12-09

**Authors:** Jun-Wei Wu, Chen-Fei Zhou, Zheng-Xiang Han, Huan Zhang, Jun Yan, Jun Chen, Chun-Bin Wang, Zhi-Quan Qin, Yong Mao, Xin-Yu Tang, Liang-Jun Zhu, Xiao-Wei Wei, Dong-Hai Cui, Xiu-Li Yang, Min Shi, Li-Qin Zhao, Jin-Ling Jiang, Wei-You Zhu, Hong-Mei Wang, Chun Wang, Ling-Jun Zhu, Jun Zhang

**Affiliations:** 1 Department of Oncology, Ruijin Hospital, Shanghai Jiao Tong University School of Medicine, Shanghai, China; 2 Department of Oncology, The Affiliated Hospital of Xuzhou Medical University, Xuzhou, China; 3 Department of Radiology, Ruijin Hospital, Shanghai Jiao Tong University School of Medicine, Shanghai, China; 4 Department of Oncology, Jiading District Central Hospital Affiliated Shanghai University of Medicine & Health Sciences, Shanghai, China; 5 Department of Chemoradiotherapy, The Affiliated People’s Hospital of Ningbo University, Ningbo, China; 6 Department of Oncology, The Third People’s Hospital of Yancheng, Yancheng, China; 7 Department of Medical Oncology, Zhejiang Provincial People’s Hospital, Affiliated People’s Hospital, Hangzhou, China; 8 Department of Oncology, Affiliated Hospital of Jiangnan University, Wuxi, China; 9 Department of Oncology, Wuxi Branch of Ruijin Hospital, Shanghai Jiao Tong University School of Medicine, Wuxi, China; 10 Department of Medical Oncology, Jiangsu Cancer Hospital, Nanjing, China; 11 Department of Oncology, Nanjing First Hospital, Nanjing Medical University, Nanjing, China; 12 Department of Internal Medicine, Anyang Tumor Hospital, Anyang, China; 13 Department of Oncology, First Affiliated Hospital of Nanyang Medical College, Nanyang, China; 14 Department of Oncology, The First Affiliated Hospital of Nanjing Medical University, Nanjing, China

**Keywords:** Cancer therapy, Gastrointestinal cancer

## Abstract

This multicohort phase II trial (ALTER-G-001; NCT05262335) aimed to assess the efficacy of first-line anlotinib plus chemotherapy for gastrointestinal (GI) cancer patients with unresectable liver metastases. Eligible patients with colorectal cancer (Cohort A) or noncolorectal and nonesophageal GI cancer (Cohort C) received six cycles of anlotinib plus standard chemotherapeutic regimens followed by anlotinib plus metronomic capecitabine as a maintenance therapy. Liver metastasectomy can be performed when liver metastases are converted to resectable lesions. The primary outcome was the investigator-confirmed objective response rate (ORR) in the intention-to-treat population. Among the 47 patients in Cohort A, the ORR was 40.4% (95% CI 26.4–55.7), including 1 with a complete response (CR) and 18 who achieved a partial response (PR). The median progression-free survival (PFS) was 8.7 months (95% CI 7.3-NE), and the median overall survival (OS) was not reached. In Cohort C, 14 of 44 patients achieved a PR, with an ORR of 31.8% (95% CI 18.6–47.6). The PFS and OS were 5.8 months (95% CI 4.8–6.5) and 11.4 months (95% CI 5.8–19.3), respectively. The liver metastasectomy rate in patients with liver-limited disease was 22.7% (5/22) in Cohort A and 6.7% (2/30) in Cohort C. For pancreatic cancer patients, the ORR of the efficacy-evaluable population was 36.0% (9/25), and those with liver-limited metastasis had better survival. Moreover, no new safety concerns emerged. In conclusion, an anlotinib-based first-line regimen demonstrated promising antitumor activity among GI cancer patients with unresectable liver metastases and led to liver metastasectomy in selected patients.

## Introduction

Gastrointestinal (GI) cancers account for approximately 1/4 of the global cancer incidence and more than 1/3 of cancer mortalities in 2022,^1^ with the highest burden in East Asia.^2^ Most GI cancers, including gastric cancer, colorectal cancer (CRC), and pancreatic cancer, are commonly diagnosed at advanced stages with distant metastases. Liver metastasis, occurring in 5%–50% of all GI cancers,^3^ leads to a relatively dismal prognosis, with a 5-year survival rate of less than 10%.^4–6^ Therefore, there is an urgent need to enhance the overall therapeutic effects for GI cancer patients with liver metastases.

Combination therapy is the major strategy for patients with advanced GI cancer and liver metastasis. For patients with liver-limited metastatic CRC (mCRC), conversion metastasectomy of liver lesions after effective treatment can be a viable strategy that leads to a definite cure.^7^ Although a standard of care (SOC) for advanced GI cancer has been established, the need for effective new therapies to improve the overall prognosis is still urgent.^8^ Additionally, anti-angiogenic combination therapies provide benefits to advanced GI cancer patients, mainly in mCRC; however, the modest improvement in objective response rate (ORR) highlights the necessity of optimizing current anti-angiogenic strategies to address the unmet clinical needs.^3,8^

The unique microenvironment of the liver is closely correlated with the antitumor efficacy of systemic treatment leading to treatment failure. Immune tolerance in the liver fosters an immunosuppressive tumor microenvironment (TME) with increased infiltration of regulatory T cells and decreased content of effector CD8^+^ T cells, which leads to immune evasion of tumor cells.^4,9^ The relatively high interstitial fluid pressure in liver metastases caused by tumor-induced angiogenesis can also impede the delivery of drugs and diminish their antitumor activities.^10^ Therefore, novel drug combinations and treatment paradigms that target these critical malignant phenotypes of liver metastasis are worthy of assessment in GI cancers.

Anlotinib is an oral multityrosine kinase inhibitor (MKI) inhibiting vascular endothelial growth factor receptor, fibroblast growth factor receptor, platelet-derived growth factor receptor, and c-kit.^11^ The antitumor efficacy of first-line anlotinib plus chemotherapy has been demonstrated in multiple tumor types, including mCRC and advanced esophageal squamous cell carcinoma (ESCC).^12–14^ In preclinical studies, anlotinib has shown potent effects on promoting vessel normalization and reprogramming the immunosuppressive TME.^15^ An increase in the delivery of cytotoxic drugs was observed after administering anlotinib in vivo, as indicated by a decrease in the interstitial fluid pressure in tumors.^16^ These results suggest that anlotinib is a potential treatment option for controlling liver metastasis. Currently, first-line anti-angiogenic therapy for GI cancer primarily focuses on single-target monoclonal antibodies, with no clinical evidence of MKI plus chemotherapy for GI cancer patients with liver metastasis. The clinical benefits of anlotinib-based combination therapy in this population are worth exploring based on our pervious findings.

We conducted a phase II, multicohort, exploratory trial (ALTER-G-001) to assess the efficacy and safety of first-line anlotinib plus chemotherapy for advanced GI cancer with unresectable liver metastasis. Metronomic capecitabine plus anlotinib was administered as a maintenance therapy to improve the safety profiles of patients during treatment.

## Results

### Clinical characteristics of patients

**Fig. 1:**
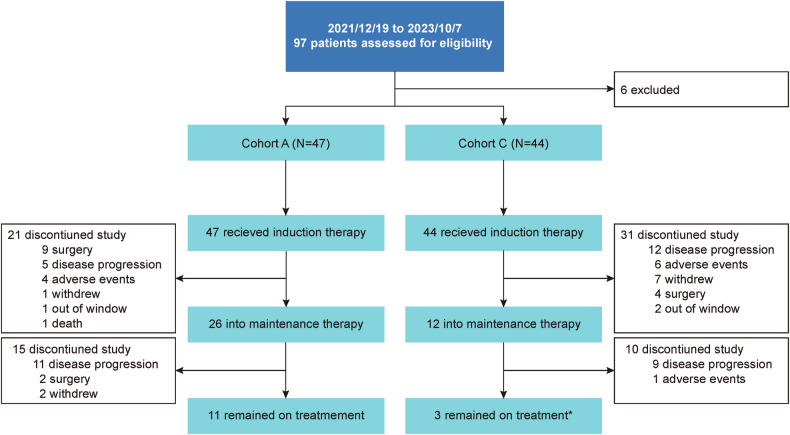
The study flowchart. *Among the three patients in Cohort C, one is receiving induction therapy and two are receiving maintenance therapy

From December 19, 2021 to October 7, 2023, 97 patients were screened for eligibility (Fig. 1). The detailed baseline characteristics of the patients are described in Table 1. Forty-seven CRC patients with a median age of 65 years (range 35–75) constituted the intention-to-treat (ITT) population in Cohort A, and of these, 33 (70.2%) were male. Twenty-two patients (46.8%) were diagnosed with liver-limited disease (LLD) at baseline. Forty-four patients were eligible for enrollment in Cohort C, including 32 patients with pancreatic cancer, 6 patients with gastric or gastroesophageal junction cancer, 5 patients with biliary tract cancer, and 1 patient with duodenal cancer, with a median age of 63.5 years (range 34–74); of these patients, 28 (63.6%) were male. LLD was observed in 30 patients (68.2%).

**Table 1:** Demographic and baseline characteristics of the intention-treat-population (ITT) population in Cohorts A and C

Characteristics	Cohort A (*N* = 47)	Cohort C (*N* = 44)
Age, years, median (range)	65 (35–75)	63.5 (34–74)
Male, *n* (%)	33 (70.2)	28 (63.6)
ECOG performance status, *n* (%)^a^		
0	5 (10.6)	3 (6.8)
1	42 (89.4)	41 (93.2)
Tumor types, *n* (%)		
Colorectal cancer	47 (100.0)	-
Pancreatic cancer	-	32 (72.7)
Gastric or gastro-esophageal junction cancer	-	6 (13.6)
Biliary tract cancer	-	5 (11.4)
Duodenal cancer	-	1 (2.3)
Primary tumor location, *n* (%)		
Colorectal cancer		
Left colon	17 (36.2)	-
Right colon	15 (31.9)	-
Rectum	15 (31.9)	-
Pancreatic cancer		
Head^b^	-	13 (40.6)
Body^b^	-	13 (40.6)
Tail^a^	-	6 (18.8)
No. of metastatic sites, *n* (%)		
1	22 (46.8)	30 (68.2)
2	18 (38.3)	11 (25.0)
≥3	7 (14.9)	3 (6.8)
Sites of metastases, *n* (%)		
Liver	47 (100.0)	44 (100.0)
Lungs	7 (14.9)	3 (6.8)
Peritoneum or retroperitoneum	1 (2.1)	1 (2.3)
Bones	3 (6.4)	1 (2.3)
Lymph nodes	17 (36.2)	12 (27.3)
Liver metastasis, *n* (%)		
Liver metastasis only	22 (46.8)	30 (68.2)
Liver metastasis and metastasis to other sites	25 (53.2)	14 (31.8)
Sum of the diameter of target lesions at baseline, mm, median (range)^c^	72.9 (10.0–191.5)	80.5 (11.6–190.9)
Median time from diagnosis to first dose (range), weeks	3.3 (0.3–168.4)	1.9 (0.3–175.1)
CA19-9 level at baseline, U/mL, median (IQR)	-	1000.0 (298.1–7449.0)
<37^b^	-	2 (6.3)
≥37^b^	-	30 (93.8)
Previous treatments, *n* (%)		
Prior surgery	26 (55.3)	19 (43.2)
Prior chemotherapy	8 (17.0)	3 (6.8)
Prior radiotherapy	0 (0.0)	1 (2.3)

Data are expressed as numbers (%) unless otherwise indicated. The percentage may be greater than 100% because of rounding

*ECOG* Eastern Cooperative Oncology Group, *γ-GT* gamma-glutamyl transferase

^a^ECOG performance status scores range from 0 to 5, with 0 indicating no symptoms and higher scores indicating greater disability

^b^The percentage is based on 32 patients with pancreatic cancer

^c^Tumor size is the sum of the largest diameters of all target lesions (shortest diameter for the lymph nodes)

### Efficacy

**Fig. 2:**
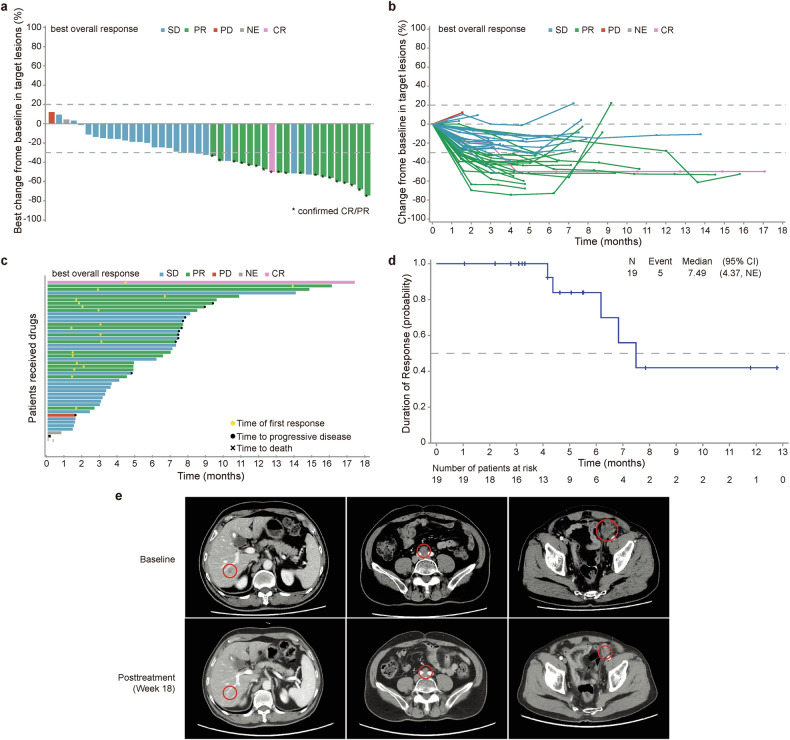
Treatment responses of patients in Cohort A. **a** Waterfall plots of the best percentage changes for the sum of target lesion diameters are shown for individual patients, as assessed by investigators via RECIST version 1.1. Each bar represents one patient in the intention-to-treat (ITT) population. **b** The spider plot displays individual changes in the sum of unidimensional tumor measurements over time relative to the baseline tumor burden. **c** Swimmer plots of the time to tumor response (months) of individual patients as assessed by investigators. Each swim lane represents one patient in the ITT population. **d** Kaplan‒Meier curve of the duration of response. **e** Representative images of a 72-year-old male patient with mCRC who achieved a PR following treatment with the study drug. The CT images of right lobe of the liver, retroperitoneal lymph nodes, and pelvic target lesions at baseline and cycle 6 are shown on the left, middle, and right, respectively. The dotted line in **a** and **b** indicates a 30% reduction and a 20% increase in the target lesion size, respectively. CR complete response, PD progressive disease, PR partial response, SD stable disease, RECIST Response Evaluation Criteria in Solid Tumors, CT computed tomography

In Cohort A, the median duration of treatment (DOT) was 5.5 months (interquartile range [IQR] 3.4–9.0). The investigator-confirmed ORR was 40.4% (19/47, 95% CI 26.4–55.7) and included 1 patient with a complete response (CR) and 18 who achieved a partial response (PR) in the ITT population. Tumor shrinkage after treatment was observed in most patients, and the disease control rate (DCR) was 89.4% (95% CI 76.9–96.5; Fig. 2a). For the efficacy-evaluable population, the investigator-confirmed ORR was 44.2% (19/43, Table 2). The median time to response (TTR) was 1.9 months (range 1.3–13.6), and the duration of response (DoR) was 7.5 months (95% CI 4.4–not estimable [NE]) in the ITT population (Fig. 2b-d). The subgroup analysis did not reveal a significant difference in ORR benefit across diverse clinical subgroups in Cohort A. The ORR of mCRC patients with and without extrahepatic metastases was similar (40.0% vs. 40.9%, *P* = 0.950, supplementary Table 1). A 72-year-old male patient with recurrent mCRC 21 months after primary tumor resection showed metastases involving the liver, retroperitoneal lymph nodes, and pelvis. Following 4 cycles of study drug treatment, he achieved PR. At the data cutoff, 9 cycles of treatment were completed and the PR was maintained for 5 cycles. Representative images of right lobe of the liver, retroperitoneal lymph nodes, and pelvic target lesions at baseline and cycle 6 are shown in Fig. 2e.

**Table 2:** Investigator-confirmed best overall response in the study patients in Cohorts A and C

	Cohort A		Cohort C		Pancreatic cancer	
	ITT (*N* = 47^e^)	Efficacy-evaluable population (*N* = 43)	ITT (*N* = 44^f^)	Efficacy-evaluable population (*N* = 37)	ITT (*N* = 32)	Efficacy-evaluable population (*N* = 25)
Objective response^a^	40.4 (26.4, 55.7)	44.2 (29.1, 60.1)	31.8 (18.6, 47.6)	37.8 (22.5, 55.2)	28.1 (13.8, 46.8)	36.0 (18.0, 57.5)
Complete response	1 (2.1)	1 (2.3)	0 (0.0)	0 (0.0)	0 (0.0)	0 (0.0)
Partial response	18 (38.3)	18 (41.9)	14 (31.8)	14 (37.8)	9 (28.1)	9 (36.0)
Stable disease	23 (48.9)	23 (53.5)	18 (40.9)	18 (48.7)	13 (40.6)	13 (52.0)
Progressive disease	1 (2.1)	1 (2.3)	5 (11.4)	5 (13.5)	3 (9.4)	3 (12.0)
Not evaluable	4 (8.5)	0 (0.0)	7 (15.9)	0 (0.0)	7 (21.9)	0 (0.0)
Disease control	89.4 (76.9, 96.5)	97.7 (87.7, 99.9)	72.7 (57.2, 85.0)	86.5 (71.2, 95.5)	68.8 (50.0, 83.9)	88.0 (68.8, 97.5)
Median time to response (range), months^b^	1.9 (1.3–13.6)		1.4 (1.2–4.6)		1.6 (1.2–3.5)	
Duration of response, months^c^	7.5 (4.4, NE)		6.8 (3.8, 9.2)		4.6 (2.9, 8.0)	
Depth of response^d^						
30%-50%	8 (42.1)		6 (42.9)		3 (33.3)	
50%-70%	10 (52.6)		5 (35.7)		5 (55.6)	
70%-100%	1 (5.3)		3 (21.4)		1 (11.1)	

Data are expressed as n (%; 95% CI) or n (%) unless otherwise specified

*ITT* intention-to-treat, *CR* complete response, *NE* not evaluable, *NR* not reached, *PR* partial response, *SD* stable disease

^a^Assessed by investigators via RECIST version 1.1. CR and PR are confirmed 4 weeks apart, and SD should persist for at least 8 weeks. The objective response measures include CR and PR; the disease control measures include CR, PR, and SD

^b^Time to response is defined as the time from the first dose of study medication to the first recorded response (CR or PR) and is calculated only for patients who achieve the best overall response of CR or PR

^c^Duration of response is defined as the time from the first recorded response (CR or PR) to the first recorded disease progression (based on RECIST 1.1) or death from any cause, whichever occurs first

^d^Depth of response is defined as the maximal tumor shrinkage observed

^e^In Cohort A, 4 patients were NE due to adverse events or patient withdrawal

^f^In Cohort C, 7 patients were NE due to withdrawal of consent, patient withdrawal, adverse events, treatment beyond the time window, or treatment interruption for more than 1 cycle

**Fig. 3:**
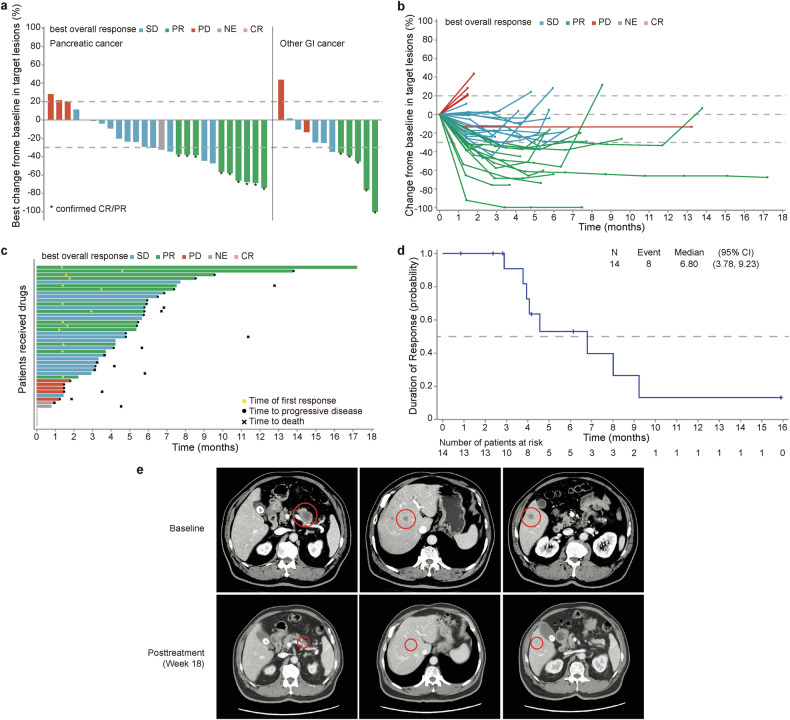
Treatment responses of patients in Cohort C. **a** Waterfall plots of the best percentage changes for the sum of target lesion diameters are shown for individual patients assessed by investigators via RECIST version 1.1. Each bar represents one patient in the intention-to-treat (ITT) population. **b** The spider plot displays individual changes in the sum of unidimensional tumor measurements over time relative to the baseline tumor burden. **c** Swimmer plots of the time to tumor response (months) of individual patients as assessed by investigators. Each swim lane represents one patient in the ITT population. **d** Kaplan‒Meier curve of the duration of response. **e** Representative images of a 62-year-old male patient with pancreatic cancer and liver metastases who achieved a PR following treatment with the study drug. The CT images of one target lesion in the pancreatic body and two target lesions in the right lobe of the liver at baseline and cycle 6 are shown on the left, middle, and right, respectively. The dotted line in **a** and **b** indicates a 30% reduction and a 20% increase in the target lesion size, respectively. CR, complete response; PD, progressive disease; PR, partial response; SD, stable disease; RECIST, Response Evaluation Criteria in Solid Tumors; CT, computed tomography

In Cohort C, the median DOT was 3.7 months (IQR 1.4–5.8). The investigator-confirmed ORR was 31.8% (14/44, 95% CI 18.6–47.6) in the ITT population. Among the 14 patients who achieved a PR, 8 experienced a greater than 50% reduction in tumor size (Fig. 3a). The DCR was 72.7% (95% CI 57.2–85.0). For the efficacy-evaluable population, the investigator-confirmed ORR was 37.8% (14/37, Table 2). The median TTR was 1.4 months (range 1.2–4.6), and the median DoR reached 6.8 months (95% CI 3.8–9.2) in the ITT population (Fig. 3b–d). A 62-year-old male pancreatic cancer patient with liver metastases achieved a PR following two cycles of study drug treatment. The PR sustained for 22 cycles. Non-target liver lesions also disappeared by the end of cycle 9. Representative images of one target lesion in the pancreatic body and two target lesions in the right lobe of the liver at baseline and cycle 6 are shown in Fig. 3e.

Eleven patients (11/47, 23.4%) in Cohort A and 4 patients (4/44, 9.1%) in Cohort C (2 with gastric cancer, 1 with gallbladder cancer, and 1 with pancreatic cancer) underwent surgical resection after treatment. Among the LLD patients, 5 patients (5/22, 22.7%) in Cohort A and 2 patients (2/30, 6.7%) in Cohort C underwent segmentectomy of the liver, while 1 patient in Cohort A underwent radiofrequency ablation.

### Survival

**Fig. 4:**
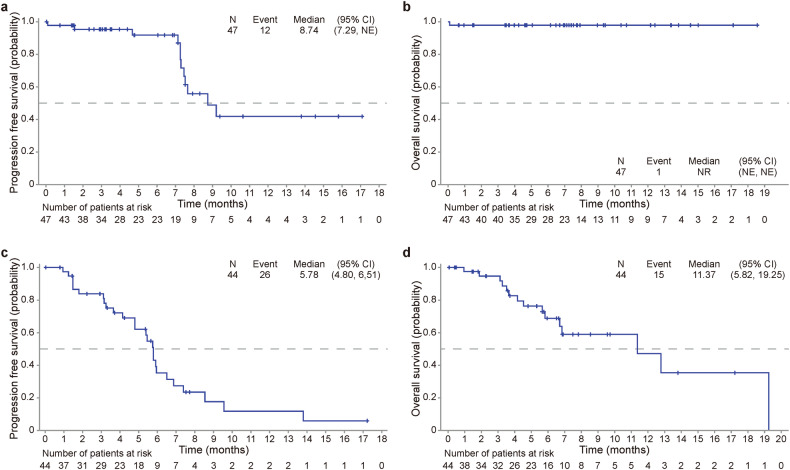
Survival curves of patients in Cohorts A and C. **a** Kaplan‒Meier curve of progression-free survival (PFS) of patients in Cohort A. **b** Kaplan‒Meier curve of overall survival (OS) of patients in Cohort A. **c** Kaplan‒Meier curve of PFS of patients in Cohort C. **d** Kaplan‒Meier curve of the OS of patients in Cohort C

As of February 29, 2024, the median follow-up duration of all patients was 6.9 months (95% CI 5.7-7.5). In Cohort A, 12 progression-free survival (PFS) events occurred, and the median PFS was 8.7 months (95% CI 7.3-NE) (Fig. 4a). The 6- and 12-month PFS rates were 91.8% (95% CI 76.1–97.4) and 41.9% (95% CI 19.2–63.2), respectively (supplementary Table 2). The overall survival (OS) data were not mature because only 1 death event occurred (Fig. 4b). No significant difference in PFS across clinical subgroups was observed (supplementary Table 1).

In Cohort C, 26 PFS events were observed, and the median PFS was 5.8 months (95% CI 4.8–6.5) (Fig. 4c). The 6- and 12-month PFS rates were 35.3% (95% CI 18.5–52.6) and 11.8% (95% CI 2.4–29.4), respectively (supplementary Table 2). The deaths of 15 patients were recorded. The median OS was 11.4 months (95% CI 5.8–19.3) (Fig. 4d), and the 12-month OS rate was 47.2% (95% CI 21.3–69.5).

### Treatment efficacy in pancreatic cancer

**Fig. 5:**
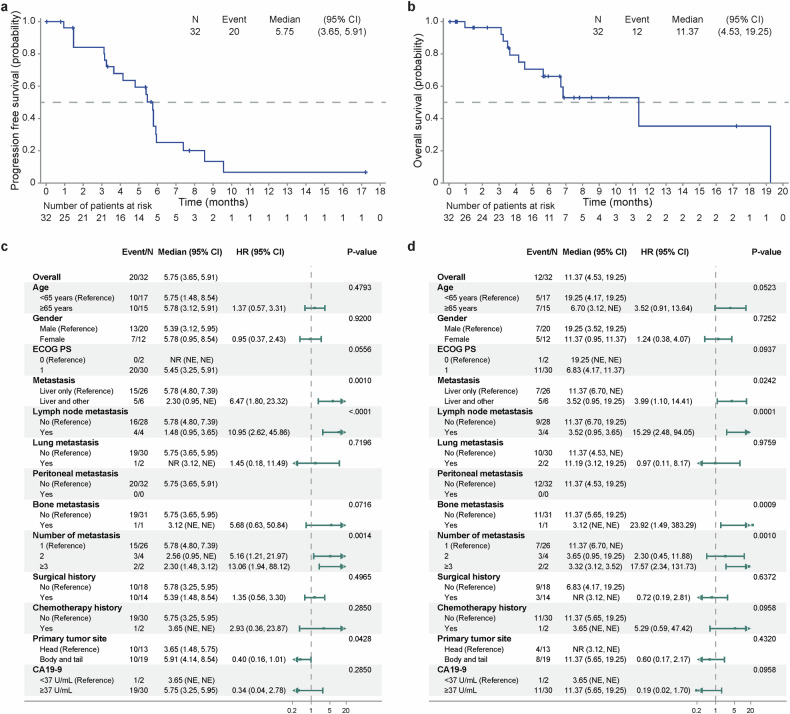
Survival curves of pancreatic cancer patients in Cohort C and subgroup analysis. **a** Kaplan‒Meier curve of the progression-free survival (PFS) of patients with pancreatic cancer. **b** Kaplan‒Meier curve of the overall survival (OS) of patients with pancreatic cancer. **c** Subgroup analysis of PFS. **d** Subgroup analysis of OS

Thirty-two pancreatic cancer patients were enrolled in Cohort C, 90.6% of whom (29/32) received anlotinib plus gemcitabine/nab-paclitaxel chemotherapy, and 10 patients received maintenance therapy. In the efficacy-evaluable population, the investigator-confirmed ORR was 36.0% (9/25, Table 2). The median PFS and OS were 5.8 months (95% CI 3.7–5.9) and 11.4 months (95% CI 4.5–19.3), respectively (Fig. 5b). The 12-month OS rate was 35.3% (95% CI 8.3-64.6; supplementary Table 2). The subgroup analysis revealed that the ORRs of patients with and without extrahepatic metastases were 16.7% and 30.8%, respectively (supplementary Table 3). Patients with LLD had significantly better PFS (5.8 months, vs. 2.3 months, *P* = 0.001; Fig. 5c) and OS (11.4 months, vs. 3.5 months, *P* = 0.024; Fig. 5d) than patients with multiorgan metastasis. Lymph node metastasis and metastasis at ≥3 sites were also associated with poor survival.

### Safety

The median duration of anlotinib exposure was 5.5 months (IQR 3.4-9.0) in Cohort A and 3.7 months (IQR 1.4–5.8) in Cohort C. Two patients (4.3%) in Cohort A and 4 (9.1%) in Cohort C discontinued at least one treatment component due to adverse events. Dose reductions of any study drugs occurred in 10 patients (21.3%) in Cohort A and 9 (20.5%) in Cohort C (Supplementary Table 4). All treated patients were included in the safety population (*N* = 91). The rate of treatment-related AEs (TRAEs) of any grade was 86.8% (79/91) during induction therapy and 55.3% (21/38) during maintenance therapy. The rate of grade 3 or worse TRAEs was 45.1% (41/91) during induction therapy and 10.5% (4/38) during maintenance therapy (Table 3). Neutropenia was the most common grade 3 or worse TRAE (12.8%), followed by thrombocytopenia (10.6%) and hypertension (6.4%) in Cohort A, while the most common was neutropenia (18.2%) followed by leucopenia (13.6%) and thrombocytopenia (9.1%) in Cohort C (Table 3). Common TRAEs in Cohort A and Cohort C were similar (supplementary Table 5). The rate of serious AEs was 25.3% (23/91), and those that led to hospitalization are listed in supplementary Table 6. No treatment-related deaths were reported in either Cohort A or C.

**Table 3:** Treatment-related adverse events occurring in ≥ 10% of the safety population in Cohorts A (*N* = 47) and C (*N* = 44)

Cohort A		Cohort C			
TRAEs	Any grade	Grade 3 or higher	TRAEs	Any grade	Grade 3 or higher
Any events	45 (95.7)	19 (40.4)	Any events	37 (84.1)	23 (52.3)
Induction therapy	42 (89.4)	19 (40.4)	Induction therapy	37 (84.1)	22 (50.0)
Maintenance therapy*	11 (42.3)	2 (7.7)	Maintenance therapy	10 (83.3)	2 (16.7)
TRAEs			TRAEs		
Neutropenia	20 (42.6)	6 (12.8)	Neutropenia	20 (45.5)	8 (18.2)
Leucopenia	18 (38.3)	0 (0.0)	Leucopenia	20 (45.5)	6 (13.6)
Thrombocytopenia	17 (36.2)	5 (10.6)	Anemia	15 (34.1)	1 (2.3)
Palmar-plantar erythrodysesthesia syndrome	11 (23.4)	1 (2.1)	Lymphocytopenia	10 (22.7)	2 (4.5)
Hypertension	10 (21.3)	3 (6.4)	Thrombocytopenia	9 (20.5)	4 (9.1)
Nausea	9 (19.2)	0 (0.0)	Hypertension	7 (15.9)	2 (4.5)
Fatigue	8 (17.0)	0 (0.0)	Reduced body weight	6 (13.6)	0 (0.0)
Diarrhea	8 (17.0)	0 (0.0)	γ-GT increased	5 (11.4)	3 (6.8)
Anemia	8 (17.0)	0 (0.0)	Abnormal hepatic function	5 (11.4)	0 (0.0)
Hyperlipidemia	7 (14.9)	0 (0.0)	Rash	5 (11.4)	0 (0.0)
Aspartate aminotransferase increased	7 (14.9)	0 (0.0)	-	-	-
Blood bilirubin increased	7 (14.9)	0 (0.0)	-	-	-
Vomiting	6 (12.8)	0 (0.0)	-	-	-
Abnormal hepatic function	5 (10.6)	2 (4.3)	-	-	-
Thyroid stimulating hormone elevated	5 (10.6)	0 (0.0)	-	-	-
During maintenance therapy			-	-	-
Thrombocytopenia	5 (10.6)	1 (2.1)	-	-	-

Listed in descending order of frequency are all treatment-related adverse events (TRAEs) that occurred in ≥10% of the patients or any grade 3 or higher TRAEs that occurred during the trial period or within the 30 days thereafter (within 90 days for serious events), regardless of attribution to any trial regimen by an investigator

*Twenty-six patients received maintenance therapy in Cohort A, twelve patients received maintenance therapy in Cohort C

For patients who underwent liver metastasectomy, the median time interval from the last dose of anlotinib to surgery was 3.3 weeks (range 1.3–23.3). The rate of perioperative treatment-emergent adverse events (TEAEs) of any grade was 46.7% (supplementary Table 7). No bleeding events were reported by those patients.

## Discussion

This multicohort exploratory study revealed promising antitumor effects of first-line anlotinib plus chemotherapy for patients with advanced GI cancer and liver metastasis, primarily those with mCRC and advanced pancreatic cancer. After induction therapy, selected patients with LLD were eligible for liver metastasectomy. Metronomic capecitabine combined with anlotinib as a maintenance therapy showed excellent safety profiles and did not impair the survival of patients. The results of this exploratory study support further investigations of anlotinib, a multitarget tyrosine kinase inhibitor, as a component of systemic therapy for GI cancer patients.

Currently, this novel combination regimen and treatment paradigm (anlotinib plus chemotherapy for induction, plus metronomic capecitabine as a maintenance therapy) has not been fully assessed in advanced GI cancer, especially in patients with unresectable liver metastasis. In the present study, the investigator-confirmed ORRs of efficacy-evaluable mCRC and pancreatic cancer, the two major cancers in the enrolled patients, were 44.2% and 36.0%, respectively. The median PFS was 8.7 months among mCRC patients and 5.8 months among pancreatic cancer patients. These efficacies are comparable with those found in historical data. In the NO16966 trial, the ORR of bevacizumab in combination with oxaliplatin-based chemotherapy was 38% according to the independent response review committee assessment, while the PFS was 9.4 months.^17^ Gemcitabine plus nab-paclitaxel was the primary regimen used to treat pancreatic cancer patients in this study. In the MPACT trial, the response rate was 23%, and the PFS was 5.5 months for pancreatic cancer patients treated with gemcitabine plus nab-paclitaxel.^18^ The OS of the pancreatic cancer patients in this study was 11.4 months, which was comparable to that of patients treated with the FOLFIRINOX regimen.^19^ Although the ORR of the ITT population did not meet our expectations, over 80% of patients achieved tumor shrinkage, which still indicated the potent tumor control activity of anlotinib in advanced GI cancer. Currently, no angiogenesis inhibitor has been demonstrated to be efficacious for the treatment of metastatic pancreatic cancer. The promising efficacy of anlotinib plus chemotherapy in pancreatic cancer patients with liver-limited metastasis provides valuable information for further randomized controlled trials to validate the antitumor efficacy of anlotinib in these patients.

Conversion surgery for liver metastasis after effective systemic treatment provides an opportunity to cure unresectable liver-limited mCRC. Currently, attempts to apply conversion surgery in other GI cancers, such as pancreatic cancer and gastric cancer, are being investigated.^20,21^ Although a consensus has not been established, survival benefits have been reported in highly selected GI cancer patients who underwent either primary lesion resection alone or resection combined with liver metastasectomy after a favorable response to systemic treatment.^22^ Anlotinib, an antiangiogenic MKI, can not only inhibit tumor angiogenesis but also participates in modulating the immunosuppressive microenvironment of the liver, which might lead to a potent inhibitory effect on liver metastasis and improve its resectability. In this study, the liver metastasectomy rate of LLD patients in Cohort A was 22.7%. Four patients in Cohort C, including those with gastric cancer, gallbladder cancer, and pancreatic cancer, also underwent surgical resection after treatment. The R0 metastasectomy rate in mCRC patients treated with bevacizumab-based combination therapy was 6.3% in the NO16966 trial.^23^ Due to the limited sample size, survival differences between patients who underwent surgery and those who did not could not be analyzed in this study. Moreover, the lack of a universal definition of surgical resectability of liver metastases in GI cancer patients complicates the comparison of surgical conversion rates across different trial populations. A randomized controlled trial with a larger population size should be performed to validate the effect of anlotinib on improving the conversion resection rate and its survival benefit in mCRC patients as well as in patients with other GI cancers.

The safety profiles of chemotherapy plus anlotinib were similar between the two cohorts and were comparable with those of the individual components reported in other trials.^24,25^ No unanticipated serious TEAEs or severe surgical complications were observed. The most common grade 3 or worse TRAEs in this study were hematological toxicities, including neutropenia, leucopenia, and thrombocytopenia, which were attributed mainly to chemotherapy. The addition of anlotinib did not increase the risk of hematological toxicity during chemotherapy. The rate of grade 3 or worse hypertension caused by angiogenesis inhibitors was also low (6.4% for Cohort A and 4.5% for Cohort C).^26^ Palmar-plantar erythrodysesthesia (PPE) is a common AE associated with both anlotinib and capecitabine, which might cause overlapping toxicity. Although the incidence rate of PPE in Cohort A was higher than that in Cohort C, its overall incidence in the present study was lower than that reported in previous studies.^13^ The application of metronomic capecitabine during maintenance therapy might also contribute to the low incidence of PPE, which minimizes overlapping toxicity. However, some studies have shown that in mCRC patients, capecitabine-related PPE responses are associated with improved OS.^27^

Excellent safety profiles should be sought during maintenance therapy, with a rate of grade 3 or worse TRAEs no higher than 10.5%. Metronomic chemotherapy is a cancer treatment paradigm in which cytotoxic drugs with minimum effective doses and high frequencies are used.^28^ In addition to its excellent safety profile, the antitumor effects of metronomic chemotherapy have been observed in multiple types of cancer, and thus this therapy is a promising option for maintenance therapy. Our preclinical studies revealed that metronomic capecitabine exerts potent antiangiogenic effects and modulates the TME, which indicates that it has a synergistic effect with anlotinib.^29,30^ Long-term disease control was observed in patients who received maintenance therapy, even those with pancreatic cancer, and the survival of patients in both Cohort A and Cohort C was comparable with that in previously reported studies; this indicates the efficacy of anlotinib plus metronomic capecitabine as a maintenance therapy. Therefore, due to its good tolerance and promising efficacy, anlotinib plus metronomic capecitabine can be a novel maintenance regimen for GI cancer patients, but future studies with larger sample sizes are still needed.

This study had some potential limitations, including the lack of a control arm and the limited sample size. The assessment of tumor response by investigators without an independent central review committee might have also introduced bias in the ascertainment of PFS. The proportion of CRC patients with near-resectable liver metastases was low, which might have impacted the liver metastasectomy rate. The relatively high tumor burden might have also caused difficulty in the accurate evaluation of the overall efficacy of this regimen. Nevertheless, this was a phase II study to assess the feasibility of anlotinib-based regimens for the treatment of patients with advanced GI cancer for translational purposes on the basis of our preclinical data. Preliminary efficacy data in pancreatic cancer patients with liver metastasis provide valuable information for future confirmatory studies.

In conclusion, six cycles of induction treatment with anlotinib plus standard chemotherapy followed by anlotinib plus metronomic capecitabine maintenance therapy exhibited promising antitumor activities and excellent tolerance, which provides a new first-line treatment option for patients with advanced GI cancer and unresectable liver metastasis. These findings support further exploration of the role of anlotinib in GI cancers.

## Materials and methods

### Ethics

The study protocols and all amendments were approved by the institutional review board or ethics committee of each participating center. All patients provided written informed consent. The trial was performed per the Declaration of Helsinki, the Good Clinical Practice guidelines of the International Conference on Harmonization, and relevant laws and directives in China. This trial is registered with CLINICALTRIALS. GOV (NCT05262335).

### Study design and participants

This phase II multicenter, multicohort, single-arm, investigator-initiated exploratory study (ALTER-G-001) enrolled adult patients (aged 18 to 75 years) who had histologically or cytologically confirmed GI cancer with unresectable liver metastasis (with or without extrahepatic metastases). Cohort A consisted of patients with stage IV CRC, while Cohort C consisted of patients with GI cancers other than CRC and ESCC (such as gastric cancer, pancreatic cancer, and biliary tract cancer). Included patients also had at least one measurable metastatic liver lesion per the Response Evaluation Criteria in Solid Tumors (RECIST), version 1.1. Other key eligibility criteria were an Eastern Cooperative Oncology Group (ECOG) performance status of 0–1, adequate hematologic and organ function, and a life expectancy of ≥12 weeks. Patients should have received no prior systemic therapy, including chemotherapy, targeted therapy, or immune therapy. Patients with recurrent disease, those who received prior neoadjuvant chemoradiotherapy plus curative surgical resection, and those treated with prior adjuvant chemoradiotherapy or curative concurrent chemoradiotherapy were eligible if treatment was completed more than six months before enrollment. The key exclusion criteria were a history of primary malignant cancer, with the exception of cured nonmelanoma skin cancer or cured lentigo maligna melanoma, and cured carcinoma in situ; active bleeding in the primary and/or metastatic lesions within two months of enrollment; arterial and/or venous thromboembolic events within six months of enrollment; current thrombolytic or anticoagulation treatment; and GI diseases with bleeding diathesis at risk of bleeding, as determined by the investigators, perforation, obstruction, or fistulation. Patients with brain and leptomeningeal metastases were ineligible. Patients who had received radiation therapy or surgery within 30 days of enrollment were also excluded. The full eligibility criteria are provided in the trial protocol.

### Procedures

For induction therapy, patients in Cohort A received 12 mg oral anlotinib (Chia Tai Tianqing Pharmaceutical Co., Ltd.) once daily on days 1 to 14 of each cycle^31^ plus the CAPEOX regimen (850 mg/m^2^ capecitabine given orally twice a day on days 1 to 14 of each cycle and 130 mg/m^2^ oxaliplatin given intravenously on day 1 of each cycle). Patients in Cohort C received 12 mg oral anlotinib once daily from days 1 to 14 of each cycle plus an SOC chemotherapeutic regimen at the discretion of the investigators. Each cycle lasted three weeks. The resectability of the liver metastases was assessed via multidisciplinary consultation after every two cycles during the six cycles of induction therapy. Patients who were eligible for surgery discontinued treatment four weeks prior to surgery or at the discretion of the investigators. Local treatment of liver metastases was also allowed. Patients who were ineligible for surgery and who achieved disease control (CR, PR or stable disease [SD]) per RECIST, version 1.1, received maintenance therapy consisting of 12 mg of anlotinib once daily for two weeks with one week off plus metronomic capecitabine chemotherapy (500 mg, twice daily, per os) (Cohort A), or anlotinib plus chemotherapy, in which the chemotherapy was preferably capecitabine metronomic chemotherapy (500 mg, twice daily, per os) (Cohort C). Maintenance therapy was continued until disease progression, death, or unacceptable toxicity.

Anlotinib was reduced by up to two doses (12 mg/d to 10 mg/d and 10 mg/d to 8 mg/d). The protocol-defined criteria for dose modification of anlotinib, capecitabine or other chemotherapeutic agents are available in the trial protocol.

### Assessments

Responses were evaluated radiologically with contrast computed tomography (CT) or magnetic resonance imaging (MRI) by investigators every two cycles during induction, every three cycles thereafter and at the end of the study per the RECIST (v1.1) criteria. CR and PR were confirmed 4 weeks apart, and SD should persist for at least 8 weeks. Patients were followed-up every six months to assess survival until the death of the patient or until the data cutoff date, whichever came first.

The occurrence, frequency, and severity of AEs were assessed throughout the treatment period and up to 30 days after the final treatment dose was given according to the National Cancer Institute Common Terminology Criteria of Adverse Events (NCI CTCAE) version 5.0. The incidence of perioperative TEAEs was assessed from the end of systemic treatment to 30 days after surgery. Serious AEs were observed in this study.

### Outcomes

The primary end point of the study was the investigator-confirmed ORR, which is defined as the proportion of patients who achieved CR and PR as the best overall response per RECIST, version 1.1. The secondary endpoints included PFS, defined as the duration from treatment initiation to disease progression in patients who did not undergo surgery, or death, whichever occurred first; OS, defined as the duration from the date of treatment initiation to the date of death from any cause; DCR, defined as the proportion of patients who achieved CR, PR and SD as the best overall response; DoR, calculated from the first documented CR or PR to the first documented disease progression or death, whichever occurred first; and the conversion rate of liver metastasis, defined as the proportion of patients with initially unresectable liver metastases who become eligible for surgical resection after undergoing conversion therapy.

### Statistical analysis

Based on an ORR of 47% with first-line CAPEOX plus bevacizumab in NO16966,^32,33^ and assuming a dropout rate of 20%, a sample size of 45 patients in Cohort A was required to achieve an ORR of 70% with first-line CAPEOX plus anlotinib and maintenance therapy. Furthermore, based on an ORR of 19.4–47.8% with first-line SOC chemotherapy for GI cancers,^34–38^ a sample size of 40 patients in Cohort C was anticipated to achieve an ORR of 47% with first-line SOC chemotherapy plus anlotinib and maintenance therapy, which resulted in 20% attrition.

This study followed the ITT principle, and the full analysis set included all patients who had received at least one dose of the study medications. Efficacy analysis was based on the ITT population. The ORR, DCR and conversion rate of liver metastases and their 95% CIs were estimated via the Clopper‒Pearson method. The follow-up duration was estimated using the reverse Kaplan‒Meier method. The median PFS and OS were estimated with the Kaplan‒Meier method, and the corresponding 95% CIs were calculated using the Brookmeyer‒Crowley method via log transformation. Patients who underwent surgery were censored for PFS at the final radiological evaluation before surgery.

The safety set included all patients who had received at least one dose of the study medications and had available safety records. Safety was analyzed mainly by descriptive statistics.

All *P* values were 2-sided, with *P* < 0.05 considered statistically significant. Statistical analysis was performed via SAS version 9.4.

## Supplementary information

Revised Sigtrans_Supplementary_Materials

Study Protocol

## Data Availability

The datasets used and/or analyzed in the current study are available from the corresponding author upon reasonable request.
