# Supplementary material for: Anlotinib plus chemotherapy as a first-line treatment for gastrointestinal cancer patients with unresectable liver metastases: a multicohort, multicenter, exploratory trial
Source: Signal Transduct Target Ther. 2024 Dec 9;9:344. doi: 10.1038/s41392-024-02051-4 (PMC11625826; doi:10.1038/s41392-024-02051-4)
Supplement: Supplementary file 1 — Revised Sigtrans_Supplementary_Materials [file 41392_2024_2051_MOESM1_ESM.docx]

Supplementary Materials for

Anlotinib plus chemotherapy as a first-line treatment for gastrointestinal cancer patients with unresectable liver metastases: a multicohort, multicenter, exploratory trial

Jun-Wei Wu^#^, Chen-Fei Zhou^#^, Zheng-Xiang Han, Huan Zhang, Jun Yan, Jun Chen, Chun-Bin Wang, Zhi-Quan Qin, Yong Mao, Xin-Yu Tang, Liang-Jun Zhu, Xiao-Wei Wei, Dong-Hai Cui, Xiu-Li Yang, Min Shi, Li-Qin Zhao, Jin-Ling Jiang, Wei-You Zhu, Hong-Mei Wang, Chun Wang, Ling-Jun Zhu^*^, Jun Zhang^*^

# These authors contributed equally to the manuscript and thus share the first authorship.

^*^Correspondence to: junzhang10977@sjtu.edu.cn; zhulingjun@njmu.edu.cn.

**This PDF file includes:**

Materials and Methods

Supplementary Text

Tables S1 to S7

**Other Supplementary Materials for this manuscript include the following:**

Study protocol

**Materials and Methods**

Inclusion Criteria

Patients who meet all the following inclusion criteria can be included in this trial:

1) The patient voluntarily joined the study, signed the informed consent form, had good compliance and cooperated with the follow-up;

2) Male or female patients aged 18-75 years;

3) Cohort A: Patients histopathologically or cytologically confirmed colorectal cancer at stage IV (TAnyNAnyM1) with unresectable liver metastases;

Cohort B: Patients histopathologically or cytologically confirmed esophageal squamous cell carcinoma at stage IVb (TAnyNAnyM1) with liver unresectable metastases (excluding mixed adenosquamous carcinoma);

Cohort C: Patients histopathologically or cytologically confirmed other gastrointestinal tumors with liver metastases (excluding gastrointestinal stromal tumors, neuroendocrine tumors and other malignant tumors of non-glandular epithelial origin);

4) No previous systemic therapy, including chemotherapy, targeted therapy and immunotherapy;

Note: Patients who relapsed after receiving neoadjuvant (radio)chemotherapy plus radical surgery for more than 6 months, or relapsed after receiving adjuvant (radio)chemotherapy or radical concurrent chemoradiotherapy for more than 6 months were eligible;

5) At least one measurable lesion in the liver metastases according to the Response Evaluation Criteria in Solid Tumors (RECIST) Version 1.1, and the target lesions must contain liver metastases. The selected target lesions have not received local therapy such as radiotherapy or TACE within 6 months;

Note: Lesions in the area of previous local treatment can also be selected as target lesions if progression is confirmed and meets RECIST 1.1 criteria;

6) Palliative therapy for localized lesions (non-target lesions) should be completed for> 2 weeks;

7) ECOG PS score: 0-1;

8) Life expectancy of more than 3 months;

9) Adequate organ and bone marrow function, defined as meeting the following criteria (within 7 days prior to enrollment):

a) The blood routine examination criteria shall meet:

- Hemoglobin level (HB) ≥ 90 g/L (no blood transfusion within 14 days);
- Absolute neutrophil count (ANC) ≥ 1.5 × 10^9^/L;
- Platelet count (PLT) ≥ 90 × 10^9^/L.

b) Biochemical tests must meet the following criteria:

- Serum total bilirubin (TBIL) ≤ 1.5 × upper limit of normal (ULN);
- Alanine aminotransferase (ALT) and aspartate aminotransferase (AST) ≤ 5 ULN;
- Serum creatinine (Cr) ≤ 1.5 ULN or creatinine clearance (CCr) ≥ 60ml/min; (Cockcroft-Gault formula)

c) Adequate coagulation function defined as international normalized ratio (INR) or prothrombin time (PT) ≤ 1.5 × ULN;

10) Females of childbearing potential are required to use adequate contraception and avoid breastfeeding from screening through 3 months after discontinuation of study treatment. Have a negative pregnancy test prior to initiation of dosing or meet one of the following criteria to demonstrate absence of risk of pregnancy:

a) Postmenopausal is defined as age greater than 50 years and amenorrhea for at least 12 months after stopping all exogenous hormone replacement therapy;

b) Women younger than 50 years of age may also be considered postmenopausal if they have been amenorrheic for 12 months or more after stopping all exogenous hormone therapy and their luteinizing hormone (LH) and follicle stimulating hormone (FSH) levels are within the laboratory's postmenopausal reference range;

c) Undergo irreversible sterilization, including hysterectomy, oophorectomy, or salpingectomy, except tubal ligation.

Males must agree to use an adequate method of contraception or have been surgically sterile during the trial and for 8 weeks after receiving trial drug.

Exclusion Criteria:

Patients with any of the following will not be enrolled in the study:

1) Patients with active bleeding within 2 months from the primary lesion and/or metastases;

2) Hyperactive/venous thrombosis within 6 months, such as cerebrovascular accident (including temporary ischemic attack), deep vein thrombosis and pulmonary embolism;

3) For patients receiving thrombolytic or anticoagulant therapy such as warfarin, heparin or their analogues, low-dose heparin (daily dose of 6,000 ~ 12,000 U for adults) or low-dose aspirin (daily dose ≤ 100 mg) for preventive purposes is allowed provided INR ≤ 1.5 × ULN;

4) Patients with gastrointestinal diseases with bleeding tendency (such as active gastrointestinal ulcer) or investigators judgment that may cause gastrointestinal bleeding, perforation or obstruction, or patients with established fistula;

5) Receiving radiotherapy or surgery within 30 days, except biopsy and palliative treatment for non-target lesions (note: patients undergoing non-major trauma surgery can receive this protocol as early as 15 days after surgery if they recover quickly after surgery and can use anti-angiogenic drugs as assessed by the investigator);

6) Patients with HER2-positive gastric adenocarcinoma;

7) A history of immunodeficiency, including HIV positive or other acquired or congenital immunodeficiency disorders, or a history of organ transplantation;

8) Patients with brain metastasis and/or leptomeningeal metastasis; for subjects with neurological symptoms, CT/MRI should be performed to rule out brain metastasis;

9) Patients with any severe and/or uncontrolled disease including:

- Patients with hypertension that is not well controlled by single antihypertensive drug therapy (systolic blood pressure ≥ 150 mmHg, diastolic blood pressure ≥ 100 mmHg); or use two or more antihypertensive drugs to control blood pressure;
- Patients with acute myocardial infarction, malignant arrhythmia (including QT interval > 450 ms for males and > 470 ms for females) and grade II and above congestive heart failure (New York Heart Association (NYHA) classification);
- Active or uncontrolled serious infection (NCI-CTC AE Grade ≥ 2 infection);
- Liver diseases such as cirrhosis, decompensated liver disease, active hepatitis or chronic hepatitis (HBV-DNA > 1000 IU/mL) requiring antiviral therapy;
- Poor glycemic control in diabetic patients (fasting blood glucose > 10 mmol/L);
- Urinalysis showed urine protein ≥ + +, and confirmed 24-hour urine protein > 1.0 g;

10) Patients with clinically significant ascites, including any ascites that are detected by physical examination, previously treated or currently treated ascites. Asymptomatic patients with only small amount of ascites on imaging were eligible;

11) Patients with moderate pleural effusion, or a large amount of pleural effusion on one side, or caused respiratory dysfunction requiring drainage;

12) Uncontrolled metabolic disorder or other nonmalignant organ or systemic disease or reaction secondary to cancer, leading to a high medical risk and/or uncertainty in survival evaluation;

13) Known active pulmonary tuberculosis;

14) Interstitial lung disease requiring steroid hormone therapy;

15) Patients with significant malnutrition;

16) Known hypersensitivity to the study drug;

17) Patients with a history of psychotropic drug abuse and unable to quit or with mental disorders;

18) Female patients who are pregnant or lactating;

19) Participation in other clinical trials within four weeks;

20) History of other primary malignancies, except for the following: 1) malignancies that were in complete remission for at least 2 years prior to enrollment and did not require other treatment during the study period; 2) non-melanoma skin cancer or lentigo maligna with no sign of recurrence after adequate treatment; 3) carcinoma in situ with no sign of recurrence after adequate treatment;

21) Patients with concomitant diseases that seriously jeopardize the patient's safety or prevent patients from completing the study according to the investigator's judgment.

Withdrawal Criteria

1) Use of other anti-tumor therapies (including chemotherapy, targeted therapy or biological agents, etc.) that may affect the evaluation of efficacy during the study;

2) Patients who experienced serious adverse events and longer suitable for continued participation in the study according to the investigators’ judgment, or have unintended pregnancy;

3) Patients who are unwilling to continue the clinical trial and insist on withdrawing;

4) The investigator considered termination of the study necessary

Removal criteria

1) Wrong dose and method of administration;

2) Patients who have received chemotherapy or drugs beyond the protocol during the trial;

3) Those who do not meet the criteria but were included by mistake;

4) Patients without medication;

Note: Patients who met criteria 1-2 were included in the safety analysis.

Discontinuation Criteria

1) Patients with PD;

2) The researchers found severe safety problems based on the decisions of investigators;

3) Drug withdrawal for more than one cycle;

4) Dose reduction for more than 2 times;

5) Surgery is feasible after treatment and patients voluntarily undergo surgery.

Note: In the first 6 cycles of treatment, if one of the chemotherapy drugs has been dose reduced more than twice or discontinued for more than one cycle, while the other chemotherapy drugs can be used alone (e.g., only platinum is intolerant, while capecitabine, paclitaxel or gemcitabine are still tolerable), the latter and anlotinib can be retained; if the retained chemotherapy drugs cannot be used alone, e.g., platinum, the patients should be withdrawn from the study. After 6 cycles, anlotinib maintenance could be continued if capecitabine was intolerable while anlotinib was tolerable.

## Table S1. Subgroup analyses of efficacy measures in Cohort A

| Subgroups | ORR, % (95%CI) | OR (95%CI) | *P* | Median PFS, months (95%CI) | HR (95%CI) | *P* | Median OS, months (95%CI) | HR (95%CI) | *P* |
| --- | --- | --- | --- | --- | --- | --- | --- | --- | --- |
| Age, years |  |  |  |  |  |  |  |  |  |
| <65 | 50.0 (28.2, 71.8) |  |  | 8.7 (7.1, NE) |  |  | NR (NE, NE) |  |  |
| ≥65 | 32.0 (15.0, 53.5) | 0.5 (0.1, 1.5) | 0.210 | NR (7.5, NE) | 0.7 (0.2, 2.1) | 0.474 | NR (NE, NE) | - | 0.348 |
| Sex |  |  |  |  |  |  |  |  |  |
| Male | 45.5 (28.1, 63.7) |  |  | 9.2 (7.3, NE) |  |  | NR (NE, NE) |  |  |
| Female | 28.6 (8.4, 58.1) | 0.5 (0.1, 1.9) | 0.281 | 8.7 (4.7, NE) | 1.2 (0.3, 3.9) | 0.821 | NR (NE, NE) | - | 0.515 |
| ECOG performance status |  |  |  |  |  |  |  |  |  |
| 0 | 20.0 (0.5, 71.6) |  |  | NR (7.3, NE) |  |  | NR (NE, NE) |  |  |
| 1 | 42.9 (27.7, 59.0) | 3.0 (0.3, 29.2) | 0.635 | 8.7 (7.3, NE) | 2.3 (0.3, 18.2) | 0.406 | NR (NE, NE) | - | 0.730 |
| Primary tumor location |  |  |  |  |  |  |  |  |  |
| Right colon | 40.0 (16.3, 67.7) |  |  | 8.7 (7.3, NE) |  |  | NR (NE, NE) |  |  |
| Left colon/rectum | 40.6 (23.7, 59.4) | 1.0 (0.3, 3.6) | 0.968 | 9.2 (7.3, NE) | 0.8 (0.3, 2.8) | 0.768 | NR (NE, NE) | - | 0.494 |
| No. of metastatic sites |  |  |  |  |  |  |  |  |  |
| 1 | 40.9 (20.7, 63.7) |  |  | 9.2 (7.3, NE) |  |  | NR (NE, NE) |  |  |
| 2 | 44.4 (21.5, 69.2) | 1.2 (0.3, 4.1) |  | 8.7 (7.5, NE) | 0.9 (0.2, 3.5) |  | NR (NE, NE) | - |  |
| ≥3 | 28.6 (3.7, 71.0) | 0.6 (0.1, 3.7) | 0.850 | 7.7 (0.1, NE) | 1.4 (0.4, 5.6) | 0.836 | NR (NE, NE) | - | 0.057 |
| Liver metastasis |  |  |  |  |  |  |  |  |  |
| Liver metastasis only | 40.9 (20.7, 63.7) |  |  | 9.2 (7.3, NE) |  |  | NR (NE, NE) |  |  |
| Liver metastasis and metastasis to other sites | 40.0 (21.1, 61.3) | 1.0 (0.3, 3.1) | 0.950 | 8.7 (7.1, NE) | 1.1 (0.3, 3.3) | 0.903 | NR (NE, NE) | - | 0.348 |
| Peritoneum or retroperitoneum metastasis |  |  |  |  |  |  |  |  |  |
| No | 41.3 (27.0, 56.8) |  |  | 8.7 (7.3, NE) |  |  | NR (NE, NE) |  |  |
| Yes | 0.0 (0.0, 97.5) | - | 1.000 | NR (NE, NE) | - | 0.436 | NR (NE, NE) | - | 0.883 |
| Lung metastasis |  |  |  |  |  |  |  |  |  |
| No | 40.0 (24.9, 56.7) |  |  | 8.7 (7.3, NE) |  |  | NR (NE, NE) |  |  |
| Yes | 42.9 (9.9, 81.6) | 1.1 (0.2, 5.7) | 1.000 | NR (7.7, NE) | 0.6 (0.1, 4.5) | 0.595 | NR (NE, NE) | - | 0.676 |
| Bone metastasis |  |  |  |  |  |  |  |  |  |
| No | 43.2 (28.4, 59.0) |  |  | 8.7 (7.3, NE) |  |  | NR (NE, NE) |  |  |
| Yes | 0.0 (0.0, 70.8) | - | 0.262 | NR (7.7, NE) | 0.8 (0.1, 6.5) | 0.840 | NR (NE, NE) | - | 0.794 |
| Lymph node metastasis |  |  |  |  |  |  |  |  |  |
| No | 40.0 (22.7, 59.4) |  |  | 9.2 (7.3, NE) |  |  | NR (NE, NE) |  |  |
| Yes | 41.2 (18.4, 67.1) | 1.1 (0.3, 3.5) | 0.937 | 8.7 (7.1, NE) | 1.6 (0.5, 5.1) | 0.399 | NR (NE, NE) | - | 0.184 |
| Surgery |  |  |  |  |  |  |  |  |  |
| No | 42.9 (21.8, 66.0) |  |  | 9.2 (7.5, NE) |  |  | NR (NE, NE) |  |  |
| Yes | 38.5 (20.2, 59.4) | 0.8 (0.3, 2.7) | 0.760 | 8.7 (7.3, NE) | 1.1 (0.4, 3.5) | 0.850 | NR (NE, NE) | - | 0.369 |
| Chemotherapy |  |  |  |  |  |  |  |  |  |
| No | 41.0 (25.6, 57.9) |  |  | 8.7 (7.3, NE) |  |  | NR (NE, NE) |  |  |
| Yes | 37.5 (8.5, 75.5) | 0.9 (0.2, 4.1) | >0.999 | NR (7.3, NE) | 0.7 (0.1, 5.2) | 0.692 | NR (NE, NE) | - | 0.651 |

## Table S2. Survival outcomes of the patients in Cohorts A and C

|  | Cohort A (N=47) | Cohort C (N=44) | Pancreatic cancer (N=32) |
| --- | --- | --- | --- |
| Progression-free survival |  |  |  |
| Median^*^ | 8.7 (7.3, NE) | 5.8 (4.8, 6.5) | 5.8 (3.7, 5.9) |
| 6 months | 91.8 (76.1, 97.4) | 35.3 (18.5, 52.6) | 25.1 (9.5, 44.5) |
| 12 months | 41.9 (19.2, 63.2) | 11.8 (2.4, 29.4) | 6.7 (0.5, 25.2) |
| Overall survival |  |  |  |
| Median^*^ | NR (NE, NE) | 11.4 (5.8, 19.3) | 11.4 (4.5, 19.3) |
| 6 months | 97.9 (85.8, 99.7) | 68.8 (49.4, 82.1) | 66.1 (43.4, 81.5) |
| 12 months | 97.9 (85.8, 99.7) | 47.2 (21.3, 69.5) | 35.3 (8.3, 64.6) |

Note^*^: Month, other data are expressed in % (95% CI).

## Table S3. Subgroup analyses of the ORR in patients with pancreatic cancer (N=32)

| Subgroups | ORR, % (95%CI) | OR (95%CI) | *P* |
| --- | --- | --- | --- |
| Age, years |  |  |  |
| <65 | 23.5 (6.8, 49.9) |  |  |
| ≥65 | 33.3 (11.8, 61.6) | 1.6 (0.3, 7.7) | 0.699 |
| Sex |  |  |  |
| Male | 30.0 (11.9, 54.3) |  |  |
| Female | 25.0 (5.5, 57.2) | 0.8 (0.2, 3.9) | >0.999 |
| ECOG performance status |  |  |  |
| 0 | 100.0 (15.8, 100.0) |  |  |
| 1 | 23.3 (9.9, 42.3) | - | 0.073 |
| Primary tumor location |  |  |  |
| Head | 23.1 (5.0, 53.8) |  |  |
| Body/Tail | 31.6 (12.6, 56.6) | 1.5 (0.3, 7.7) | 0.704 |
| No. of metastatic sites |  |  |  |
| 1 | 30.8 (14.3, 51.8) |  |  |
| 2 | 25.0 (0.6, 80.6) | 0.8 (0.1, 8.4) |  |
| ≥3 | 0.0 (0.0, 84.2) | - | >0.999 |
| Liver metastasis |  |  |  |
| Liver metastasis only | 30.8 (14.3, 51.8) |  |  |
| Liver metastasis and metastasis to other sites | 16.7 (0.4, 64.1) | 0.5 (0.0, 4.5) | 0.648 |
| Peritoneum or retroperitoneum metastasis |  |  |  |
| No | 28.1 (13.8, 46.8) |  |  |
| Yes | - | - | - |
| Lung metastasis |  |  |  |
| No | 26.7 (12.3, 45.9) |  |  |
| Yes | 50.0 (1.3, 98.7) | 2.8 (0.2, 49.3) | 0.490 |
| Bone metastasis |  |  |  |
| No | 29.0 (14.2, 48.0) |  |  |
| Yes | 0.0 (0.0, 97.5) | - | >0.999 |
| Lymph node metastasis |  |  |  |
| No | 32.1 (15.9, 52.4) |  |  |
| Yes | 0.0 (0.0, 60.2) | - | 0.304 |
| Surgery |  |  |  |
| No | 22.2 (6.4, 47.6) |  |  |
| Yes | 35.7 (12.8, 64.9) | 1.9 (0.4, 9.2) | 0.453 |
| Chemotherapy |  |  |  |
| No | 30.0 (14.7, 49.4) |  |  |
| Yes | 0.0 (0.0, 84.2) | - | >0.999 |
| CA19-9 |  |  |  |
| <37 U/mL | 0.0 (0.0, 84.2) |  |  |
| ≥37 U/mL | 30.0 (14.7, 49.4) | - | >0.999 |

## Table S4. Summary of treatment characteristics of the study patients in Cohorts A and C

|  | Cohort A (N=47) | Cohort C (N=44) |
| --- | --- | --- |
| Event leading to discontinuation of any treatment component | 2 (4.3) | 4 (9.1) |
| Discontinuation of anlotinib | 1 (2.1) | 3 (6.8) |
| Discontinuation of capecitabine | 0 (0.0) | 2 (4.5) |
| Discontinuation of oxaliplatin | 1 (2.1) | 0 (0.0) |
| Discontinuation of albumin-bound paclitaxel | 0 (0.0) | 1 (2.3) |
| Event leading to dose reductions | 10 (21.3) | 9 (20.5) |
| Anlotinib | 9 (19.2) | 7 (15.9) |
| Capecitabine | 4 (8.5) | 0 (0.0) |
| Oxaliplatin | 5 (10.6) | 0 (0.0) |
| Gemcitabine | 0 (0.0) | 6 (13.6) |
| Albumin-bound paclitaxel | 0 (0.0) | 5 (11.4) |

## Table S5. Summary of treatment-related adverse events in the safety population in Cohorts A (N=47) and C (N=44)

| **Cohort A** | | | **Cohort C** | | | |
| --- | --- | --- | --- | --- | --- | --- |
| **TRAEs** | **Any grades** | **Grade 3 or higher** | **TRAEs** | **Any grades** | **Grade 3 or higher** |  |
| Neutropenia | 20 (42.6) | 6 (12.8) | Neutropenia | 20 (45.5) | 8 (18.2) |  |
| Leucopenia | 18 (38.3) | 0 (0.0) | Leucopenia | 20 (45.5) | 6 (13.6) |  |
| Thrombocytopenia | 17 (36.2) | 5 (10.6) | Anemia | 15 (34.1) | 1 (2.3) |  |
| Palmar-plantar erythrodysaesthesia syndrome | 11 (23.4) | 1 (2.1) | Lymphocytopenia | 10 (22.7) | 2 (4.5) |  |
| Hypertension | 10 (21.3) | 3 (6.4) | Thrombocytopenia | 9 (20.5) | 4 (9.1) |  |
| Nausea | 9 (19.2) | 0 (0.0) | Hypertension | 7 (15.9) | 2 (4.5) |  |
| Fatigue | 8 (17.0) | 0 (0.0) | Body weight reduced | 6 (13.6) | 0 (0.0) |  |
| Diarrhea | 8 (17.0) | 0 (0.0) | γ-GT increased | 5 (11.4) | 3 (6.8) |  |
| Anemia | 8 (17.0) | 0 (0.0) | Abnormal hepatic function | 5 (11.4) | 0 (0.0) |  |
| Hyperlipidemia | 7 (14.9) | 0 (0.0) | Rash | 5 (11.4) | 0 (0.0) |  |
| Aspartate aminotransferase increased | 7 (14.9) | 0 (0.0) | Fatigue | 4 (9.1) | 1 (2.3) |  |
| Blood bilirubin increased | 7 (14.9) | 0 (0.0) | Alanine aminotransferase increased | 4 (9.1) | 0 (0.0) |  |
| Vomiting | 6 (12.8) | 0 (0.0) | Alopecia | 4 (9.1) | 0 (0.0) |  |
| Abnormal hepatic function | 5 (10.6) | 2 (4.3) | Hypertriglyceridemia | 3 (6.8) | 1 (2.3) |  |
| Thyroid stimulating hormone elevated | 5 (10.6) | 0 (0.0) | Constipation | 3 (6.8) | 0 (0.0) |  |
| Lymphocytopenia | 4 (8.5) | 0 (0.0) | Proteinuria | 3 (6.8) | 0 (0.0) |  |
| Decreased appetite | 4 (8.5) | 0 (0.0) | Hyponatremia | 3 (6.8) | 0 (0.0) |  |
| Fever | 3 (6.4) | 1 (2.1) | Oral ulcer | 3 (6.8) | 0 (0.0) |  |
| Blood pressure increased | 3 (6.4) | 1 (2.1) | Vomiting | 3 (6.8) | 0 (0.0) |  |
| Alanine aminotransferase increased | 3 (6.4) | 0 (0.0) | Aspartate aminotransferase increased | 3 (6.8) | 0 (0.0) |  |
| Body weight decreased | 3 (6.4) | 0 (0.0) | Indigestion | 3 (6.8) | 0 (0.0) |  |
| Blood triglyceride increased | 3 (6.4) | 0 (0.0) | Thyroid stimulating hormone elevated | 3 (6.8) | 0 (0.0) |  |
| Hypokalemia | 2 (4.3) | 1 (2.1) | Blood alkaline phosphatase increased | 3 (6.8) | 0 (0.0) |  |
| Ulcer | 1 (2.1) | 1 (2.1) | Bone marrow suppression | 2 (4.5) | 2 (4.5) |  |
| **-** | - | - | Fever | 2 (4.5) | 1 (2.3) |  |
| **-** | - | - | Intestinal fistulation | 1 (2.3) | 1 (2.3) |  |
| **-** | - | - | Arterial thrombosis | 1 (2.3) | 1 (2.3) |  |
| **-** | - | - | Febrile neutropenia | 1 (2.3) | 1 (2.3) |  |
| **-** | - | - | Hepatic hemorrhage | 1 (2.3) | 1 (2.3) |  |
| **-** | - | - | Coagulation abnormalities | 1 (2.3) | 1 (2.3) |  |
| **During induction therapy** |  |  |  |  |  |  |
| Neutropenia | 20 (42.6) | 6 (12.8) | Neutropenia | 20 (45.5) | 8 (18.2) |  |
| Leucopenia | 18 (38.3) | 0 (0.0) | Leucopenia | 20 (45.5) | 6 (13.6) |  |
| Thrombocytopenia | 16 (34.0) | 4 (8.5) | Anemia | 13 (29.6) | 1 (2.3) |  |
| Hypertension | 10 (21.3) | 3 (6.4) | Lymphocytopenia | 9 (20.5) | 2 (4.5) |  |
| Nausea | 9 (19.2) | 0 (0.0) | Thrombocytopenia | 8 (18.2) | 4 (9.1) |  |
| Diarrhea | 8 (17.0) | 0 (0.0) | Hypertension | 7 (15.9) | 2 (4.5) |  |
| Palmar-plantar erythrodysaesthesia syndrome | 7 (14.9) | 1 (2.1) | Body weight reduced | 6 (13.6) | 0 (0.0) |  |
| Fatigue | 7 (14.9) | 0 (0.0) | γ-GT increased | 5 (11.4) | 3 (6.8) |  |
| Anemia | 7 (14.9) | 0 (0.0) | Rash | 5 (11.4) | 0 (0.0) |  |
| Aspartate aminotransferase increased | 7 (14.9) | 0 (0.0) | Fatigue | 4 (9.1) | 1 (2.3) |  |
| Blood bilirubin increased | 7 (14.9) | 0 (0.0) | Alanine aminotransferase increased | 4 (9.1) | 0 (0.0) |  |
| Vomiting | 6 (12.8) | 0 (0.0) | Abnormal hepatic function | 4 (9.1) | 0 (0.0) |  |
| Abnormal hepatic function | 5 (10.6) | 2 (4.3) | Alopecia | 4 (9.1) | 0 (0.0) |  |
| TSH elevated | 5 (10.6) | 0 (0.0) | Hypertriglyceridemia | 3 (6.8) | 1 (2.3) |  |
| Hyperlipidemia | 4 (8.5) | 0 (0.0) | Constipation | 3 (6.8) | 0 (0.0) |  |
| Lymphocytopenia | 4 (8.5) | 0 (0.0) | Oral ulcer | 3 (6.8) | 0 (0.0) |  |
| Decreased appetite | 4 (8.5) | 0 (0.0) | Vomiting | 3 (6.8) | 0 (0.0) |  |
| Fever | 3 (6.4) | 1 (2.1) | Aspartate aminotransferase increased | 3 (6.8) | 0 (0.0) |  |
| Blood pressure increased | 3 (6.4) | 1 (2.1) | Indigestion | 3 (6.8) | 0 (0.0) |  |
| Alanine aminotransferase increased | 3 (6.4) | 0 (0.0) | Blood alkaline phosphatase increased | 3 (6.8) | 0 (0.0) |  |
| Body weight decreased | 3 (6.4) | 0 (0.0) | Bone marrow suppression | 2 (4.5) | 2 (4.5) |  |
| Blood triglycerides increased | 3 (6.4) | 0 (0.0) | Fever | 2 (4.5) | 1 (2.3) |  |
| Hypokalemia | 2 (4.3) | 1 (2.1) | Intestinal fistulation | 1 (2.3) | 1 (2.3) |  |
| Ulcer | 1 (2.1) | 1 (2.1) | Febrile neutropenia | 1 (2.3) | 1 (2.3) |  |
| - | - | - | Hepatic hemorrhage | 1 (2.3) | 1 (2.3) |  |
| - | - | - | Coagulation abnormalities | 1 (2.3) | 1 (2.3) |  |
| **During maintenance therapy** |  |  |  |  |  |  |
| Thrombocytopenia | 5 (10.6) | 1 (2.1) | Arterial thrombosis | 1 (2.3) | 1 (2.3) |  |
| Palmar-plantar erythrodysaesthesia syndrome | 4 (8.5) | 0 (0.0) | Hypertension | 1 (2.3) | 1 (2.3) |  |
| Hyperlipidemia | 3 (6.4) | 0 (0.0) | - | - | - |  |
| Neutropenia | 2 (4.3) | 1 (2.1) | - | - | - |  |

Note: Listed in descending order of frequency are all treatment-related adverse events (TRAEs) that occurred in ≥5% of the patients or any grade 3 or higher TRAEs during the trial period or within the 30 days thereafter (within 90 days for serious events), regardless of attribution to any trial regimen by an investigator.

## Table S6. Summary of serious adverse events and those leading to hospitalization or extended hospitalization in the study patients

| Events | Patients (N=91) |
| --- | --- |
| **Serious adverse events** |  |
| Abnormal hepatic function | 3 (3.3) |
| Thrombocytopenia | 3 (3.3) |
| Nausea | 2 (2.2) |
| COVID-19 | 1 (1.1) |
| Epistaxis | 1 (1.1) |
| Viral abdominal infection | 1 (1.1) |
| Intestinal fistula | 1 (1.1) |
| Cerebral hemorrhage | 1 (1.1) |
| Biliary tract infection | 1 (1.1) |
| Biliary obstruction | 1 (1.1) |
| Hypokalemia | 1 (1.1) |
| Arterial thrombosis | 1 (1.1) |
| Cachexia | 1 (1.1) |
| Fever | 1 (1.1) |
| Febrile infection | 1 (1.1) |
| Febrile neutropenia | 1 (1.1) |
| Abdominal pain | 1 (1.1) |
| Hepatic hemorrhage | 1 (1.1) |
| Hepatic abscess | 1 (1.1) |
| Infectious pneumonia | 1 (1.1) |
| Perianal abscess | 1 (1.1) |
| Bone marrow suppression | 1 (1.1) |
| Coronavirus infection | 1 (1.1) |
| Disease progression | 1 (1.1) |
| Glycosuria | 1 (1.1) |
| Sepsis | 1 (1.1) |
| Upper respiratory infection | 1 (1.1) |
| Upper gastrointestinal bleed | 1 (1.1) |
| Renal infection | 1 (1.1) |
| Retinal hemorrhage | 1 (1.1) |
| Pancreatitis | 1 (1.1) |
| Dizziness | 1 (1.1) |
| **Serious adverse events leading to hospitalization or extended hospitalization** |  |
| Abnormal hepatic function | 3 (3.3) |
| Thrombocytopenia | 3 (3.3) |
| Nausea | 2 (2.2) |
| COVID-19 | 1 (1.1) |
| Viral abdominal infection | 1 (1.1) |
| Cerebral hemorrhage | 1 (1.1) |
| Biliary tract infection | 1 (1.1) |
| Biliary obstruction | 1 (1.1) |
| Arterial thrombosis | 1 (1.1) |
| Fever | 1 (1.1) |
| Febrile infection | 1 (1.1) |
| Febrile neutropenia | 1 (1.1) |
| Abdominal pain | 1 (1.1) |
| Hepatic abscess | 1 (1.1) |
| Infectious pneumonia | 1 (1.1) |
| Perianal abscess | 1 (1.1) |
| Bone marrow suppression | 1 (1.1) |
| Coronavirus infection | 1 (1.1) |
| Glycosuria | 1 (1.1) |
| Sepsis | 1 (1.1) |
| Upper respiratory infection | 1 (1.1) |
| Retinal hemorrhage | 1 (1.1) |
| Pancreatitis | 1 (1.1) |
| Dizziness | 1 (1.1) |

## Table S7. Summary of perioperative treatment-emergent adverse events in patients who underwent surgical resection

| Treatment emergent adverse events | Patients (N=15) |
| --- | --- |
| Anemia | 4 (26.7) |
| Neutropenia | 3 (20.0) |
| Leukopenia | 2 (13.3) |
| Lymphopenia | 2 (13.3) |
| Hypoalbuminemia | 1 (6.7) |
| Hypoproteinemia | 1 (6.7) |
| Hypokalemia | 1 (6.7) |
| Abnormal hepatic function | 1 (6.7) |
| Hypercholesterolemia | 1 (6.7) |
| Proteinuria | 1 (6.7) |
| Aspartate aminotransferase increased | 1 (6.7) |
| Blood triglycerides increased | 1 (6.7) |
| Blood alkaline phosphatase increased | 1 (6.7) |

Note: Listed in descending order of frequency are all liver resection-related complications for 15 patients from the end of systemic treatment until the 30-day safety follow-up period post-surgery.
