# Supplementary material for: Anlotinib plus chemotherapy as a first-line treatment for gastrointestinal cancer patients with unresectable liver metastases: a multicohort, multicenter, exploratory trial
Source: Signal Transduct Target Ther. 2024 Dec 9;9:344. doi: 10.1038/s41392-024-02051-4 (PMC11625826; doi:10.1038/s41392-024-02051-4)
Supplement: Supplementary file 2 — Study Protocol [file 41392_2024_2051_MOESM2_ESM.docx]

A Multi-cohort, multi-center and exploratory clinical study

of anlotinib combined with chemotherapy in the treatment of gastrointestinal cancer with liver metastases

| Protocol Number: | ALTER-G-001 |
| --- | --- |
| Version No.: | 2.0 |
| Version Date: | December 06, 2022 |

| Leading site: | Ruijin Hospital affiliated to Shanghai Jiao Tong University School of Medicine |
| --- | --- |
| Principal Investigator: | Jun Zhang |
| Sponsor: | Chia Tai Tianqing Pharmaceutical Group Co., Ltd. |

This document contains important confidential commercial information that is the property of Chia Tai Tianqing Pharmaceutical Group CO., LTD and shall not be disclosed unless such disclosure is required by current laws or regulations. In any event, all persons to whom this information is received shall be informed of confidentiality requirements and shall not further disclose this information. This disclosure restriction also applies to all future documents supplied and annotated as confidential.

Protocol Signature Page

Investigator Signature

I will be in accordance with China GCP stipulates that the investigator's responsibilities shall be earnestly performed, and the clinical study shall be personally participated in or directly guided. I have read and confirmed this protocol (Protocol No.: ALTER-G-001; Version No.: _2.0_; Version Date: December 06, 2022), and agrees with the scientificity and ethics of this protocol. We will comply with the laws of China, Declaration of Helsinki, China GCP and the study protocol stipulate to fulfill the relevant responsibilities, and only modify the protocol after notifying the sponsor, which can be implemented only after being approved by the ethics committee, unless the measures must be taken to protect the safety, rights and interests of the subjects.

We will keep this study protocol confidential.

| Study Site: Ruijin Hospital affiliated to Shanghai Jiao Tong University School of Medicine |
| --- |
|  |

| Jun Zhang | |  |  |
| --- | --- | --- | --- |
| Principal Investigator (Print) | Principal Investigator (Signature) | Date of Signature (DD/MMM/YYYY) |  |

Protocol Version Update Log

| Version No. | Version Date |
| --- | --- |
| 1.0 | April 1, 2021 |
| 1.1 | July 30, 2021 |
| 2.0 | December 6, 2022 |
|  |  |

CONTENTS

[List of Abbreviations 3](#_Toc178516209)

[Protocol Synopsis 4](#_Toc178516210)

[1.1 Flow Chart of Clinical Trial 11](#_Toc178516211)

[1.2 Study Design Planning Diagram 18](#_Toc178516212)

[2 Study background 18](#_Toc178516213)

[2.1 Study Feasibility 18](#_Toc178516214)

[2.2 Anlotinib Hydrochloride 20](#_Toc178516215)

[2.2.1 Basic information and pharmacological mechanism of Anlotinib Hydrochloride 20](#_Toc178516216)

[2.2.2 Preclinical Studies of Anlotinib Hydrochloride 20](#_Toc178516217)

[2.2.3 Phase I clinical trials 21](#_Toc178516218)

[2.3 Feasibility of Anlotinib Hydrochloride in the Treatment of Gastrointestinal Tumors 22](#_Toc178516219)

[2.3.1 Studies of anlotinib hydrochloride in patients with advanced colorectal cancer 22](#_Toc178516220)

[2.3.2 Study of anlotinib in patients with advanced esophageal squamous cell carcinoma 24](#_Toc178516221)

[3 Study objectives 28](#_Toc178516222)

[3.1 Primary Study Objective 28](#_Toc178516223)

[3.2 Secondary study objectives 28](#_Toc178516224)

[3.3 Exploratory objective (leading site) 28](#_Toc178516227)

[4 Study plan 28](#_Toc178516228)

[4.1 Study design 28](#_Toc178516229)

[4.2 Study Duration 28](#_Toc178516230)

[4.3 Study Population 28](#_Toc178516231)

[4.3.1 Inclusion criteria 28](#_Toc178516232)

[4.3.2 Exclusion criteria 30](#_Toc178516233)

[4.3.3 Withdrawal criteria 31](#_Toc178516234)

[4.3.4 Removal criteria 31](#_Toc178516235)

[4.3.5 Discontinuation criteria 31](#_Toc178516236)

[5 Study drugs 32](#_Toc178516237)

[5.1 Study drugs 32](#_Toc178516238)

[5.2 Dosing Regimen 32](#_Toc178516239)

[5.2.1 Dosing Protocol design 32](#_Toc178516240)

[5.2. 2 Method of Administration 34](#_Toc178516241)

[5.3 Dose modification and discontinuation 35](#_Toc178516242)

[5.3.1 Anlotinib Hydrochloride 35](#_Toc178516243)

[5.3.2 Dose modification principles for oxaliplatin 37](#_Toc178516244)

[5.3.3 Dose modification principles for capecitabine 38](#_Toc178516245)

[5.3.4 Dose modification principles for paclitaxel 39](#_Toc178516246)

[5.3.5 Dose modification principles for docetaxel 40](#_Toc178516247)

[5.3.6 Dose modification principles for cisplatin 40](#_Toc178516248)

[5.3.7 Dose modification principles for other chemotherapy drugs 40](#_Toc178516249)

[5.4 Concomitant medications and treatments 40](#_Toc178516250)

[5.5 Prohibited Drugs or Concomitant medications allowing with caution 40](#_Toc178516251)

[6 Observation items 42](#_Toc178516252)

[6.1 Before study initiation 42](#_Toc178516253)

[6.2 During the trial 42](#_Toc178516254)

[6.3 End of Treatment and follow-up 42](#_Toc178516255)

[6.3.1 Out-of -group visit 42](#_Toc178516256)

[6.3.2 Safety Follow-up Visit 43](#_Toc178516257)

[6.3.3 Survival Follow-up 43](#_Toc178516258)

[7 Efficacy evaluation 43](#_Toc178516259)

[7.1 Tumor Evaluation Criteria 44](#_Toc178516260)

[7.2 Primary end point 45](#_Toc178516261)

[7.3 Secondary end points 45](#_Toc178516262)

[8 Safety evaluation 46](#_Toc178516263)

[8.1 Definitions 46](#_Toc178516264)

[8.2 Recording and assessing of adverse events 47](#_Toc178516265)

[8.3 Reporting System and Procedures for Serious Adverse Events 48](#_Toc178516266)

[8.4 Symptomatic treatment of common adverse reactions Advice 48](#_Toc178516267)

[9 Data Management and Statistical Analysis 51](#_Toc178516268)

[9.1 Case Report Form 51](#_Toc178516269)

[9.2 Database establishment 51](#_Toc178516270)

[9.3 Data Lock 52](#_Toc178516271)

[9.4 Selection of Statistical Analysis Data 52](#_Toc178516272)

[9.5 Dropouts 52](#_Toc178516273)

[9.6 Statistical Analysis Plan 52](#_Toc178516274)

[9.6.1 Analysis of Subject Characteristics 52](#_Toc178516275)

[9.6.2 Efficacy Analysis 52](#_Toc178516276)

[9.7 Estimation of Sample Size 53](#_Toc178516277)

[10 Preservation and intellectual property rights of data collected in the study 54](#_Toc178516278)

[11 Responsibilities of sponsor and Investigator 54](#_Toc178516279)

[11.1 Sponsor 54](#_Toc178516280)

[11.2 Investigator 54](#_Toc178516281)

[12 Ethical Guidelines and Informed Consent Form 55](#_Toc178516282)

[13 Quality Control and Quality Assurance 55](#_Toc178516283)

[14 Study site and personnel 55](#_Toc178516284)

[14.1 Study Site, Site Number, and Principal Investigator (Sort by Site Number) 55](#_Toc178516285)

[14.2 Leading Site and principle investigator 56](#_Toc178516286)

[14.3 Sponsor and responsible personnel 56](#_Toc178516287)

[15 Discussion, Approval and Modification of Study Protocol 56](#_Toc178516288)

[16 Test Summary 56](#_Toc178516289)

[17 REFERENCES 57](#_Toc178516290)

**List of Abbreviations**

| ACEI | | : | | Angiotensin-converting enzyme inhibitor | |
| --- | --- | --- | --- | --- | --- |
| ADR | | : | | Adverse drug reaction | |
| AE | | : | | Adverse events | |
| AFP | | : | | Alpha-fetoprotein | |
| ALT | | : | | Alanine aminotransferase (Glutamic-pyruvic transaminase) | |
| ALP | | : | | Alkaline phosphatase | |
| ANC | | : | | Neutrophils | |
| APTT | | : | | Activated part Coagulation Active enzyme time | |
| ARB | | : | | Angiotensin II Receptor Antagonists | |
| AUC | | : | | Area under the curve | |
| AST | | : | | Aspartate aminotransferase (AST) | |
| BID | | : | | Twice daily | |
| BUN | | : | | Urea nitrogen | |
| Ca | | : | | Calcium | |
| CEA | | : | | Carcinoembryonic antigen | |
| CFDA | | : | | China Food and Drug Administration | |
| CCR | | : | | Creatinine clearance | |
| Cl | | : | | Chlorine | |
| Cr | | : | | Creatinine | |
| CR | | : | | Complete response | |
| CRF | | : | | Case Report Form | |
| CT | | : | | Computed tomography | |
| CYP450 | | : | | Cytochrome P450 | |
| DBIL | | : | | Direct bilirubin | |
| DCR | | : | | Disease control rate | |
| DoR | | : | | Duration of Response | |
| DLT | | : | | Dose limiting toxicity | |
| ECOG PS | | : | | Performance status scoring criteria | |
| EDC | | : | | Electronic entry system | |
| EGFR | | : | | Epidermal growth factor receptor | |
| FIB | | : | | Fibrinogen | |
| FAS | | : | | Full Analysis Set | |
| FISH | | : | | Fluorescence in situ hybridization | |
| Glu | | : | | Glucose | |
| GCP | | : | | Good Clinical Practice | |
| G-CSF | | : | | Granulocyte colony stimulating factor | |
| GGT | | : | | Glutamyl transpeptidase | |
| GM-CSF | | : | | Granulocyte-macrophage colony-stimulating factor | |
| Hb | | : | | Hemoglobin | |
| HER2 | | : | | Human epidermal growth factor receptor-2 | |
| HMG-COA | | : | | 3-hydroxy-3-methylpentanedioyl-CoA reductase | |
| HB | | : | | Hemoglobin | |
| HR | | : | | Hazard ratio | |
| HIV | | : | | HIV | |
| IC50 | | : | | Half-maximal inhibitory concentration | |
| INR | | : | | International Standardization | |
| ITT | | : | | Intent to treat set | |
| ICH | | : | | Immunohistochemical Staining | |
| K | | : | | Potassium | |
| LD _50_ | | : | | Median lethal dose | |
| MRI | | : | | NMR | |
| Na | | : | | Sodium | |
| NCI-CTC | | : | | National Cancer Institute Common Toxicity Criteria | |
| ORR | | : | | Objective response rate | |
| OS | | : | | Overall survival | |
| PFS | | : | | Progression-free survival | |
| PD | | : | | Disease progression | |
| PDGFR | | : | | Platelet-derived growth factor receptor | |
| PLT | | : | | Platelets | |
| PO | | : | | Oral | |
| PR | | : | | Partial response | |
| PRO | | : | | Protein | |
| PPS | | : | | Per Protocol Set | |
| PT | | : | | Coagulation Zymogen time | |
| Q3W | | : | | Every 3 weeks | |
| Qd | | : | | Once daily | |
| RBC | | : | | Red blood cell | |
| RECIST | | : | | Response Evaluation Criteria in Solid Tumors | |
| NMPA | | : | | China Food and Drug Administration | |
| SAE | | : | | Serious adverse event | |
| SAS | | : | | Safety Analysis Set | |
| SD | | : | | Stable disease | |
| SUSAR | | : | | Suspected and unexpected serious adverse reactions | |
| TACE | | : | | Transcatheter arterial chemoembolization | |
| TBIL | | : | | Total bilirubin | |
| TT | | : | | Coagulation Enzyme Time | |
| ULN | | : | | Upper limit of normal | |
| VEGF | | : | | Vascular endothelial growth factor | |
| VEGFR | | : | | Vascular endothelial growth factor receptor | |
| WBC | | : | | Leucocyte | |
|  | |  | |  | |

**Protocol Synopsis**

| **Study title** | | A multi-cohort, multi-center, exploratory clinical study of anlotinib combined with chemotherapy in the treatment of gastrointestinal cancer with liver metastases |
| --- | --- | --- |
| **Protocol No.** | | ALTER-G-001 |
| **Version and date** | | 2.0/ December 6, 2022 |
| **Principal Center** | | Ruijin Hospital affiliated to Shanghai Jiao Tong University School of Medicine |
| **Co-organizer** | | Chia Tai Tianqing Pharmaceutical Group Co., Ltd. |
| **Nature of Study** | | Investigator-initiated exploratory studies |
| **Study subjects** | | Patients with untreated advanced colorectal cancer, esophageal squamous cell carcinoma, or other gastrointestinal cancers with unresectable liver metastases. |
| **Study objectives** | | To explore the efficacy and safety of anlotinib combined with chemotherapy in the first-line and maintenance therapy of gastrointestinal cancer patients with unresectable liver metastases |
| **Evaluation Criteria** | | Adverse events are evaluated using NCI CTC AE V5.0 criteria  Efficacy is evaluated using RECIST 1.1 criteria |
| **Study**  **Purpose** | Primary objective | objective response rate ( ORR, CR + PR) |
|  | Secondary objectives | (1) Progression-free survival (PFS);  (2) Disease control rate (DCR, CR + PR + SD);  (3) Duration of response (DoR);  (4) Overall survival (OS);  (5) Conversion rate of liver metastases;  (6) Safety |
| **Study Design** | | Multi-cohort, multi-center clinical trial |
| **Sample Size** | | 116 cases |
| **Study Cohort** | | Participants are divided into 3 cohorts according to different primary tumor types:  Cohort A: colorectal cancer with liver metastasis, 45 cases in total;  Cohort B: esophageal squamous cell carcinoma with liver metastasis, 31 cases in total;  Cohort C: other gastrointestinal tumors with liver metastases, 40 cases in total |
| **Estimation of sample size** | | This is a single-arm exploratory study, with ORR as the primary study endpoint; α=0.05, β=0.2, and NCSS&PASS 15.0 software are used for calculation.  Cohort A: Colorectal cancer with liver metastases  In the NO16966^[1]^ study, the ORR was 47% in patients with advanced colorectal cancer treated with CAPEOX/FOLFOX4 in combination with bevacizumab as first-line therapy. Based on the preliminary study results of anlotinib in the treatment of patients with colorectal cancer and current clinical practice requirements, assuming that first-line and maintenance therapy of anlotinib in combination with CAPEOX regimen could improve the ORR from 47% to 70%, a sample size of 36 patients is required. A total of 45 patients are required to be enrolled in this cohort with 20% drop-out rate.  Cohort B: Esophageal squamous cell carcinoma with liver metastasis  The ORR of paclitaxel combined with cisplatin as first-line treatment in patients with advanced esophageal squamous cell carcinoma was 48.6%. Based on the preliminary study results of anlotinib in the treatment of patients with advanced esophageal squamous cell carcinoma and current clinical practice requirements, assuming that first-line and maintenance therapy with anlotinib in combination with paclitaxel and cisplatin could increase the ORR from 48% to 75%, a sample size of 25 patients is required. A total of 31 patients are required to be enrolled in this cohort with 20% drop-out rate.  Cohort C: Other gastrointestinal tumor with liver metastasis  The ORR for the first-line standard chemotherapy regimen in the treatment of other advanced gastrointestinal malignancies (e.g., gastric cancer, biliary tract cancer cancer, pancreatic cancer, etc.) were 19.4%-47.8%. Based on the preliminary study results of anlotinib in esophageal and colorectal cancer and current clinical practice requirements, assuming that anlotinib in combination with standard chemotherapy regimen for first-line and maintenance therapy could increase the ORR from 25% to 47%, a sample size of 33 patients is required. A total of 40 patients are required to be enrolled in this cohort with 20% drop-out rate. |
| **Statistical Methods** | | - Selection of statistical analysis data   **Full Analysis Set:** Enrolled patients who used the drug at least once according to the intention-to-treat (ITT) principle.  **Per Protocol Set:** Patients who have completed the treatment for more than 6 cycles (including 6 cycles), comformed to the trial protocol, have good compliance, have not used prohibited drugs during the trial, and complete the content specified in the CRF.  **Safety Analysis Set:** All patients who used the experimental drug at least once with safety record.   - **Statistical analysis plan**   **Efficacy Analysis:** For PFS, DoR and OS, median time will be estimated using Kaplan-Meier method and event and its 95% confidence interval will be presented. Disease control rate (DCR = CR + PR + SD), objective response rate (ORR = CR + PR), and liver metastasis conversion rate is calculated using Fisher's exact probability.  **Safety analysis:** Descriptive statistical analysis should be the main method to describe AEs in this trial. Laboratory test results describe the conditions that are normal before the test but abnormal after treatment and the relationship with the test drug in case of abnormal changes. |
| **Dosing Regimen** | | **Cohort A: colorectal Cancer with liver metastases (anlotinib in combination with CAPEOX regimen):**   - Anlotinib: 12 mg, po, qd, d1-d14 of each cycle; - Oxaliplatin: 130 mg/m^2^, IV infusion > 2h, d1 of each cycle; - Capecitabine: 850 mg/m^2^, oral, twice a day on d1-d14 of each cycle. - Three weeks is a treatment cycle of the above drugs, and tumor efficacy is evaluated at the end of cycle 2, 4 and 6; if necessary, the MDT team is required to assess the possibility of surgical resection of liver metastases. If surgical resection is possible, anlotinib and chemotherapeutic drugs should be discontinued (4 weeks before surgery or determined by the investigator). In case of the liver metastases is unresectable after 6 cycles of treatment, patients with CR/PR/SD were switched to maintenance therapy with anlotinib (12 mg, po, qd, d1-d14, Q3W) combined with metronomic capecitabine chemotherapy (500 mg, po, bid, daily) until disease progression or intolerance.   **Cohort B: Esophageal squamous cell carcinoma with liver metastasis (anlotinib plus TP regimen):**   - Anlotinib: 12 mg, po, qd, d1-d14 of each cycle; - Paclitaxel: 135 mg/m^2^, IV infusion > 2h, d1 of each cycle; or Docetaxel: 75 mg/m^2^ IV infusion for 1h, d1 in each cycle; - Cisplatin: 60-75 mg/m^2^ IV infusion, on d1 of each cycle, or divided into d1-d3. - Three weeks is a treatment cycle of the above drugs, and tumor efficacy is evaluated at the end of cycle 2, 4 and 6; if necessary, MDT team is required to assess the possibility of surgical resection of liver metastases. If surgical resection is possible, anlotinib and chemotherapeutic drugs should be discontinued (4 weeks before surgery or determined by the investigator). In case of the liver metastases is unresectable after 6 cycles of treatment, patients with CR/PR/SD were switched to maintenance therapy with anlotinib (12 mg, po, qd, d1-d14, Q3W) combined with metronomic capecitabine chemotherapy (500 mg, po, bid, daily) until disease progression or intolerance.   **Cohort C: Other gastrointestinal cancers with liver metastasis (anlotinib in combination with standard first-line chemotherapy)**   - Anlotinib: 12 mg, po, qd, d1-d14 of each cycle; - first-line standard chemotherapy regimens are determined by the investigator (3-week chemotherapy regimen is selected);   the above drugs are 3-week treatment cycles, and tumor efficacy is evaluated at the end of cycle 2, 4, and 6; if necessary, the MDT team is required to assess the possibility of surgical resection of liver metastases. If surgical resection is possible, anlotinib and chemotherapeutic drugs should be discontinued (4 weeks before surgery or determined by the investigator). In case of the liver metastases is unresectable after 6 cycles of treatment, patients with CR/PR/SD were switched to maintenance therapy with anlotinib (12 mg, po, qd, d1-d14, Q3W) combined with chemotherapy (500 mg, po, bid, daily, metronomic capecitabine chemotherapy is the preferred chemotherapy agent) until disease progression or intolerance. |
| **Principal Investigator** | | Jun Zhang |
| **Principal Center** | | Ruijin Hospital affiliated to Shanghai Jiao Tong University School of Medicine |
| **Inclusion Criteria:**  Patients who meet all the following inclusion criteria can be included in this trial:   1. The patient voluntarily joined the study, signed the informed consent form, had good compliance and cooperated with the follow-up; 2. Male or female patients aged 18-75 years; 3. Cohort A: Patients histopathologically or cytologically confirmed colorectal cancer at stage IV (T_Any_N_Any_M1) with with unresectable liver metastases;   Cohort B: Patients histopathologically or cytologically confirmed esophageal squamous cell carcinoma at stage IVb (T_Any_N_Any_M1) with liver unresectable metastases (excluding mixed adenosquamous carcinoma);  Cohort C: Patients histopathologically or cytologically confirmed other gastrointestinal tumors with liver metastases (excluding gastrointestinal stromal tumors, neuroendocrine tumors and other malignant tumors of non-glandular epithelial origin);   1. No previous systemic therapy, including chemotherapy, targeted therapy and immunotherapy;   Note: Patients who relapsed after receiving neoadjuvant (radio)chemotherapy plus radical surgery for more than 6 months, or relapsed after receiving adjuvant (radio)chemotherapy or radical concurrent chemoradiotherapy for more than 6 months were eligible;   1. At least one measurable lesion in the liver metastases according to the Response Evaluation Criteria in Solid Tumors (RECIST) Version 1.1, and the target lesions must contain liver metastases. The selected target lesions have not received local therapy such as radiotherapy or TACE within 6 months;   Note: Lesions in the area of previous local treatment can also be selected as target lesions if progression is confirmed and meet RECIST 1.1 criteria;   1. Palliative therapy for localized lesions (non-target lesions) should be completed for> 2 weeks; 2. ECOG PS score: 0 ~ 1; 3. Life expectancy of more than 3 months; 4. Adequate organ and bone marrow function, defined as meeting the following criteria (within 7 days prior to enrollment): 5. The blood routine examination criteria shall meet:  - Hemoglobin level (HB) ≥ 90 g/L (no blood transfusion within 14 days); - Absolute neutrophil count (ANC) ≥ 1.5 × 10^9^ /L; - Platelet count (PLT) ≥ 90 × 10^9^ /L.  1. Biohemical tests must meeting the following criteria:  - Serum total bilirubin (TBIL) ≤ 1.5 × upper limit of normal (ULN); - Alanine aminotransferase (ALT) and aspartate aminotransferase (AST) ≤ 5 ULN; - Serum creatinine (Cr) ≤ 1.5 ULN or creatinine clearance (CCr) ≥ 60ml/min; (Cockcroft-Gault formula)  1. Adequate coagulation function defined as international normalized ratio (INR) or prothrombin time (PT) ≤ 1.5 × ULN; 2. Females of childbearing potential are required to use adequate contraception and avoid breastfeeding from screening through 3 months after discontinuation of study treatment. Have a negative pregnancy test prior to initiation of dosing or meet one of the following criteria to demonstrate absence of risk of pregnancy: 3. Postmenopausal is defined as age greater than 50 years and amenorrhea for at least 12 months after stopping all exogenous hormone replacement therapy; 4. Women younger than 50 years of age may also be considered postmenopausal if they have been amenorrheic for 12 months or more after stopping all exogenous hormone therapy and their luteinizing hormone (LH) and follicle stimulating hormone (FSH) levels are within the laboratory's postmenopausal reference range; 5. Undergo irreversible sterilization, including hysterectomy, oophorectomy, or salpingectomy, except tubal ligation.   Males must agree to use an adequate method of contraception or have been surgically sterile during the trial and for 8 weeks after receiving trial drug.  **Exclusion Criteria**:  Patients with any of the following will not be enrolled in the study:   1. Patients with active bleeding within 2 months from the primary lesion and/or metastases; 2. Hyperactive/venous thrombosis within 6 months, such as cerebrovascular accident (including temporary ischemic attack), deep vein thrombosis and pulmonary embolism; 3. For patients receiving thrombolytic or anticoagulant therapy such as warfarin, heparin or their analogues, low-dose heparin (daily dose of 6,000 ~ 12,000 U for adults) or low-dose aspirin (daily dose ≤ 100 mg) for preventive purposes is allowed provided INR ≤ 1.5 × ULN; 4. Patients with gastrointestinal diseases with bleeding tendency (such as active gastrointestinal ulcer) or investigators judgment that may cause gastrointestinal bleeding, perforation or obstruction, or patients with established fistula; 5. Receiving radiotherapy or surgery within 30 days, except biopsy and palliative treatment for non-target lesions (note: patients undergoing non-major trauma surgery can receive this protocol as early as 15 days after surgery if they recover quickly after surgery and can use anti-angiogenic drugs as assessed by the investigator); 6. Patients with HER2-positive gastric adenocarcinoma; 7. a history of immunodeficiency, including HIV positive or other acquired or congenital immunodeficiency disorders, or a history of organ transplantation; 8. Patients with brain metastasis and/or leptomeningeal metastasis; for subjects with neurological symptoms, CT/MRI should be performed to rule out brain metastasis; 9. Patients with any severe and/or uncontrolled disease including:  - Patients with hypertension that is not well controlled by single antihypertensive drug therapy (systolic blood pressure ≥ 150 mmHg, diastolic blood pressure ≥ 100 mmHg); or use two or more antihypertensive drugs to control blood pressure; - Patients with acute myocardial infarction, malignant arrhythmia (including QT interval > 450ms for males and > 470ms for females) and grade II and above congestive heart failure (New York Heart Association (NYHA) classification); - Active or uncontrolled serious infection (NCI-CTC AE Grade ≥ 2 infection); - Liver disease such as cirrhosis, decompensated liver disease, active hepatitis or chronic hepatitis (HBV-DNA > 1000 IU/mL) requiring antiviral therapy; - Poor glycemic control in diabetic patients (fasting blood glucose > 10mmol/L); - Urinalysis showed urine protein ≥ + +, and confirmed 24-hour urine protein > 1.0 g;  1. Patients with clinically significant ascites, including any ascites that detected by physical examination, previously treated or current treated ascites. Asymptomatic patients with only small amount of ascites on imaging were eligible; 2. patients with moderate pleural effusion, or a large amount of pleural effusion on one side, or caused respiratory dysfunction requiring drainage; 3. Uncontrolled metabolic disorder or other nonmalignant organ or systemic disease or reaction secondary to cancer, leading to a high medical risk and/or uncertainty in survival evaluation; 4. Known active pulmonary tuberculosis; 5. Interstitial lung disease requiring steroid hormone therapy; 6. Patients with significant malnutrition; 7. Known hypersensitivity to the study drug; 8. Patients with a history of psychotropic drug abuse and unable to quit or with mental disorders; 9. Female patients who are pregnant or lactating; 10. Participation in other clinical trials within four weeks; 11. History of other primary malignancies, except for the following: 1) malignancies that were in complete remission for at least 2 years prior to enrollment and did not require other treatment during the study period; 2) non-melanoma skin cancer or lentigo maligna with no sign of recurrence after adequate treatment; 3) carcinoma in situ with no sign of recurrence after adequate treatment; 12. Patients with concomitant diseases that seriously jeopardize the patient's safety or prevent patients from completion the study according to the investigator's judgment.   **Withdrawal Criteria**   1. Use of other anti-tumor therapies (including chemotherapy, targeted therapy or biological agents, etc.) that may affect the evaluation of efficacy during the study; 2. Patients who experienced serious adverse events and longer suitable for continue participation in the study according to the investigators’ judgment, or have unintended pregnancy; 3. Patients who are unwilling to continue the clinical trial and insist on withdrawing; 4. The investigator considered termination of the study necessary.   **Removal criteria**   1. Wrong dose and method of administration; 2. Patients who have received chemotherapy or drugs beyond the protocol during the trial; 3. Those who do not meet the criteria but included by mistake; 4. Patients without medication;   Note: Patients who met criteria 1-3 are included in the safety analysis.  **Discontinuation Criteria**   1. Patients with PD; 2. The researchers found severe safety problems based on the decisions of investigators; 3. Drug withdrawal for more than one cycle; 4. Dose reduction for more than 2 times; 5. Surgery is feasible after treatment and patients voluntarily undergo surgery.   Note: In the first 6 cycles of treatment, if one of the chemotherapy drugs has been dose reduced for more than twice or discontinued for more than one cycle, while the other chemotherapyc drugs can be used alone (e.g., only platinum is intolerant, while capecitabine, paclitaxel or gemcitabine are still tolerable), the latter and anlotinib can be retained; if the retained chemotherapyc drugs cannot be used alone, e.g., platinum, the patients should be withdrawn from the study. After 6 cycles, anlotinib maintenance could be continued if capecitabine was intolerable while anlotinib is tolerable. | | |
| Duration of trail | | Estimated start time: August, 2021  Estimated completion time of enrollment: August, 2022  Estimated completion time of this trail: August, 2023 |

1.1 Flow Chart of Clinical Trial

| Table 1. Clinical Trial Schedule | | | | | | | | | | | | |
| --- | --- | --- | --- | --- | --- | --- | --- | --- | --- | --- | --- | --- |
| Visit/Program ^B^ | Screening Period ^A^ | | Treatment period | | | | | | | End of Treatment/End of Study | | |
|  | Baseline Day | | Cycles 1-6 | | | | | | Maintenance therapy Period | **Out of group visit** ^[26]^ | **30-day Safety Follow-up** ^[27 ]^ | Survival Follow-up |
|  | - 28 to -1 | - 7 to -1 |  |  |  |  |  |  |  |  |  |  |
| Window period ^[1]^ | NA | NA | ± 3 | | | | | | ± 3 | ± 3 | ± 7 | ± 7 |
| Cycle |  |  | C 1D21 | C 2D21 | C 3D21 | C 4D21 | C 5D21 | C 6D21 | D21 every 3 cycles |  |  |  |
| Informed Consent ^[2]^ | × |  |  |  |  |  |  |  |  |  |  |  |
| Tumor history, other medical history, previous treatment history ^[3]^ | × |  |  |  |  |  |  |  |  |  |  |  |
| Radiographic evidence ^[4]^ | × |  |  |  |  |  |  |  |  |  |  |  |
| Inclusion and exclusion criteria check ^[5]^ | × |  |  |  |  |  |  |  |  |  |  |  |
| Pregnancy test ^[6]^ |  | × |  |  |  |  |  |  |  |  |  |  |
| Hepatitis B panel/Hepatitis C/HIV test ^[7]^ | × |  |  |  |  |  |  |  |  |  |  |  |
| Vital Signs and Physical Examination ^[8]^ |  | × | × | × | × | × | × | × | × | × | × |  |
| ECOG PS score |  | × | × | × | × | × | × | × | × | × | × |  |
| Blood pressure ^[9]^ |  | × |  | × |  | × |  | × | × | × | × |  |
| Coagulation function ^[10]^ |  | × | × | × | × | × | × | × | × | × | × |  |
| Blood routine ^[11]^ |  | × | × | × | × | × | × | × | × | × | × |  |
| Blood biochemistry ^[12]^ |  | × | × | × | × | × | × | × | × | × | × |  |
| Urine routine ^[13]^ |  | × |  | × |  | × |  | × | × | × | × |  |
| Stool routine ^[14]^ |  | × |  | × |  | × |  | × | × | × |  |  |
| Thyroid function ^[15]^ |  | × |  | × |  | × |  | × | × | × | × |  |
| Tumor markers ^[16]^ |  | × |  | × |  | × |  | × | × | × |  |  |
| Microcirculatory changes (not necessary) ^[ 17]^ |  | × |  | × |  | × |  | × | × | × |  |  |
| Electrocardiogram ^[18]^ |  | × | × | × | × | × | × | × | × | × | × |  |
| Echocardiogram ^[19]^ |  | × |  |  |  |  |  |  |  |  |  |  |
| Tumor imaging examination and evaluation ^[20]^ |  | × |  | × |  | × |  | × | × | × |  |  |
| Resectability Assessment of Liver Metastases ^[21]^ |  |  |  | × |  | × |  | × |  |  |  |  |
| Study drug ^[22]^ | | | | | | | | | | | | |
| Oxaliplatin |  |  | × | | | | | |  |  |  |  |
| Epilepsy |  |  | × | | | | | |  |  |  |  |
| Paclitaxel or Taxotere |  |  | × | | | | | |  |  |  |  |
| Capecitabine |  |  | × | | | | | | |  |  |  |
| Anlotinib |  |  | × | | | | | | |  |  |  |
| Documentation of concomitant medications ^[23]^ | × |  | × | | | | | | | × | × |  |
| Recording Adverse Events ^[24]^ | × |  | × | | | | | | | × | × |  |
| Survival Status ^[25]^ |  |  |  | | | | | | |  |  | × |

Note: In addition to the examination items and time points in the table, the investigator may add visits and other examination items as needed, and the examination results should be filled in the corresponding part of case report form (eCRF).

1. Informed consent at screening, Tumor history, other medical history, previous treatment history, hepatitis B 5/C/HIV tests, inclusion and exclusion criteria check, imaging evidence can be completed within 28 days prior to the first use of study drugs; Pregnancy test, coagulation function, hematology, blood biochemistry, urinalysis, stool routine, thyroid function, tumor markers, ECG, echocardiography and tumor imaging can be completed within 7 days prior to the first dose of study drugs; the examination results obtained before signing the informed consent form can be used for screening assessment if the requirements for this time are met.
2. All the examinations in other hospitals should be performed only under the special circumstances (e.g., the patients cannot come to our hospital for examination due to epidemic situation) and with the consent of the investigator. The examination results in other hospitals should be reported to the investigator in a timely manner, and the corresponding section in the case report form (or eCRF) should be filled in with the reasons.

[1]. Window: ± 7 days for imaging, 30-day safety follow-up and survival follow-up, and ± 3 days for other examinations.

[2]. Informed Consent: Performed within 28 days prior to enrollment. If the informed consent is revised during the study, the patient will be asked to do so if the investigator deems it necessary Second Signature.

[3]. Tumor history and other medical history: pathological results; tumor surgery, chemotherapy, radiotherapy and other diseases and treatment history, which should be performed within 28 days prior to enrollment.

[4]. Radiographic evidence: For patients with postoperative recurrence, radiographic evidence of tumor recurrence was collected and completed within 28 days prior to enrollment.

[5]. Inclusion and exclusion criteria check: Performed within 28 days prior to enrollment. Where The time of HER2-negative test report for gastric cancer should be completed within 3 months before enrollment, and HER2 testing should be repeated if it exceeds 3 months, Only H ER2 Only patients with negative test results; there is no time requirement for HER2-positive test reports, and patients previously confirmed to be HER2-positive are excluded; Immunohistochemical staining (IHC) Yes to screen for gastric cancer HER2 The preferred method of status. If the test result is IHC3 +, it can be directly judged as HER2 Positive; if test is IHC 1 + or IHC0, it is judged as negative; if the test result is IHC2 +, it must be used Fluorescence in situ hybridization (FISH) Test, H can only be judged when the FISH test result is positive ER2 Positive, negative by FISH is judged as H ER2 Negative.

[6]. Pregnancy testing was limited to women of childbearing age and was completed within 7 days prior to enrollment. Urine or serum pregnancy tests will be performed during treatment and at safety follow-up. If urine pregnancy test is positive, serum pregnancy test (performed at each study site or designated laboratory) must be performed, and the result of serum pregnancy should prevail.

[7]. Hepatitis B (5 items)/Hepatitis C/ HIV Test: Hepatitis B Five items (If Hepatitis B surface antigen (HBsAg) Positive HBV DNA quantitative detection is performed ), Hepatitis C antibody Inspection (Fig. If HCV antibody If positive, proceed HCV RNA quantification Test ), HIV testing , To be completed within 28 days prior to enrollment.

[8]. Vital signs examination: body temperature, respiratory rate, heart rate and body weight. Physical examination: examination of major body systems (general condition, mucocutaneous, head, neck, chest, abdomen, spine/extremities, other), completed within 7 days prior to enrollment. Every cycle during first 6 cycles of treatment; every 3 cycles during maintenance after 6 cycles (C9D21C12D21C15D21...) The examination should be performed once at the out-group visit and 30-day safety follow-up visit.

[9]. Blood pressure monitoring: Blood pressure monitoring was completed and recorded by the patients themselves, at least 3 times a week. If blood pressure was abnormal, testing was followed up and recorded every day. Blood pressure was measured by the investigator at each visit. Attention should be paid to the measurement of blood pressure. Smoking and drinking coffee are prohibited within 30 minutes before measurement. The patient has a quiet rest for at least 10 minutes. During measurement, the patient is placed in a sitting position with the elbow at the same level as the heart. The same side is used for each blood pressure measurement.

[10]. Coagulation function: Includes prothrombin time (PT), activated partial thromboplastin time (APTT), thrombin time (TT), fibrinogen (FIB), international normalized ratio (INR), Examined within 7 days prior to enrollment. The first 6 cycles during treatment, Every cycle; every 3 cycles during maintenance after 6 cycles (C9D21C12D21C15D21...) Check once Examinations are required at the check-out visit and at the 30-day safety follow-up visit.

[11]. Blood routine: Red blood cell count (RBC), hemoglobin (Hb), platelet count (PLT), white blood cell count (WBC), neutrophil count (ANC), lymphocyte count, examined within 7 days before enrollment. The first 6 cycles during treatment, Every cycle; every 3 cycles during maintenance after 6 cycles (C9D21C12D21C15D21...) Check once Examinations are required at the check-out visit and at the 30-day safety follow-up visit. If neutrophils ≤ 1 × 10 ^9^ /L or platelets ≤ 50 × 10 ^9^ /L, then the frequency of reexamination should be increased (once every 2 ~ 3 days).

[12]. Blood chemistry: Alanine aminotransferase (ALT), aspartate aminotransferase (AST), glutamyl transpeptidase (GGT), total bilirubin (TBIL), direct bilirubin (DBIL), alkaline phosphatase (ALP), blood urea nitrogen (BUN), total protein (TP), albumin (ALB), creatinine (Cr), blood glucose (GLU), Cholesterol (GHO), triglycerides (TG), Low Density Lipoproteins (LDL ), high-density lipoprotein (HDL ) , K +, Na +, Ca2 +, Cl- , examined within 7 days before enrollment. The first 6 cycles during treatment, Every cycle; every 3 cycles during maintenance after 6 cycles (C9D21C12D21C15D21...) Check once Examinations are required at the check-out visit and at the 30-day safety follow-up visit. If ALT or AST increases by 2 times compared with abnormal baseline values during the trial, the frequency of examination should be increased (1-2 times/week is recommended).

[13]. Urine routine: urine protein, urine glucose, Urinary occult blood Urine red blood cells, urine white blood cells, urine pH and urine ketone body. If urine protein ≥ by semi-quantitative method 2 + (e.g., urine dipstick) for 24-hour urine protein quantification, Examined within 7 days prior to enrollment. Every even cycle (C2D21C4D21C6D21) during first 6 cycles; every 3 cycles during maintenance after 6 cycles (C9D21C12D21C15D21...) Once, at the check-out visit, and at the 30-day safety follow-up visit.

[14]. Stool routine: fecal red blood cell, fecal white blood cell, occult blood test, Examined within 7 days prior to enrollment. During the first 6 cycles of treatment Every even cycle (C2D21C4D21C6D21); every 3 cycles during maintenance after 6 cycles (C9D21C12D21C15D21...) Exam once, required at check-out visit. It is performed according to the actual situation of the patient and does not have to be examined; if the patient cannot complete the examination due to constipation and other factors, this examination may not be performed.

[15]. Thyroid function: triiodothyronine (T3), thyroxine (T4), free triiodothyronine (FT3), free thyroxine (FT4), thyroid-stimulating hormone (TSH), examined within 7 days prior to enrollment. Every even cycle (C2D21C4D21C6D21) during first 6 cycles of treatment; every 3 cycles during maintenance after 6 cycles (C9D21C12D21C15D21...) Inspection 1 at check-out visit, and at 30-day safety follow-up visit.

[16]. Tumor markers: examined within 7 days prior to enrollment. For the first 6 cycles during treatment, every Even number Periodic examination (C2D21C4D21C6D21); every 3 cycles during maintenance therapy after 6 cycles (C9D21C12D21C15D21...) Exam once, required at check-out visit. Serum test is required for patients with colorectal cancer C A199, CA1 25. CA 724 and Fig. C EA; serum should be tested in patients with esophageal squamous cell carcinoma SCC, cytokeratin; Serum C should be measured in patients with gallbladder cancer A199, CA 125 and C EA; Serum C should be measured in patients with gastric cancer EA, A FP, C A125, CA199, C A724; serum for pancreatic cancer C EA, C A125, CA199.

[ 17]. Microcirculatory changes (leading site): including CT perfusion imaging technique, vascular endothelial function, and sublingual microcirculation measurement. Pre-enrollment Check within 7 days. For the first 6 cycles during treatment, every Even number Periodic examination (C2D21C4D21C6D21); every 3 cycles during maintenance therapy after 6 cycles (C9D21C12D21C15D21...) Exam once, required at check-out visit. This test is only performed in the leading site and is a non-clinical routine test item, so it is a non-essential item.

[18]. Electrocardiogram: Special attention was paid to QTc interval, which was examined within 7 days before enrollment. Every cycle during first 6 cycles of treatment; every 3 cycles during maintenance after 6 cycles (C9D21C12D21C15D21...) Inspection 1 at check-out visit, and at 30-day safety follow-up visit. ECG abnormalities should be confirmed twice (at an interval of 5 minutes, the QTc interval should be indicated).

[19]. Echocardiogram: within 7 days prior to enrollment, and is required to be supplemented only if clinically significant ECG abnormalities occur during treatment.

[20]. Tumor imaging examination: At baseline, subjects with esophageal squamous cell carcinoma must receive chest and abdominal CT or MRI examination, subjects with colorectal cancer, gastric cancer, biliary tract cancer or pancreatic cancer must receive chest, abdomen and pelvic CT or MRI examination, and the remaining sites are determined by the investigator according to clinical needs or actual needs; during the trial, brain MRI is required when brain metastasis is suspected and confirmed (CT can be used instead when MRI is prohibited), and bone scan is only performed when there is clinical need. Follow Up Imaging examination conditions should be the same as the baseline (including examination method, scanning slice thickness, contrast agent, etc.).

✓ During the screening period, tumor assessment must be completed within 7 days prior to the first dose for subjects. The imaging results obtained before signing the informed consent form must meet RECIST 1.1 criteria and be agreed by the investigator before tumor assessment during the screening period.

Tumor imaging will be performed every 2 cycles during the first 6 cycles of the study treatment period (C2D21C4D21C6D21), and every 3 cycles during the maintenance therapy period after 6 cycles (C9D21C12D21C15D21...) An imaging examination should be performed, and timely examination should also be performed if new lesions are suspected; timely imaging examination should be performed when subjects appear out for any reason (± 7 days, Fig. If the previous examination time is not more than 2 weeks away from the termination of treatment, it is not necessary to make examination again at the time of group withdrawal). Unscheduled imaging may be performed when disease progression is suspected (e.g., symptomatic deterioration).

Preoperative imaging assessment, if liver metastases are assessed as resectable and the patient is willing to undergo surgery, an imaging examination is required within 7 days before surgery.

In addition to radiologically confirmed disease progression, subjects who discontinue study treatment for other reasons should also undergo imaging as frequently as possible per protocol until documented disease progression, initiation of new antineoplastic therapy, or death.

[21]. Assessment of resectability of liver metastases: The resectability of liver metastases was assessed at the end of Cycle 2, Cycle 4, and Cycle 6.

[22]. Study drug dosage and administration:

During the first 6 cycles of treatment:

- Cohort A: ① Anlotinib: 1 capsule orally once daily (1 2 mg), for 2 weeks, followed by 1 week off, and 3 weeks as a treatment cycle; ② Oxaliplatin: 130 mg/m ^2^ On the first day, intravenous drip > 2h, d1 in each cycle, 3 weeks as a treatment cycle; ③ Capecitabine: 850 mg/m ^2^ Calculated according to the body surface area of the patient at baseline, once in the morning and once in the evening, for 2 weeks, followed by 1 week of drug withdrawal, and 3 weeks as a treatment cycle.
- Cohort B: ① Anlotinib: 1 capsule orally once daily (1 2 mg), for 2 weeks, followed by 1 week off, and 3 weeks as a treatment cycle; ② Paclitaxel: 135 mg/m ^2^ Intravenous drip > 2h, d1 in each cycle, hormone pretreatment before treatment, 3 weeks for a treatment cycle or other: at 75 mg/m ^2^ Intravenous drip for 1 hour, d1 medication in each cycle, 3 weeks for a treatment cycle. ③: administer after paclitaxel, at 60-75 mg/m ^2^ Intravenous drip, every cycle d 1 Or divided into d1-d3 medication, 3 weeks for a treatment cycle.
- Cohort C: ① Anlotinib: 1 capsule orally once daily (1 2 mg), for 2 weeks, followed by 1 week off, and 3 weeks as a treatment cycle; ② chemotherapeutic drugs and their usage and dosage are standard first-line chemotherapeutic drugs decided by the investigator (3-week chemotherapy regimen is selected);

Maintenance after 6 cycles:

- Cohort A and Cohort B were dosed identically: ① Anlotinib: as above; ② Metronomic capecitabine chemotherapy: 500 mg, twice daily (once in the morning and once in the evening), orally, daily;
- Cohort C: ① Anlotinib: the same as above; ② Preferred capecitabine for maintenance chemotherapy: 500 mg twice daily (morning and evening) by mouth every day.

✓ If a cycle is delayed, the schedule for the next cycle should not be changed. Per cycle D 1. Before administration, the diary card of the previous cycle and untaken drugs, bottles, aluminum foil plate and other consumables should be returned, and the drugs of the next cycle should be distributed.

[23]. Recording concomitant medications: concomitant medications and treatments were recorded within 28 days prior to enrollment and during the study, including generic name and daily dose of the drug; reason for drug treatment; start date and end date of the drug. Once a subject has discontinued trial treatment, only concomitant medications and treatments used for new or unresolved AEs related to trial treatment should be recorded.

[24]. Recording of adverse events: Recording of adverse events starts from signing of informed consent form to the end of safety follow-up.

[25]. Survival status: Survival follow-up will be performed once every 6 months by clinic visit or telephone visit. Survival status and subsequent anti-tumor treatment will be recorded. Death date will be collected if the subject dies.

[26]. Ex-group visit: The time of out-group visit is when the subject receives the study drug treatment; if the time between the examination at the previous visit and the termination of treatment is not more than 2 weeks, the re-examination is not required at the time of out-group visit. If a patient starts a new anticancer therapy within 30 days after the last treatment, then this safety follow-up visit should be performed within a 30-day window before initiation of the new anticancer therapy. If a patient is unable to return to the study center before starting a new treatment, the study center may collect any new safety information that appears during the end of treatment visit and the start of the new treatment by calling the follow-up phone.

[27]. The 30-day Safety Follow-up visit starts from the subject's last dose of study drug (Anlotinib) and only collects AEs that are possibly and definitely related to study drug.

1.2 Study Design Planning Diagram


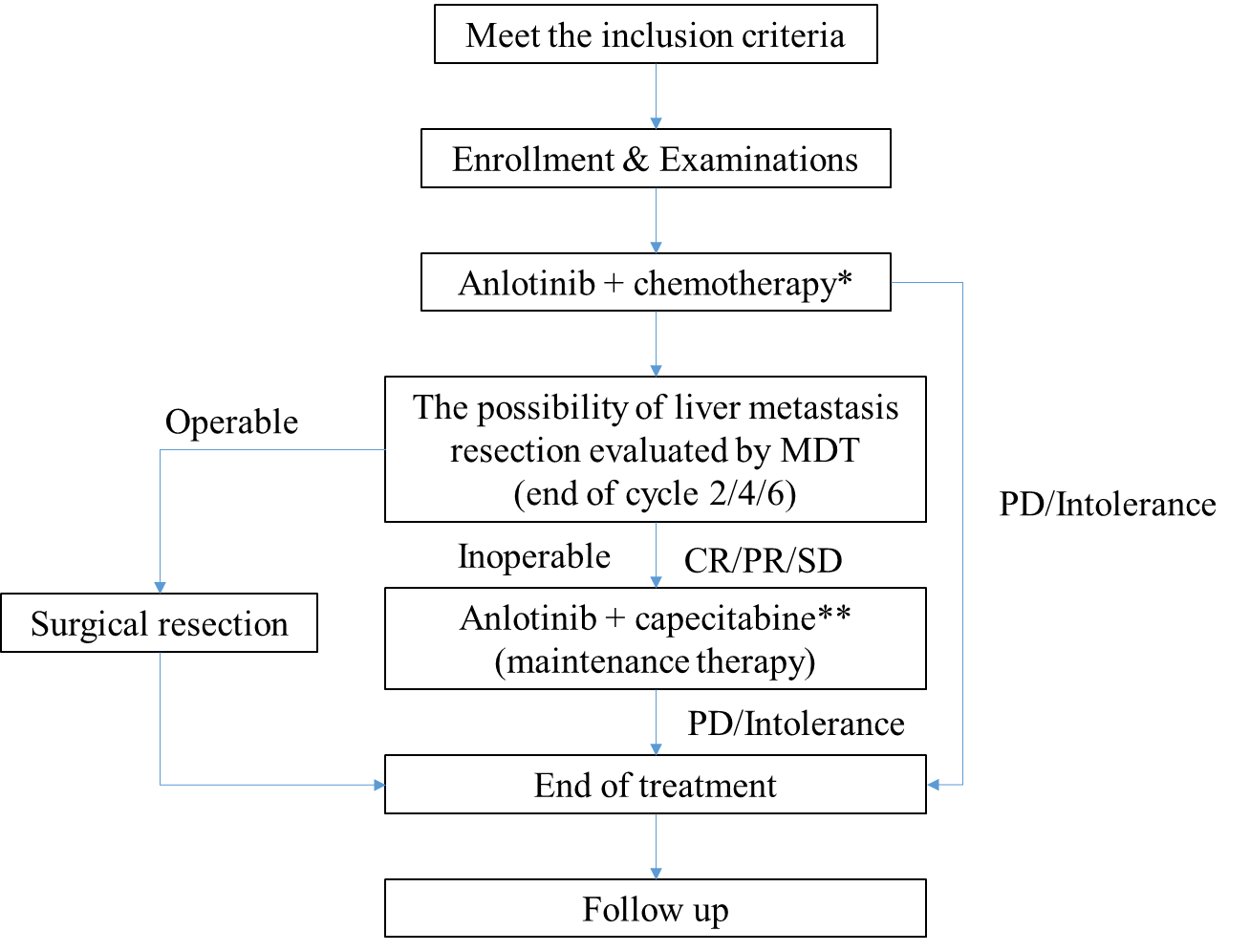


Figure 1: Study Design Planning Diagram

* Chemotherapy regimen for initial therapy (first 6 cycles): CAPEOX regimen for Cohort A; TP/DP regimen for Cohort B; and standard first-line chemotherapy regimen at the investigator's discretion for Cohort C (3-week treatment cycle)

** In the maintenance treatment, the chemotherapeutic agents in cohorts 1 and 2 are low-dose capecitabine; the chemotherapeutic agents in Cohort C are preferably low-dose capecitabine.

2 Study background

2.1 Study Feasibility

Gastrointestinal cancer represents a malignant neoplasm characterized by high morbidity and mortality rates. The incidence and mortality of prevalent cancers, including esophageal, liver, stomach, colorectal, and pancreatic cancers, rank among the top ten malignancies globally. According to the 2020 statistics from the International Agency for Research on Cancer (GLOBOCAN), approximately 5.09 million new cases of gastrointestinal cancer were reported (accounting for about 26.4% of all new cancer diagnoses) alongside roughly 3.61 million deaths (constituting around 36.3% of total cancer fatalities) worldwide [8]. In China specifically, esophageal cancer, liver cancer, stomach cancer, colorectal cancer, and pancreatic cancer accounted for 38% (1.497 million out of 3.929 million) of all newly diagnosed cases and represented 46% (1.077 million out of 2.338 million) of all cancer-related deaths in 2015 [9]. Gastrointestinal cancers lead both in incidence and mortality among malignant tumors within China.

The insidious onset of gastrointestinal cancers often results in subtle clinical manifestations coupled with a low rate of early diagnosis; consequently, most patients are identified at advanced stages frequently accompanied by distant metastasis—primarily affecting the liver as the most common organ involved via hematogenous spread. The prevalence of liver metastasis varies significantly across different types of gastrointestinal malignancies; notably, it is particularly pronounced in colorectal carcinoma where approximately 15-25% present with hepatic involvement at diagnosis while another similar percentage may develop such metastases post-radical resection [10]. Liver metastasis occurs in about 15.6% of esophageal carcinoma patients; furthermore, nearly half those diagnosed with stage IV esophageal carcinoma exhibit hepatic involvement [11]. For gastric carcinoma patients, the incidence ranges between approximately 9.9-18.7% [12]. Additionally, both pancreatic adenocarcinoma and biliary tract carcinomas predominantly involve the liver as their metastatic site—with around 65-80% exhibiting hepatic metastases at advanced stages [13-14]. The prognosis for gastrointestinal oncology patients presenting with liver metastases remains poor—especially those deemed unresectable—wherein less than a mere five percent achieve survival beyond five years following diagnosis related to colorectal carcinomas involving hepatic spread; conversely however radical resection can elevate this figure to between thirty to seventy percent [10]. Gastric carcinoma patients facing similar challenges have dismal outcomes as well—with five-year survival rates below ten percent[14-15], although surgical intervention post-resection may improve these figures to twenty-five to forty-two percent[15-17]. The absence of sensitive diagnostic markers contributes significantly towards an early detection rate under five percent for pancreatic neoplasms which complicates treatment options leading to exceedingly poor prognoses—a mere eight percent overall survival rate exists while that drops further downwards near one point eight percent when distant metastatic disease is present; yet again radical resections can yield upsurges reaching around twenty-five percent [19-20]. Unfortunately, too many biliary tract malignancy cases remain undiagnosed until late-stage progression has occurred resulting oftentimes into lost opportunities for curative surgery especially gallbladder or intrahepatic/extrahepatic variants severely impacting patient survivability benefits derived from timely interventions [13]. Current evidence substantiates that comprehensive therapeutic approaches enhance surgical candidacy concerning malignant hepatic lesions thereby alleviating tumor burdens effectively through multidisciplinary collaboration ultimately fostering improved long-term outcomes amongst affected individuals [10][12].

The vast majority of patients with liver metastases are unable to undergo radical resection, necessitating comprehensive treatment strategies that include systemic drug therapy aimed at controlling tumor burden, alleviating symptoms, prolonging survival, and enhancing the quality of life. Numerous clinical studies on advanced colorectal cancer have demonstrated that the overall response rate (ORR) for first-line treatments combining bevacizumab or cetuximab with chemotherapy can reach approximately 60%. Furthermore, the resection rate for liver metastases is significantly improved compared to chemotherapy alone, resulting in an overall survival (OS) duration of up to 32 months [21-24]. Systematic treatment approaches for advanced esophageal and gastric cancers also yield substantial anti-tumor efficacy; notably, advancements in immunotherapy have elevated the ORR for first-line esophageal cancer treatment from 29% to 45%, while gastric cancer has seen an increase from about 45% to around 60%. Patients with gastric cancer exhibiting liver metastasis experience considerable survival benefits [25-27]. In conclusion, employing combination therapies in patients suffering from colorectal cancer, gastric cancer, and esophageal cancer with liver metastasis enhances patient ORR, effectively manages disease progression, potentially improves the resectability of liver metastases and offers long-term survival advantages. Similarly, other gastrointestinal malignancies predominantly rely on systemic drug treatments. For instance, first-line therapy for advanced biliary tract tumors utilizing gemcitabine combined with cisplatin has extended OS from 6.6 months to 8.7 months [28], while treating advanced pancreatic cancer with a regimen of gemcitabine plus albumin-bound paclitaxel has increased patient ORR from 7% to 23%, improving OS similarly from 6.6 months to 8.7 months [29]. Despite these advancements in systemic treatments for pancreatic and biliary tract cancers providing some benefit to patients, their OS remains limited and does not fully address therapeutic needs. Given the large population affected by gastrointestinal cancers, there exists a pressing demand for alternative or more effective treatment options. Therefore, it is imperative to explore safer and more efficacious therapeutic avenues for patients diagnosed with gastrointestinal cancers accompanied by liver metastasis.

2.2 Anlotinib Hydrochloride

2.2.1 Basic information and pharmacological mechanism of Anlotinib Hydrochloride

Anlotinib Hydrochloride is a multi-target tyrosine kinase inhibitor independently developed in China, which can effectively inhibit VEGFR1-3, PDGFRα/β, FGFR1-4, c-Kit and other kinases, and has anti-tumor angiogenesis and tumor growth inhibition effects. In particular, it shows highly selective inhibition of vascular endothelial growth factor receptor 2 (VEGFR2/KDR) and VEGFR3. Inhibitory concentration 50% (IC50) is less than 1.0 nM, with a pronounced anti-neo-angiogenic effect. Anlotinib hydrochloride is produced as hard capsules. The structural formula of the main component anlotinib is as follows:

Molecular formula: C_23_H_22_FN_3_O_3_·2HCl

Molecular weight: 480.36

2.2.2 Preclinical Studies of Anlotinib Hydrochloride

Preclinical pharmacodynamic studies have shown that anlotinib hydrochloride can significantly inhibit the growth of a variety of human tumor xenografts in nude mice, and cause tumor shrinkage in some ovarian and lung cancer tumor-bearing mice, and anlotinib hydrochloride is well tolerated by tumor-bearing mice. No associated toxicity was observed in toxicological studies of acute toxicity, long-term toxicity, mutagenicity, and teratogenicity in animals.

Animal pharmacokinetic studies of anlotinib hydrochloride were performed in rats and dogs. 1) Tissue distribution of tumor-bearing mice: The highest concentrations were observed in the lung and liver (about 10-14 times of plasma AUC), followed by the kidney (about 5.9-8.6 times of plasma AUC), the tumor AUC was about 2.4-2.6 times of plasma AUC, and the concentration in the colon was similar to that in plasma (about 0.8-1.0 times of plasma AUC). The AUC of anlotinib concentration in each tissue was positively correlated with the dose administered. 2) Excretion: The cumulative excretion of anlotinib hydrochloride prototype drug in urine (0-72 h), feces (0-72 h) and bile (0-24 h) was less than 5% of the injected drug dose, suggesting that metabolic transformation is the main elimination route of anlotinib hydrochloride. 3) Metabolites: 23 metabolites were detected in bile, 16 metabolites in urine, 12 metabolites in feces and 8 metabolites in plasma. 4) Metabolic enzyme activity: anlotinib was found to have a strong inhibitory effect on human drug metabolism enzyme CYP3A4 (IC500.69μM) and CYP2C9 (IC50 1.23μM). In addition, anlotinib showed a certain inhibitory effect on CYP2C19 (IC50 1.3μM), but a weak inhibitory effect on CYP1A2 (IC50 7.4μM) and CYP2D6 (IC50 10.4μM).

2.2.3 Phase I clinical trials

The phase I clinical trial of anlotinib tolerability and human pharmacokinetics study showed that anlotinib had a long elimination half-life in human body (116±47h, range 80-100 h), and drug accumulation was observed in patients with continuous administration. There were more adverse reactions after taking 10mg anlotinib for 2 cycles (28 days as a treatment cycle). From the perspective of patient tolerance, we used continuous medication for two weeks and one week off. Patients tolerated 10 mg qd well with two weeks in a row and one week off, while 16 mg qd two weeks in a row and one week off showed serious adverse reactions, so it was reduced to 12mg qd two weeks in a row and one week off. Among the 21 patients, 5 patients had 7 grade 3 adverse reactions (increased triglyceride, hypertension, bilirubin, etc.), all of which were common and controllable adverse reactions. Therefore, the regimen of 12 mg qd with two weeks in a row and one week off is recommended for subsequent studies.

The pharmacokinetics study of anlotinib in healthy subjects showed that the peak time of anlotinib in human body was prolonged after high-fat diet, and the absorption of anlotinib was slightly reduced (about 80% of that of fasting). Therefore, it is recommended to take this drug in the fasting state during phase II clinical trials

Table 2: Summary of adverse reactions in phase I clinical trial

| Adverse reactions  N = 21 n (%) | Number of grade I/II AEs | | Number of grade III AEs | |
| --- | --- | --- | --- | --- |
|  | First 2 cycles | Study process | First 2 cycles | Study process |
| At least one occurrence | 21 (100%) | 21 (100%) | 2 (9.52%) | 7 (33.33%) |
| Hand-foot skin reaction | 4 (19.05%) | 10 (47.62%) | 0 | 1 (4.76%) |
| Rash | 4 (19.05%) | 6 (28.57%) | 0 | 0 |
| Blood pressure increased | 5 (23.81%) | 5 (23.81%) | 0 | 2 (9.52%) |
| Proteinuria | 5 (23.81%) | 14 (67%) | 0 | 0 |
| Triglyceride increased | 6 (28.57%) | 11 (52.38%) | 1 (4.76%) | 2 (9.52%) |
| Total cholesterol increased | 6 (28.57%) | 13 (62%) | 0 | 0 |
| Low density lipoprotein increased | 4 (19.05%) | 11 (52.38%) | 0 | 0 |
| A low | 8 (38.10%) | 12 (57%) | 0 | 0 |
| Hyperthyroidism | 2 (9.52%) | 2 (9.52%) | 0 | 0 |
| Thyrotropin increased | 2 (9.52%) | 4 (19.05%) | 0 | 0 |
| ALT increased | 6 (28.57%) | 10 (47.62%) | 0 | 0 |
| AST increased | 4 (19.05%) | 9 (42.86%) | 0 | 0 |
| Creatinine increased | 1 (4.76%) | 2 (9.52%) | 0 | 0 |
| Total bilirubin increased | 5 (23.81%) | 8 (38.10%) | 0 | 0 |
| Direct bilirubin increased | 3 (14.29%) | 8 (38.10%) | 0 | 1 (4.76%) |
| Indirect bilirubin increased | 4 (19.05%) | 5 (23.81%) | 0 | 0 |
| Lipase | 1 (4.76%) | 5 (23.81%) | 1 (4.76%) | 1 (4.76%) |
| Blood amylase | 4 (19.05%) | 9 (42.86%) | 0 | 0 |
| Cardiac enzymes abnormal | 2 (9.52%) | 3 (14.29%) | 0 | 0 |
| White blood cell decreased | 3 (14.29%) | 6 (28.57%) | 0 | 0 |
| Neutrophils decreased | 0 | 2 (9.52%) | 0 | 0 |
| Platelet decreased | 0 | 2 (9.52%) | 0 | 0 |
| Hemorrhage | 0 | 1 (4.76%) | 0 | 0 |
| Urinary occult blood | 5 (23.81%) | 8 (38.10%) | 0 | 0 |
| Asthenia | 5 (23.81%) | 7 (33.33%) | 0 | 0 |
| Diarrhea | 6 (28.57%) | 7 (33.33%) | 0 | 0 |
| Hoarseness | 3 (14.29%) | 5 (23.81%) | 0 | 0 |
| Nausea | 3 (14.29%) | 3 (14.29%) | 0 | 0 |
| Decreased appetite | 1 (4.76%) | 2 (9.52%) | 0 | 0 |
| Toothache | 1 (4.76%) | 4 (19.05%) | 0 | 0 |
| Gingivitis | 1 (4.76%) | 1 (4.76%) | 0 | 0 |
| Pain | 4 (19.05%) | 4 (19.05%) | 0 | 0 |
| Sore throat | 1 (4.76%) | 4 (19.05%) | 0 | 0 |
| Dizziness/headache | 1 (4.76%) | 2 (9.52%) | 0 | 0 |
| Fever | 1 (4.76%) | 2 (9.52%) | 0 | 0 |
| Oral mucositis | 0 | 2 (9.52%) | 0 | 0 |
| Tinnitus | 1 (4.76%) | 1 (4.76%) | 0 | 0 |
| Premature beat | 0 | 1 (4.76%) | 0 | 0 |

2.3 Feasibility of Anlotinib Hydrochloride in the Treatment of Gastrointestinal Tumors

Currently, anlotinib has been approved for the treatment of advanced non-small cell lung cancer, small cell lung cancer, soft tissue sarcoma, and medullary thyroid carcinoma. In addition, anlotinib is also being investigated in gastrointestinal malignancies, including esophageal squamous cell carcinoma and colorectal cancer

2.3.1 Studies of anlotinib hydrochloride in patients with advanced colorectal cancer

2.3.1.1 A double-blind, randomized, placebo-controlled phase 3 study of anlotinib in third-line or more therapy for advanced colorectal cancer (ALTER-0703)

The previous single-arm, phase II clinical study of anlotinib as a single agent in the treatment of advanced colorectal cancer as third-line or above showed that ORR was 6.45%, mPFS was 5.62 months, and mOS was 9.33 months. Anlotinib showed preliminary efficacy in the treatment of advanced colorectal cancer as third-line or above. Subsequently, a double-blind, randomized, placebo-controlled phase III study (ALTER-0703) of anlotinib as a third-line or later treatment for advanced colorectal cancer was conducted. Baseline characteristics of patients in both groups were as follows: About 70% patients had liver metastasis, about 40% patients were KRAS wild-type, 80% patients had received 2-3 lines of systemic chemotherapy, about 30% patients had received VEGFR-targeted drug therapy, and about 90% patients had received surgical treatment. The results showed that [30] anlotinib was associated with a significant PFS benefit compared with placebo (4.14 months vs. 1.45 months; p<0.0001) (Figure. 2A), with a 66% reduction in the risk of disease progression, and a significant PFS benefit of anlotinib was observed in all subgroups, especially in patients with RAS wild-type and liver metastases. ORR was improved (4.26% vs. 0.73%; p=0.069), and DCR was significantly improved (75.9% vs. 30.7%; p < 0.0001). Across all populations, the median OS was improved, and OS subgroup analysis showed a significant OS benefit with anlotinib versus placebo in patients with RAS/BRAF wild-type colorectal cancer (11.0 months vs. 6.7 months; p=0.043) (Figure 2B). In terms of safety, the common adverse reactions in the anlotinib group were hypertension, hand-foot skin syndrome, proteinuria, diarrhea, fatigue, etc., most of which were grade 1-2. The common adverse reactions of grade 3 and above were hypertension (20.9%), hand-foot skin syndrome (6.38%), proteinuria (4.36%), diarrhea (3.9%), hypertriglyceridemia (3.9%), etc. No unexpected adverse reactions occurred, and the safety was manageable.

B

A


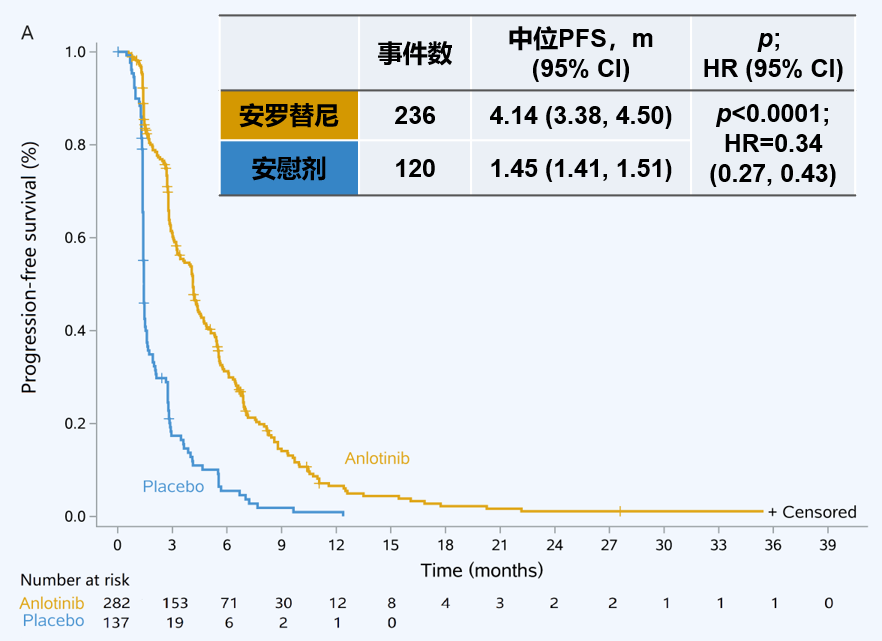

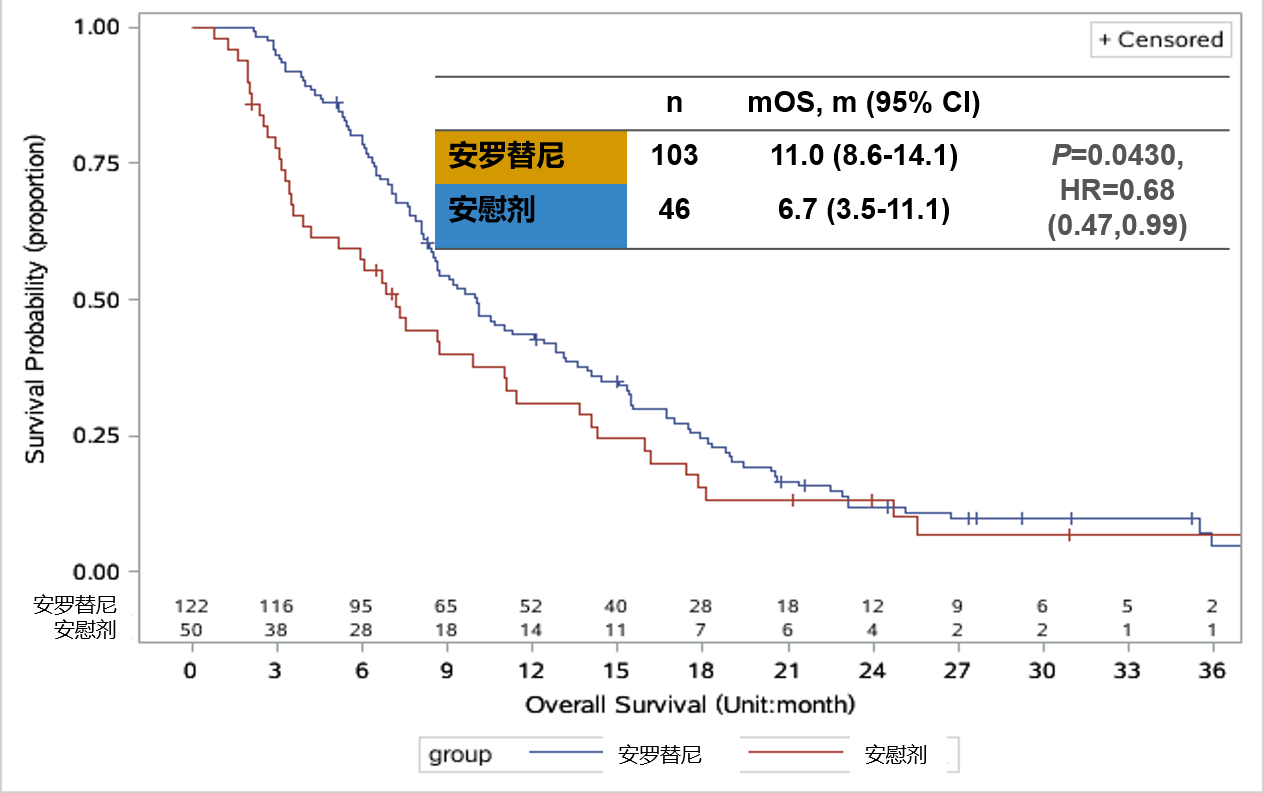


Figure 2. Anlotinib versus placebo in patients with advanced colorectal cancer treated with third-line or more therapy. A. Progression-free survival curve; B. RAS/BRAF wild-type colorectal cancer

2.3.1.2 Anlotinib in combination with capeox as first-line treatment for ras/braf wild-type advanced colorectal cancer: single-arm, multicenter exploratory clinical study (ALTER-C002)

The ALTER-C002 study [32] involved patients with unresectable, previously untreated RAS/BRAF wild-type metastatic colorectal cancer who received anlotinib (12 mg, p.o. qd, d1-14) and oxaliplatin (130mg/m2, ivgtt, D1-14) for the first six cycles of initial therapy. d1) and capecitabine (850mg/m2, p.o. bid, d1-d14) for 21 days as a cycle; Six cycles were followed by maintenance therapy, in which patients received anlotinib (12 mg, p.o. qd, d1-14) and capecitabine (850mg/m2, p O. bid, d1-d14) in a 21-day cycle until disease progression or intolerable toxicity. Up to September 2020, 19 patients had been enrolled, and 90% of them had liver metastasis. Twelve patients could be evaluated; the best response assessment was PR in 10 patients and SD in 2 patients (Figure. 4). The ORR was 83.3%, and the DCR was 100.0%. The common adverse reactions of grade 3 or above were hypertension (52.6%), diarrhea (15.8%), leukopenia/neutropenia (15.8%), etc. No grade 5 adverse reactions occurred.


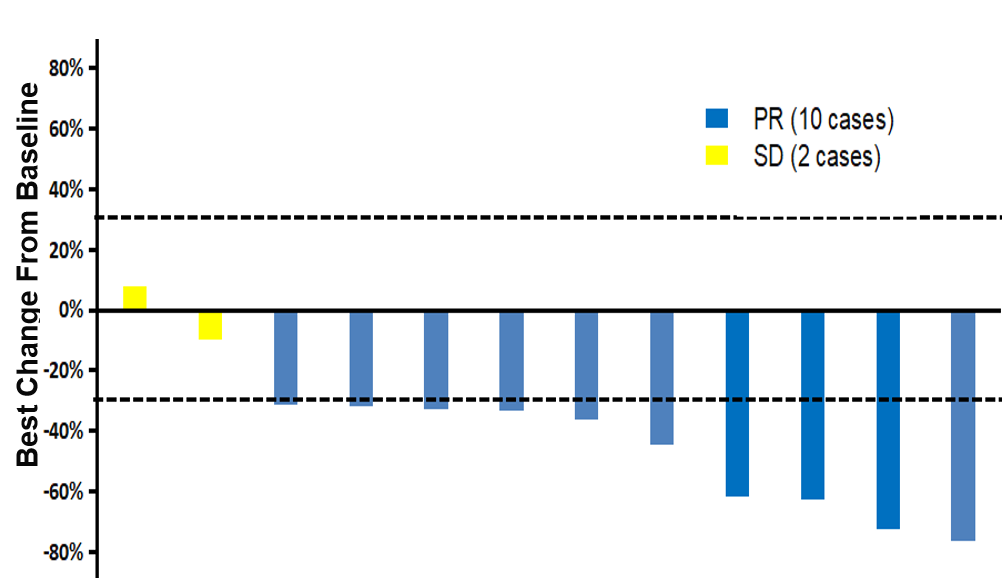


A

Figure 4. Tumor Response of anlotinib in combination with capeox in first-line treatment of ras/braf wild-type patients with advanced colorectal cancer

2.3.1.3 Single-arm, multicenter exploratory clinical study of anlotinib hydrochloride in combination with xelox regimen as first-line and maintenance therapy for metastatic colorectal cancer (ALTER-C001)

The C001 study [31] was designed to evaluate the efficacy and safety of anlotinib in combination with XELOX as first-line treatment and anlotinib as maintenance monotherapy in the treatment of metastatic colorectal cancer. For the first six cycles of initial therapys, subjects received anlotinib (10 mg, p.o. qd, d1-14) and oxaliplatin (130mg/m2, ivgtt, D1-14). d1) and capecitabine (1000mg/m2, p.o. bid, d1-d14) for 21 days as a cycle. During the maintenance therapy, patients received anlotinib monotherapy (12 mg, p.o. qd, d1-14) for 21 days as a cycle until disease progression or intolerable adverse events. As of September 2020, nine patients had been enrolled, and six patients could be evaluated, of which four patients were evaluated as PR on the first imaging (C2D21) (Figure 3A), one patient was evaluated as reduced SD, and one patient was evaluated as PD with significant reduction in target lesions but new lesions (Figure 3B). The ORR and DCR were 66.7% and 83.3%, respectively. In terms of safety, the high incidence of adverse reactions in all grades included leukopenia (55%) and diarrhea (55%). The adverse reactions were mainly grade 1-2, and the incidence of grade 3-4 adverse reactions was low (22% hypertriglyceridemia, 11% hypertension, 11% neutropenia, 11% lipase elevation), and no grade 5 adverse reactions.


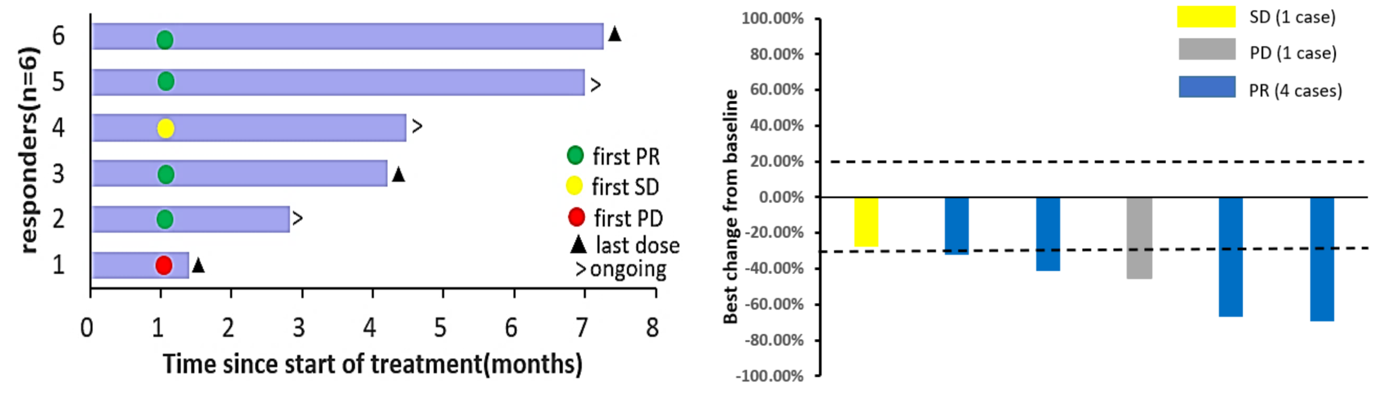


A

B

Figure 3. Anlotinib in combination with xelox regimen as first-line and maintenance therapy for metastatic colorectal cancer. A, B. Tumor response

2.3.2 Study of anlotinib in patients with advanced esophageal squamous cell carcinoma

2.3.2.1 Phase II double-blind, randomized, placebo-controlled study of anlotinib in second-line and above treatment of advanced esophageal squamous cell carcinoma (ALTER- 1102)

A total of 164 patients with stage IV esophageal squamous cell carcinoma who had received at least one prior failed platinum-based or taxane-containing chemotherapy were enrolled in the ALTER-1102 study [33] according to 2: 1 Randomized to anlotinib (109 patients) or placebo (55 patients), approximately 90% of patients had distant organ metastases, most had undergone tumor surgery, and approximately 64% had failed second-line or more chemotherapy. The results showed that anlotinib was associated with a significantly longer PFS benefit than placebo (3.02 vs. 1.41 months; p<0.0001) (Figure 4), and the risk of disease progression was reduced by 54%. ORR was improved (7.34% vs. 3.64%; p=0.4978), and DCR was significantly improved (64.22% vs. 18.18%; p < 0.0001). In terms of safety (Table 3), the incidence of all adverse reactions in the two groups was 94% and 82%, respectively, and the incidence of grade 3-4 adverse reactions was 37% and 11%, respectively. Common adverse events (> 20%) included hypertension, anorexia, hypothyroidism, hand-foot syndrome, proteinuria and elevated TSH. The most common grade 3 toxicities were hypertension (16%) and anorexia (6%). Except for hypertension, the other grade 3 and above adverse reactions did not increase significantly, and the safety was controllable. Based on the results of this study, the 2019 CSCO Guidelines for the Diagnosis and Treatment of Esophageal Cancer [20] has made anlotinib as a level II recommendation (level 2A) for second-line or later-line treatment of esophageal squamous cell carcinoma.


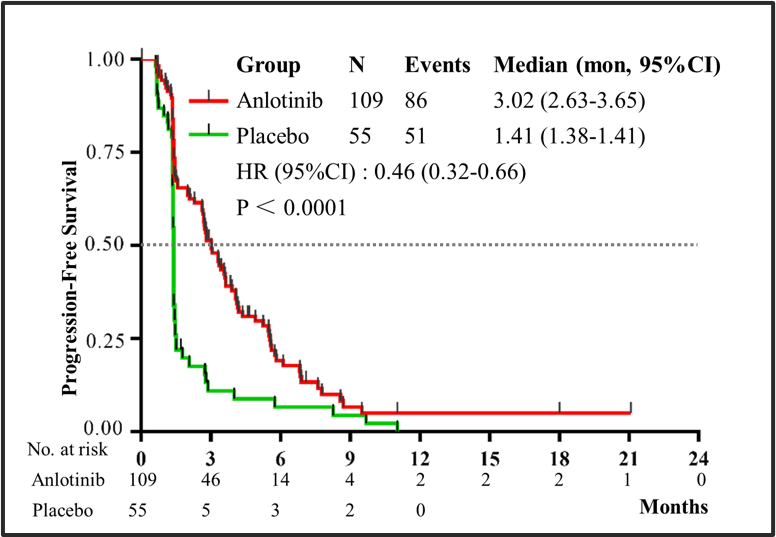


Figure 4. Progression-free survival curve and efficacy of anlotinibe and placebo in patients with advanced esophageal squamous cell carcinoma

Table 3: Adverse reactions of all grades (> 10%) and grade 3-5 (> 1%) occurring in patients with advanced esophageal squamous cell carcinoma treated with anlotinib and placebo as second-line and above therapy

|  | Anlotinib group (n = 109) | | | | Placebo (n = 55) | | |
| --- | --- | --- | --- | --- | --- | --- | --- |
| N (%) | All grades | Grade 3 | Grade 4 | All grades | | Grade 3 | Grade 4 |
| All adverse reactions | 102 (94%) | 37 (34%) | 3 (3%) | | 45 (82%) | 6 (11%) | 0 |
| Fatigue | 62 (57%) | 2 (2%) | 0 | | 19 (35%) | 1 (2%) | 0 |
| Hypertension | 59 (54%) | 17 (16%) | 0 | | 9 (16%) | 0 | 0 |
| Decreased appetite | 47 (43%) | 6 (6%) | 0 | | 14 (25%) | 2 (4%) | 0 |
| Hypothyroidism | 39 (36%) | 1 (< 1%) | 0 | | 3 (5%) | 0 | 0 |
| Palmar-plantar erythrodysesthesia syndrome | 29 (27%) | 2 (2%) | 0 | | 0 | 0 | 0 |
| Proteinuria | 28 (26%) | 1 (< 1%) | 0 | | 7 (13%) | 0 | 0 |
| TSH increased | 26 (24%) | 1 (< 1%) | 0 | | 1 (2%) | 0 | 0 |
| Weight decreased | 21 (19%) | 3 (3%) | 0 | | 3 (5%) | 0 | 0 |
| Diarrhea | 19 (17%) | 0 | 0 | | 4 (7%) | 0 | 0 |
| White blood cell count decreased | 18 (17%) | 0 | 0 | | 2 (4%) | 0 | 0 |
| AST increased | 18 (17%) | 2 (2%) | 0 | | 2 (4%) | 0 | 0 |
| QT prolongation | 17 (16%) | 2 (2%) | 0 | | 6 (11%) | 0 | 0 |
| Hypercholesterolemia | 16 (15%) | 0 | 0 | | 4 (7%) | 0 | 0 |
| Hypertriglyceridemia | 15 (14%) | 0 | 0 | | 4 (7%) | 0 | 0 |
| GGT increased | 15 (14%) | 3 (3%) | 0 | | 3 (5%) | 1 (2%) | 0 |
| ALT increased | 15 (14%) | 1 (< 1%) | 0 | | 6 (11%) | 0 | 0 |
| Dysphonia | 15 (14%) | 0 | 0 | | 5 (9%) | 0 | 0 |
| LDL increased | 14 (13%) | 1 (< 1%) | 0 | | 2 (4%) | 0 | 0 |
| Platelet count decreased | 14 (13%) | 2 (2%) | 0 | | 3 (5%) | 0 | 0 |
| Oropharyngeal pain | 13 (12%) | 0 | 1 (< 1%) | | 2 (4%) | 0 | 0 |
| Abdominal pain | 13 (12%) | 0 | 0 | | 1 (2%) | 0 | 0 |
| Red blood cell urine positive | 12 (11%) | 0 | 0 | | 2 (4%) | 0 | 0 |
| Neutrophil count decreased | 12 (11%) | 0 | 0 | | 1 (2%) | 1 (2%) | 0 |
| Rash | 11 (10%) | 0 | 0 | | 2 (4%) | 0 | 0 |
| Nausea | 11 (10%) | 0 | 0 | | 3 (5%) | 1 (2%) | 0 |
| Lymphocyte count decreased | 10 (9%) | 3 (3%) | 0 | | 3 (5%) | 0 | 0 |
| Bilirubin conjugated increased | 9 (8%) | 3 (3%) | 0 | | 1 (2%) | 0 | 0 |
| HYPONATREMIA | 8 (7%) | 4 (4%) | 0 | | 0 | 0 | 0 |
| Hyperglycaemia | 5 (5%) | 0 | 0 | | 6 (11%) | 0 | 0 |
| Hemoptysis | 5 (5%) | 2 (2%) | 0 | | 1 (2%) | 0 | 0 |
| Hypokalemia | 4 (4%) | 2 (2%) | 1 (< 1%) | | 0 | 0 | 0 |
| Pneumonitis | 3 (3%) | 2 (2%) | 0 | | 0 | 0 | 0 |
| Insomnia | 1 (< 1%) | 0 | 0 | | 1 (2%) | 1 (2%) | 0 |

**2.3.2.2 Single-arm, multicenter exploratory clinical study of paclitaxel and cisplatin combined with anlotinib as first-line treatment in advanced esophageal squamous cell carcinoma (ALTER-** **E002)**

The ALTER-E002 study [34] involved patients with previously untreated, unresectable/locally advanced recurrent or metastatic esophageal squamous cell carcinoma who received 4-6 cycles of initial therapy. Subjects received anlotinib (10 mg, p.o. qd, d1-14) plus paclitaxel (135mg/m2, i.v., d1) and cisplatin (60-75mg/m2, i.v., d1-3) for 21 days as a cycle; After 6 cycles, subjects received single-agent maintenance therapy with anlotinib (10 mg, p.o. qd, d1-14) as a 21-day cycle until disease progression. Up to August 2020, 27 patients had been enrolled and all could be evaluated. 70.40% of the patients were over 60 years old, 51.90% of the patients had ECOG PS greater than 1, 88.9% had distant metastasis, 88.90% of the patients were in stage Ⅳ b, and more than 50% of the patients had received previous surgical treatment. The results of the study showed that among 27 patients with esophageal squamous cell carcinoma who were evaluated for efficacy, 2 patients achieved complete response (CR), 18 patients achieved partial response (PR), and 7 patients achieved stable disease (SD). The RR was 74.1% (95%CI: 53.7%-88.9%), and the DCR was 100.0% (95%CI: 87.2%-100.0%) (FIG. 6-A); The PFS of 1 case was 2.30 months, which was contrary to the study protocol because of surgical treatment. One patient died due to tumor, and the PFS was 1.41 months. The remaining 25 patients were still receiving treatment, and mPFS and mOS were not reached. Preliminary survival data results based on the date of the last visit showed that the longest duration of treatment (DoT) for the current patient was 8.41 months (as of August 2020) (Figure 6-B). In terms of safety, the adverse reactions that occurred were mostly related to chemotherapy, such as bone marrow suppression (70.4%), gastrointestinal reactions (77.8%), diarrhea (63%), etc. Most of the adverse reactions were grade 1-2, and the adverse reactions of grade 3 and above were common such as bone marrow suppression (18.5%) and hypertension (7.4%).


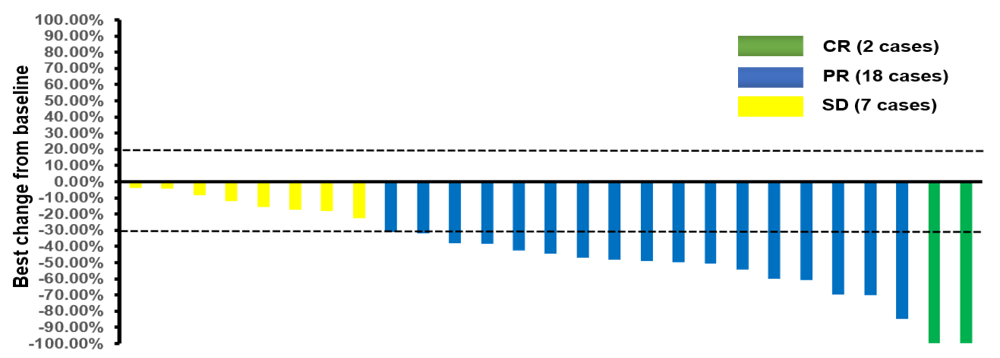


A


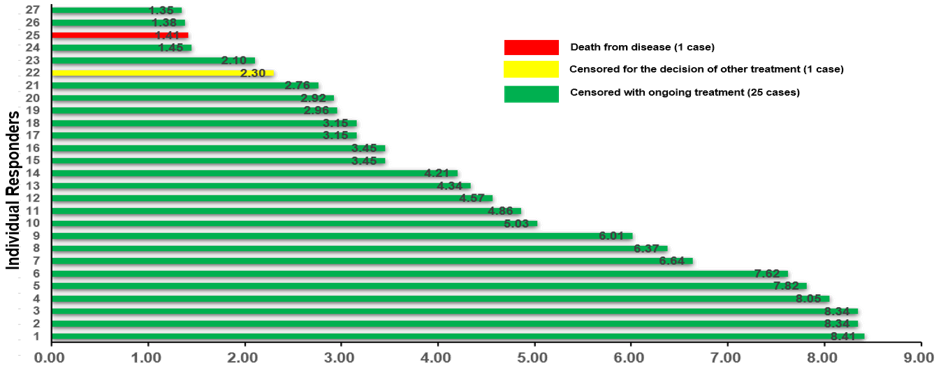


B

Figure 6. Paclitaxel and cisplatin combined with anlotinib hydrochloride capsules in first-line treatment of advanced esophageal squamous cell carcinoma. A. Tumor response; B. Subjects progression.

The above studies have shown that anlotinib has preliminary anti-tumor efficacy in the field of gastrointestinal cancer, and the adverse reactions are controllable. A case analysis of the existing anlotinib plus chemotherapy first-line treatment ALTER-E002, ALTER-C001, and ALTER-C002 studies found that patients with esophageal squamous and colorectal cancer with liver metastases (n=20) had an ORR of 75% and a DCR of 100%. In conclusion, anlotinib combined with chemotherapy has preliminary efficacy and tolerable safety in the treatment of colorectal cancer or esophageal phosphorous cancer with liver metastasis. However, the efficacy and safety of anlotinib in gastrointestinal tumors with liver metastasis need to be further verified to provide more treatment options for these patients. Therefore, the efficacy and safety of anlotinib hydrochloride combined with chemotherapy as first-line and maintenance therapy for gastrointestinal cancer patients with liver metastasis are worthy of further exploration.

3 Study objectives

To explore the efficacy and safety of anlotinib combined with chemotherapy in the first-line and maintenance therapy of gastrointestinal cancer patients with unresectable liver metastases

3.1 Primary Study Objective

- Objective response rate (ORR)

3.2 Secondary study objectives

- Progression-free survival (PFS);
- Disease control rate (DCR, CR + PR + SD);
- Duration of response (DoR);
- Overall survival (OS);
- Conversion rate of liver metastases;
- Safety
  1. Exploratory objective (leading site)
- To evaluate the changes of blood perfusion of tissues and organs in patients with gastrointestinal cancer and liver metastasis treated with anlotinib combined with chemotherapy as first-line and maintenance therapy by CT perfusion imaging. In addition, non-invasive techniques such as vascular endothelial function and sublingual microcirculation measurement were used to evaluate the microcirculation changes of patients during the study period

4 Study plan

4.1 Study design

A multi-cohort, multi-center trial. Each study site selected appropriate patients and conducted the clinical trial in a competitive manner.

4.2 Study Duration

Three weeks is a treatment cycle of the above drugs, and tumor efficacy is evaluated at the end of cycle 2, 4 and 6; if necessary, the MDT team is required to assess the possibility of surgical resection of liver metastases. If surgical resection is possible, anlotinib and chemotherapeutic drugs should be discontinued (4 weeks before surgery or determined by the investigator). In case of the liver metastases is unresectable after 6 cycles of treatment, patients with CR/PR/SD were switched to maintenance therapy with anlotinib (12 mg, po, qd, d1-d14, Q3W) combined with metronomic capecitabine chemotherapy (500 mg, po, bid, daily) until disease progression or intolerance.

4.3 Study Population

Patients with measurable liver metastases, advanced colorectal cancer, esophageal squamous cell carcinoma or other gastrointestinal tumors with unresectable liver metastases who are eligible. Patients must sign an informed consent prior to the clinical trial.

4.3.1 Inclusion criteria

Patients who meet all the following inclusion criteria can be included in this trial:

1. The patient voluntarily joined the study, signed the informed consent form, had good compliance and cooperated with the follow-up;
2. Male or female patients aged 18-75 years;
3. Cohort A: Patients histopathologically or cytologically confirmed colorectal cancer at stage IV (T_Any_N_Any_M1) with unresectable liver metastases;

Cohort B: Patients histopathologically or cytologically confirmed esophageal squamous cell carcinoma at stage IVb (T_Any_N_Any_M1) with liver unresectable metastases (excluding mixed adenosquamous carcinoma);

Cohort C: Patients histopathologically or cytologically confirmed other gastrointestinal tumors with liver metastases (excluding gastrointestinal stromal tumors, neuroendocrine tumors and other malignant tumors of non-glandular epithelial origin);

1. No previous systemic therapy, including chemotherapy, targeted therapy and immunotherapy;

Note: Patients who relapsed after receiving neoadjuvant (radio)chemotherapy plus radical surgery for more than 6 months, or relapsed after receiving adjuvant (radio)chemotherapy or radical concurrent chemoradiotherapy for more than 6 months were eligible;

1. At least one measurable lesion in the liver metastases according to the Response Evaluation Criteria in Solid Tumors (RECIST) Version 1.1, and the target lesions must contain liver metastases. The selected target lesions have not received local therapy such as radiotherapy or TACE within 6 months;

Note: Lesions in the area of previous local treatment can also be selected as target lesions if progression is confirmed and meets RECIST 1.1 criteria;

1. Palliative therapy for localized lesions (non-target lesions) should be completed for> 2 weeks;
2. ECOG PS score: 0 ~ 1;
3. Life expectancy of more than 3 months;
4. Adequate organ and bone marrow function, defined as meeting the following criteria (within 7 days prior to enrollment):
5. The blood routine examination criteria shall meet:

- Hemoglobin level (HB) ≥ 90 g/L (no blood transfusion within 14 days);
- Absolute neutrophil count (ANC) ≥ 1.5 × 10^9^ /L;
- Platelet count (PLT) ≥ 90 × 10^9^ /L.

1. Biohemical tests must meet the following criteria:

- Serum total bilirubin (TBIL) ≤ 1.5 × upper limit of normal (ULN);
- Alanine aminotransferase (ALT) and aspartate aminotransferase (AST) ≤ 5 ULN;
- Serum creatinine (Cr) ≤ 1.5 ULN or creatinine clearance (CCr) ≥ 60ml/min; (Cockcroft-Gault formula)

1. Adequate coagulation function defined as international normalized ratio (INR) or prothrombin time (PT) ≤ 1.5 × ULN;
2. Females of childbearing potential are required to use adequate contraception and avoid breastfeeding from screening through 3 months after discontinuation of study treatment. Have a negative pregnancy test prior to initiation of dosing or meet one of the following criteria to demonstrate absence of risk of pregnancy:
3. Postmenopausal is defined as age greater than 50 years and amenorrhea for at least 12 months after stopping all exogenous hormone replacement therapy;
4. Women younger than 50 years of age may also be considered postmenopausal if they have been amenorrheic for 12 months or more after stopping all exogenous hormone therapy and their luteinizing hormone (LH) and follicle stimulating hormone (FSH) levels are within the laboratory's postmenopausal reference range;
5. Undergo irreversible sterilization, including hysterectomy, oophorectomy, or salpingectomy, except tubal ligation.

Males must agree to use an adequate method of contraception or have been surgically sterile during the trial and for 8 weeks after receiving trial drug.

4.3.2 Exclusion criteria

Patients with any of the following will not be enrolled in the study:

1. Patients with active bleeding within 2 months from the primary lesion and/or metastases;
2. Hyperactive/venous thrombosis within 6 months, such as cerebrovascular accident (including temporary ischemic attack), deep vein thrombosis and pulmonary embolism;
3. For patients receiving thrombolytic or anticoagulant therapy such as warfarin, heparin or their analogues, low-dose heparin (daily dose of 6,000 ~ 12,000 U for adults) or low-dose aspirin (daily dose ≤ 100 mg) for preventive purposes is allowed provided INR ≤ 1.5 × ULN;
4. Patients with gastrointestinal diseases with bleeding tendency (such as active gastrointestinal ulcer) or investigators judgment that may cause gastrointestinal bleeding, perforation or obstruction, or patients with established fistula;
5. Receiving radiotherapy or surgery within 30 days, except biopsy and palliative treatment for non-target lesions (note: patients undergoing non-major trauma surgery can receive this protocol as early as 15 days after surgery if they recover quickly after surgery and can use anti-angiogenic drugs as assessed by the investigator);
6. Patients with HER2-positive gastric adenocarcinoma;
7. A history of immunodeficiency, including HIV positive or other acquired or congenital immunodeficiency disorders, or a history of organ transplantation;
8. Patients with brain metastasis and/or leptomeningeal metastasis; for subjects with neurological symptoms, CT/MRI should be performed to rule out brain metastasis;
9. Patients with any severe and/or uncontrolled disease including:

- Patients with hypertension that is not well controlled by single antihypertensive drug therapy (systolic blood pressure ≥ 150 mmHg, diastolic blood pressure ≥ 100 mmHg); or use two or more antihypertensive drugs to control blood pressure;
- Patients with acute myocardial infarction, malignant arrhythmia (including QT interval > 450ms for males and > 470ms for females) and grade II and above congestive heart failure (New York Heart Association (NYHA) classification);
- Active or uncontrolled serious infection (NCI-CTC AE Grade ≥ 2 infection);
- Liver diseases such as cirrhosis, decompensated liver disease, active hepatitis or chronic hepatitis (HBV-DNA > 1000 IU/mL) requiring antiviral therapy;
- Poor glycemic control in diabetic patients (fasting blood glucose > 10mmol/L);
- Urinalysis showed urine protein ≥ + +, and confirmed 24-hour urine protein > 1.0 g;

1. Patients with clinically significant ascites, including any ascites that are detected by physical examination, previously treated or currently treated ascites. Asymptomatic patients with only small amount of ascites on imaging were eligible;
2. patients with moderate pleural effusion, or a large amount of pleural effusion on one side, or caused respiratory dysfunction requiring drainage;
3. Uncontrolled metabolic disorder or other nonmalignant organ or systemic disease or reaction secondary to cancer, leading to a high medical risk and/or uncertainty in survival evaluation;
4. Known active pulmonary tuberculosis;
5. Interstitial lung disease requiring steroid hormone therapy;
6. Patients with significant malnutrition;
7. Known hypersensitivity to the study drug;
8. Patients with a history of psychotropic drug abuse and unable to quit or with mental disorders;
9. Female patients who are pregnant or lactating;
10. Participation in other clinical trials within four weeks;
11. History of other primary malignancies, except for the following: 1) malignancies that were in complete remission for at least 2 years prior to enrollment and did not require other treatment during the study period; 2) non-melanoma skin cancer or lentigo maligna with no sign of recurrence after adequate treatment; 3) carcinoma in situ with no sign of recurrence after adequate treatment;
12. Patients with concomitant diseases that seriously jeopardize the patient's safety or prevent patients from completing the study according to the investigator's judgment.

4.3.3 Withdrawal criteria

1. Use of other anti-tumor therapies (including chemotherapy, targeted therapy or biological agents, etc.) that may affect the evaluation of efficacy during the study;
2. Patients who experienced serious adverse events and longer suitable for continued participation in the study according to the investigators’ judgment, or have unintended pregnancy;
3. Patients who are unwilling to continue the clinical trial and insist on withdrawing;
4. The investigator considered termination of the study necessary.

4.3.4 Removal criteria

1. Wrong dose and method of administration;
2. Patients who have received chemotherapy or drugs beyond the protocol during the trial;
3. Those who do not meet the criteria but were included by mistake;
4. Patients without medication;

Note: Patients who met criteria 1-3 are included in the safety analysis.

4.3.5 Discontinuation criteria

1. Patients with PD;
2. The researchers found severe safety problems based on the decisions of investigators;
3. Drug withdrawal for more than one cycle;
4. Dose reduction for more than 2 times;
5. Surgery is feasible after treatment and patients voluntarily undergo surgery.

Note: In the first 6 cycles of treatment, if one of the chemotherapy drugs has been dose reduced for more than twice or discontinued for more than one cycle, while the other chemotherapy drugs can be used alone (e.g., only platinum is intolerant, while capecitabine, paclitaxel or gemcitabine are still tolerable), the latter and anlotinib can be retained; if the retained chemotherapy drugs cannot be used alone, e.g., platinum, the patients should be withdrawn from the study. After 6 cycles, anlotinib maintenance could be continued if capecitabine was intolerable while anlotinib is tolerable.

5 Study drugs

5.1 Study drugs

Anlotinib Hydrochloride Capsules: manufactured and provided by Chia Tai Tianqing Pharmaceutical Group Co., Ltd.

Strength: 12 mg/capsule, 10 mg/capsule, 8 mg/capsule;

10 mg/capsule and 8 mg/capsule are used for dose reduction.

Oxaliplatin, capecitabine, cisplatin, paclitaxel, docetaxel, and other chemotherapy drugs: purchased by the patient.

According to the requirements of GCP, the study drug will be kept, distributed and recycled by the hospital.

Distribute and recycle the drugs according to the cycles. Complete records are required for both distribution and recycle. The recycled drugs will be submitted to the sponsor after completing the trial. The monitor regularly checks the use and recording of drugs and monitors the recycle at any time.

5.2 Dosing Regimen

5.2.1 Dosing Protocol design

Subjects will be treated with a 3-week treatment cycle of anlotinib in combination with chemotherapy. Tumor efficacy is evaluated at the end of cycle 2, 4 and 6; if necessary, MDT team is required to assess the possibility of surgical resection of liver metastases. If surgical resection is possible, anlotinib and chemotherapeutic drugs should be discontinued (4 weeks before surgery or determined by the investigator). In case of the liver metastases is unresectable after 6 cycles of treatment, patients with CR/PR/SD were switched to maintenance therapy with anlotinib (12 mg, po, qd, d1-d14, Q3W) combined with metronomic capecitabine chemotherapy (500 mg, po, bid, daily) until disease progression or intolerance. They were divided into 3 cohorts according to the type of primary tumor; each cohort received a combination of anlotinib and chemotherapy (different chemotherapy regimens) for the first 6 cycles, and maintenance treatment thereafter of anlotinib in combination with low-dose capecitabine or chemotherapy (preferably capecitabine) at the investigator's discretion.

Table 4: Dosing Regimen

|  |  | Drug | Dose | Dose Frequency | Administration method | Course/Treatment Cycle | Notes |
| --- | --- | --- | --- | --- | --- | --- | --- |
| Initial therapy | Cohort A | Anlotinib | 12 mg / capsule | 1 capsule daily for 2 weeks and 1 week off | Oral | 3-week treatment cycle | Fasting before breakfast |
|  |  | Oxaliplatin | 130 mg/m ^2^ | Administration on d1 of each cycle | Intravenous drip  (> 2h) | 3-week treatment cycle |  |
|  |  | Capecitabine | 850 mg/m ^2^ /time | Twice daily, (once in the morning and once in the evening, total dose 1700 mg/m ^2^ ), for 2 weeks, followed by 1 week off | Oral | 3-week treatment cycle | Oral |
|  | Cohort B | Anlotinib | 12 mg / capsule | 1 capsule daily for 2 weeks followed by 1 week off | Oral | 3-week treatment cycle | Fasting before breakfast |
|  |  | Paclitaxel | 135 mg/m ^2^ | Dosing on d1 of each cycle | Intravenous drip  (> 2h) | 3-week treatment cycle | Hormone pretreatment (dexamethasone, diphenhydramine, cimetidine, etc.) before medication |
|  |  | Docetaxel | 75 mg/m ^2^ | Dosing on d1 of each cycle | Intravenous drip  1h | 3-week treatment cycle | Hormone pretreatment one day before dosing |
|  |  | Cisplatin | 60-75 mg/m ^2^ | D1 for each cycle or d1-d3 | Intravenous drip | 3-week treatment cycle | After paclitaxel |
|  | Cohort C | Anlotinib | 12 mg / capsule | 1 capsule daily for 2 weeks followed by 1 week off | Oral | 3-week treatment cycle | Fasting before breakfast |
|  |  | Standard first-line chemotherapy regimen at the investigator's discretion (3-cycle chemotherapy regimen) | | | | | |
| Maintenance treatment |  | Anlotinib | 12 mg / capsule | 1 capsule daily for 2 weeks | Oral | D1-d14 every 21 days | Fasting before breakfast |
|  |  | Capecitabine | 500 mg/time | Twice daily | Oral | D1-d21 administration in 21-day cycles | Oral |

Note: A. If anlotinib was missed and it was confirmed that the time since the next dose was less than 12 hours, no redose was given. B. Recommended body surface area calculation formula: 1. Body surface area (m ^2^ ) = 0.0061 × Height (cm) + 0.0128 × body weight (kg) - 0.1529 ; 2. Body surface area = (Height + Weight - 60 )/100 Allow the actual quantity to be within ± 5% of the calculated quantity. C. Cohort B: Either paclitaxel or paclitaxel is optional at the investigator's discretion; D. For maintenance treatment, capecitabine chemotherapeutic agents are preferred in cohort 3.

5.2. 2 Method of Administration

- **Anlotinib hydrochloride:** The recommended dose is 12 mg once daily taken orally before breakfast. Continuous medication for 2 weeks, stop for 1 week, and 3 weeks as a treatment cycle. If a dose is missed during treatment, it should not be retaken if it is less than 12 hours after the next dose.
- **Capecitabine:** It should be swallowed whole with water within 30 minutes after a meal (breakfast or dinner). It should not be crushed or cut. The first 6 cycles are 2 weeks of continuous medication followed by 1 week stop, 2 times a day (once in the morning and once in the evening with total daily doseas 1700 mg/m^2^. The capecitabine dose was calculated as shown in Table 5 below) at an interval of approximately 12 hours. After 6 cycles, one tablet (500mg) is taken orally twice a day after meals (at a fixed time each day).

Table 5: Capecitabine Dose Calculation

| 100 % of Dose Level = 850 mg/m^2^ twice daily | | Number of tablets for 500 mg strength | |
| --- | --- | --- | --- |
| Body surface area (m ^2^ ) | Average Dose per Dose (mg) | Morning | Evening |
| ≤ 1.20 | 1000 | 2 | 2 |
| 1.21- 1.50 | 1250 | 2 | 3 |
| 1.51- 1.80 | 1500 | 3 | 3 |
| 1.81- 2.10 | 1750 | 3 | 4 |
| ≥ 2.11 | 2000 | 4 | 4 |

Note: The above tables apply only to the 500 mg strength tablets and the 100% dose level refers to 850 mg/m ^2^, twice daily (total daily dose 1700 mg/m^2^) **.**

- **Oxaliplatin**: The recommended dose is 130 mg/m^2^ once according to body surface area with 250 to 500 mL of 5% glucose solution and infuse intravenously for 2 to 6 hours., Administered every 3 weeks (21 days) in the absence of major toxicities.
- **Cisplatin**: 60-75 mg/m^2^, intravenous drip at 3-week cycles, Administered on d1 or divided into d1-d3 per cycle; adequate hydration is necessary before and after administration;
- **Paclitaxel**: At a dose of 135mg/m^2^, intravenous infusion was given over 2 hours, once every 3 weeks on d1 per cycle. As this drug can cause allergic reactions, dexamethasone 20mg was given 12 hours and 6 hours before administration, diphenhydramine 50mg orally and cimetidine 300mg intravenously 30 to 60 minutes before administration. Blood pressure, heart rate, and respiration should be measured every 15 minutes after the beginning of the infusion, and any allergic reactions should be noted.
- **Docetaxel**: The recommended dose is 75 mg /m^2^, drip for 1 hour, every 3 weeks on d1 of each cycle for intravenous drip only. All patients must take glucocorticoids, such as dexamethasone, one day before receiving docetaxel, 16 mg for at least 3 days to prevent allergic reactions and body retention.
- **Other chemotherapy agents:** The investigators administered the recommended dose according to the guidelines, and the administration method is based on the package insert of the corresponding chemotherapeutic drugs.

5.3 Dose modification and discontinuation

5.3.1 Anlotinib Hydrochloride

5.3.1.1 Dose Modification

During treatment, according to the degree of drug-related toxicity experienced by patients (according to NCI CTC AE 5 .0) and possible efficacy benefit, dose adjustment is determined by the investigator. The dose levels of each dose group are: ① Starting dose: 12 mg, qd; ② Dose reduction: 10 mg, qd; ② Dose reduction: 8 mg, qd. If more than 2 dose levels needed to be reduced, treatment is terminated.

Note: If an adverse event occurs during the study, and the investigator judges that the adverse event is related to anlotinib, the dose of anlotinib should be preferentially adjusted to avoid dose adjustment of both drugs at the same time. (If adverse reactions are hand-foot skin reactions, the dose of capecitabine is preferentially reduced)

5.3.1.2 Criteria for dose delay and dose modification of anlotinib

The following table suggests delayed administration time and/or dose level change regimens for study drug-related toxicities. When a patient develops toxic reactions such as abnormal liver function, bleeding, proteinuria and decreased platelet count, the requirements for subsequent separate forms should prevail.

The cumulative duration of dose delay should not exceed 2 weeks in each dosing cycle; and should not exceed 2 times in each dosing cycle to ensure the intensity of the drug. If the delay time or number of delays exceeded the prescribed number, the patient will be withdrawn from the study treatment, but tumor assessment will still be performed according to the study procedures.

| Grade of adverse reactions | Dosing time | Dose Modification |
| --- | --- | --- |
| Grade 0-2 | On-time dosing/Delayed dosing | No change |
| Grade 3 | Dose delay until recovery to <grade 2 ^#^ | Reduce dose level |
| Grade 4 | Dose delay until recovery to <grade 2 ^#^ | Reduce dose level. the investigator can stop the treatment permanently judging by the treatment. |

# If not restored after 2 weeks of delay, treatment should be permanently terminated.

5.3.1.3 Recommended delayed administration time and/or change in the dosage regimen in case of a liver function abnormality occurs (elevated ALT, AST, or total bilirubin)

| AE Levels | Dose adjustment program | Recommendations |
| --- | --- | --- |
| Level 1 | Maintain the original dose | Follow-up as the original plan |
| Level 2 (normal baseline) | Delay administration and reduce the dose level if the AE level is restored to <2 within 2 weeks | Active liver protection and strict monitoring of liver function once a week |
| Level 2 (abnormal baseline) | Maintain the original dose | Active liver protection and strict monitoring of liver function once a week |
| Level 3 | Delay administration and reduce the dose level if the AE level is restored to <2 within 2 weeks | Active liver protection and strict monitoring of liver function twice a week until the toxicity is restored to <2 or there is an explanation |
| Level 4 | Permanent termination of treatment | Active liver protection and strict monitoring of liver function 1–2 times a week until the toxicity is restored to <2 or there is an explanation |

5.3.1.4 Recommended delayed administration time and/or change in the dosage procedure recommended for proteinuria

| AE Levels | Dose adjustment program | Recommendations |
| --- | --- | --- |
| Level 1: Routine urine examination showed urine protein+ or quantitative detection of 24 h urine protein is <1.0g | Maintain the original dose | Follow-up as the original plan |
| Level 2: Routine urine examination showed urine protein++ but quantitative detection of 24 h urine protein is 1.0g–2.0g (not included) | Maintain the original dose | Active treatment and urine monitoring (once a week); if necessary, consult renal clinicians |
| Level 2: Routine urine examination showed urine protein++ or above, quantitative detection of 24 h urine protein is 2.0–3.5g (not included) | Delay administration; reduce the dose level for follow-up medication if the AE level is restored to <2 within 2 weeks | Active treatment; if necessary, consult renal clinicians, terminate the treatment at the third occurrence |
| Level 3: Quantitative detection of 24 h urine protein is ≥3.5 g | Delay administration. Reduce the dose level for follow-up medication if the AE level is restored to <2 within 2 weeks | Active treatment; if necessary, consult renal clinicians, terminate the treatment at the third occurrence |

5.3.1.5 Recommended delayed delivery time and/or change when thrombocytopenia happened

| AE Levels | Dose adjustment program | Recommendations |
| --- | --- | --- |
| Level 1: Platelet count 100–75 × 10^9^/L | Maintain the original dose | Follow-up as the original plan |
| Level 2: Platelet count 75–50 × 10^9^/L | Delay administration and maintain the original dose if the AE level is restored to <2 within 1 week | Routine blood checks once every 2–3 days. In the follow-up visit, blood routine should be reviewed weekly |
|  | Delay administration and reduce the dose level if the AE level is restored to <2 within 2 weeks | Routine blood checkup once every 2–3 days. In the follow-up visit, blood routine should be reviewed weekly |
| Level 3: Platelet count 50–25 × 10^9^/L | Delay administration and reduce the dose level if the AE level is restored to <2 within 2 weeks | Routine blood checkup once every 2–3 days. In the follow-up visit, blood routine should be reviewed weekly |
| Level 4: Platelet count <25 × 10^9^/L | Permanent termination of treatment | Daily review of blood routine until the recovery to ≤2; active infusion of single platelets and active treatment |

5.3.1.6 Recommended delayed dose timing and/or dose level changes in case of bleeding events

| AE Levels | Dose adjustment program | Recommendations |
| --- | --- | --- |
| Level 1 | Maintain the original dose | Follow-up as the original plan |
| Level 2 | Delay administration and reduce the dose level if the AE level is restored to <2 within 2 weeks | Deal with it actively |
| Level ≥3 | Permanent termination of treatment | Emergency medical intervention |

5.3.2 Dose modification principles for oxaliplatin

The recommended dose is 130 mg/m^2^. Up to two reductions are allowed (first dose reduction: from 130 mg/m ^2^ to 100 mg/m^2^, the second dose reduction: from 100 mg/m ^2^ p to 85 mg/m ^2^ ). Before starting a new cycle of treatment, oxaliplatin should not be restarted until the toxicity has recovered to grade 1 or lower. Dose reductions are based on safety, especially neurological safety.

1. If the patient develops neurological symptoms (sensory disturbances, spasms), the following methods are recommended to adjust the dose of oxaliplatin according to the duration and severity of symptoms:

- If symptoms persist for more than 7 days and are severe, the dose of oxaliplatin should be reduced by one dose level.
- If paresthesia without functional impairment persists into the next cycle, the dose of oxaliplatin should be reduced by one dose level.
- If dysfunctional paresthesia persists until the next cycle, oxaliplatin should be discontinued.
- If these symptoms improve after discontinuation of oxaliplatin, continuation of oxaliplatin therapy may be considered.

2) If hematologic toxicity (confirmed by baseline blood count values, such as neutrophils < 1.5 × 10^9^ /L or platelets < 75 × 10^9^ /L), or myelosuppression prior to the start of treatment (Cycle 1), and treatment in the next or first cycle should be delayed until hematological parameters return to acceptable levels (grade 1 and lower).

3) If mucositis/stomatitis occurs, with or without neutropenia, the next dose should be delayed until mucositis/stomatitis recovers to at least grade 1, and/or neutrophil levels ≥ 1.5 × 10^9^ /L.

4) When severe/life-threatening (grade 4) diarrhea, severe (grade 3-4) neutropenia (neutrophils < 1.0 × 10^9^ /L), febrile neutropenia (fever of unknown origin without clinical and microbiological evidence of infection with absolute neutrophil count < 1.0 x 10 ^9^ /L, fever > 38.3℃ or persistent fever > 38℃ for more than 1 hour) or severe (grade 3- 4) thrombocytopenia (platelets < 50 × 10^9^ /L), oxaliplatin must be discontinued until symptoms improve or resolve, and reused oxaliplatin should be reduced by one dose level.

5.3.3 Dose modification principles for capecitabine

All doses of capecitabine were to be calculated in milligrams per square meter of body-surface area (mg per square meter) on the basis of the patient's height and weight at baseline. The body weight of the patients in the study may have changed, but the surface area was assumed to remain similar to the baseline measurement. The capecitabine dose was not to be changed during the study unless toxicity occurred.

Adverse reactions caused by capecitabine can be managed by symptomatic treatment, drug withdrawal and dose adjustment. Once the dose of a drug has been reduced, it should not be increased thereafter. When adverse effects occur, the capecitabine dose adjustment regimen can be handled according to the table below:

Note: Common toxicity reaction grading criteria developed by the National Cancer Institute of Canada Clinical Trials Group (NCIC CTG) were used except for hand-foot syndrome and hyperbilirubinemia.

| AE levels | During treatment | Dose modification for next cycle  (% starting dose) |
| --- | --- | --- |
| Ggade 1 | Maintain original dose | Maintain original dose |
| Grade 2 | | |
| First occurrence | Withhold until resolved to Grade 0-1 | 100% |
| Second occurrence of the same toxicity | Withhold until resolved to Grade 0-1 | 75% |
| Third occurrence of the same toxicity | Withhold until resolved to Grade 0-1 | 50% |
| Fourth occurrence of the same toxicity | Permanent treatment discontinuation | NA |
| Grade 3 | | |
| First occurrence | Withhold until resolved to Grade 0-1 | 75% |
| Second occurrence of the same toxicity | Withhold until resolved to Grade 0-1 | 50% |
| Third occurrence of the same toxicity | Permanent treatment discontinuation | NA |
| Grade 4 | | |
| First occurrence | Permanently discontinue treatment unless the physician believes that continued treatment is in the best interest of the patient, withhold treatment until resolution to Grade 0-1. | 50% |
| Second occurrence of the same toxicity | Permanent treatment discontinuation | NA |

Doses of capecitabine that were missed because of toxic effects were not replaced or resumed. Instead, the patient continued the planned course of treatment.

In the presence of grade 2 or 3 hand-foot syndrome, capecitabine should be withheld until normalization or a decrease in severity to grade 1 is achieved. Capecitabine should be reintroduced at a reduced dose after grade 3 hand-foot syndrome. Capecitabine should be permanently discontinued in patients who have a serious skin reaction from treatment with capecitabine.

Capecitabine can cause hyperbilirubinemia. If there was a drug-related increase in bilirubin of more than 3.0 × ULN or in liver aminotransferase (ALT, AST) of more than 2.5 × ULN, capecitabine was immediately discontinued. When the bilirubin level decreased to 3.0 × ULN or less or the liver aminotransferase level was 2.5 × ULN or less, capecitabine could be resumed.

No dose adjustment is allowede during maintenance therapy.

5.3.4 Dose modification principles for paclitaxel

Despite pretreatment with paclitaxel, severe allergic reactions have been reported in patients receiving paclitaxel. So should pay attention to whether there are allergies; If only mild symptoms such as flushing, skin reaction, heart rate slightly faster, blood pressure slightly lower may not need to stop the drug, can be slowed down. However, if severe reactions occur, such as hypotension, angioedema, dyspnea, or generalized urticaria, the drug should be discontinued and appropriate management should be given.

During the treatment period, if grade III or above drug-related adverse reactions occur, the dose of the original drug needs to be reduced by 20%, up to two reductions are allowed. Patients need more than two dose adjustments should be withdrawn from the study in the investigator's discretion. Before starting a new treatment cycle, paclitaxel can be re-administered only when the toxicity has recovered to grade 1 or lower .

5.3.5 Dose modification principles for docetaxel

During the treatment period, if grade III or above drug-related adverse reactions occur, the dose of the original drug needs to be reduced by 20%, up to two reductions are allowed. Patients need more than two dose adjustments should be withdrawn from the study in the investigator's discretion. Before starting a new treatment cycle, docetaxel can be re-administered only when the toxicity has recovered to grade 1 or lower.

Patients who have already experienced a SAE should not receive docetaxel.

5.3.6 Dose modification principles for cisplatin

During the treatment period, if grade III or above drug-related adverse reactions occur, the dose of the original drug needs to be reduced by 20%, up to two reductions are allowed. Patients need more than two dose adjustments should be withdrawn from the study in the investigator's discretion. Before starting a new treatment cycle, cisplatin can be re-administered only when the toxicity has recovered to grade 1 or lower.

5.3.7 Dose modification principles for other chemotherapy drugs

The investigator can administer the drug according to the recommended dose in the guidelines, and refer to the package insert of the corresponding chemotherapeutic drug for the administration method. Before starting a new cycle of treatment, chemotherapy drugs can be re-administered only when the toxicity is recovered to grade 1 or lower.

5.4 Concomitant medications and treatments

Patients may receive supportive care, which can be supplemented with various medications and related treatments, including antibiotics, analgesics, hormones, fluids, psychotherapy, palliative surgery, or any other symptomatic interventions deemed necessary to ensure optimal supportive care. Investigational antineoplastic agents or chemotherapy/endocrine/immunotherapy are excluded from the definition of supportive care. Unconventional therapies (e.g., herbal medicine or acupuncture) and vitamin/mineral supplementation are permissible if investigators ascertain that they do not impact study endpoints. Patients may also receive bisphosphonates for bone metastases during treatment. In cases where pain associated with bone metastases is inadequately managed by systemic therapy or local analgesia, palliative small-area radiation (with less than 5% of the bone marrow area treated) is permitted as long as the target lesion does not fall within radiation fields. Throughout treatment, granulocyte colony-stimulating factor (G-CSF) and other hematopoietic growth factors may be administered if clinical indications suggest a need to address acute toxic effects such as febrile neutropenia; long-term use of erythropoietin is also allowed.

- 1. Prohibited Drugs or Concomitant medications allowing with caution
- Use anticoagulant or thromboprophylaxis with caution

Anticoagulant or thrombosis-preventing drugs should be used with caution during treatment to avoid increasing the potential risk of bleeding. It mainly includes but is not limited to the following categories of drugs: salicylic acid derivatives: such as aspirin; heparin anticoagulants: such as low-molecular-weight heparin, enoxaparin, tenoxaparin, adecaparin, etc.; prophylactic anticoagulant drugs after cardiovascular and cerebrovascular events: such as clopidogrel, teagrelor, etc.

- Drugs that interfere with liver p450 enzymes

Inducers (catamizine, rifampicin, and phenobarbital) and inhibitors (ketoconazole, itraconazole, erythromycin, and clarithromycin) of CYP3A, substrates (simvastatin, cyclosporine, and pimozide) of CYP3A4, and other drugs metabolized by CYP3A4 (e.g., benzodiazepines, dihydropyridine calcium antagonists (calcium antagonists may be selected as appropriate for hypertension that cannot be controlled by ACEIs) and HMG-COA reductase inhibitors should be used with caution during treatment. Substrates of CYP2C9 (diclofenac, phenytoin, pyridoxicam, S-warfarin, and tolbutamide) and substrates of CYP2C19 (diazepam, promazine, lansoprazole, and S-mephenytoin) should be used with caution.

- Drugs that prolong the qt interval of the heart

Because drugs have the toxic and side effects of prolonging QT interval in clinical practice, it is required to use drugs that prolong QT interval with caution during the study. It mainly includes but is not limited to the following types of drugs:

Antibacterials (clarithromycin, streptomycin, erythromycin, roxithromycin, metronidazole, moxifloxacin);

Antiarrhythmics (quinidine, sotalol, amiodarone, propylamine, procainamide);

Antipsychotics (rifampin, fluphenazine, droperidol, haloperidol, thioridazine, pimozide, olanzapine, chlorzapine);

Antifungals (fluconazole, ketoconazole);

Antimalarials (mefloquine, chloroquine);

Antidepressants (amitriptyline, promizine, clomipramine, dosulepin, doxepin).

Citrus, star fruit, grapefruit and grapefruit sap can affect cytochrome P450 activity and should be avoided in combination.

- Traditional Chinese medicines and immunological preparations with anticancer effects

CFDA approved modern traditional Chinese medicine preparations and immunomodulators for the treatment of cancer (such as lentin-mushroom polysaccharide, Aidhi, compound matrine, cinobufacin, Kanglaite, ginseng polysaccharide, Xiaoaiping, Shenqi Fuzheng, Brucea brucea oil emulsion, Kangai, thymosin, interferon, interleukin-2, BCG vaccine, transfer factor, levamisole, etc.) were not allowed during the trial period.

- Drugs that cannot be combined with chemotherapeutic drugs
- Capecitabine should not be administered simultaneously with solivudine and its analogues (e.g., brovudine).
- Avoid cisplatin combined with nephrotoxic or ototoxic drugs, such as aminoglycoside antibiotics, amphotericin B, cefothiophene, furanilic acid, ethacrylatum, chloramphenicol, etc. Not in combination with drugs that increase uric acid, such as colchicine, probenecid or sulfopipiazone;
- S-1 is contraindicated in combination with solivudine, structural analogues, flucytosine, and fluorouracil anti-tumor drugs.
- Others: Refer to the instructions for contraindications or drug interactions

6 Observation items

6.1 Before study initiation

(1) Assessments within 28 days before study initiation:

- Signing the informed consent form;
- Collect tumor history, other medical history, previous treatment history and basic data, including patient ID, gender, age, contact number, occupation and ethnicity;
- Collect imaging evidence of tumor recurrence, only for patients with postoperative recurrence;
- Examination of five items of hepatitis B, hepatitis C and HIV related indicators;
- Concomitant medications and adverse events.

(2) Assessments within 7 days before study initiation:

- Vital signs and physical examination, ECOG PS, height and body weight and blood pressure;
- Pregnancy test, only for women of childbearing potential;
- ECG and echocardiography;
- Coagulation function, blood routine, blood biochemistry, urine routine, stool routine, thyroid function and tumor marker;
- Examination of microcirculation changes (leading site);
- Tumor imaging examinations (CT or MRI).

6.2 During the trial

- Blood pressure is measured daily during dosing;
- Vital signs and physical examination, ECOG PS, height and weight: measured at the end of each cycle within the first 6 cycles, and at the end of every 3 cycles thereafter;
- Coagulation, hematology, blood biochemistry and ECG; measured at the end of each cycle within the first 6 cycles, and at the end of every 3 cycles thereafter;
- Urinalysis, stool routine, thyroid function, tumor markers and microcirculation test; measured at the end of every 2 cycles within the first 6 cycles, and at the end of every 3 cycles thereafter;
- Imaging examination; measured at the end of every 2 cycles within the first 6 cycles, and at the end of every 3 cycles thereafter;
- Assessment of resectability of liver metastases; At the end of cycle 2, cycle 4 and cycle 6, if the investigator considers it necessary, the MDT team should assess the resectability of liver metastases;
- Concomitant medications and adverse events.

6.3 End of Treatment and follow-up

6.3.1 Out-of -group visit

Discharge time was defined as the date of last receipt of the study drug. If the decision to discontinue the study drug was made within 2 weeks after the previous treatment visit, an exit visit was not required unless deemed necessary by the investigator. If the decision to discontinue the study drug (for progressive disease or other reasons) was made more than 2 weeks after the last treatment visit, an out-group visit was required.

Symptomatic treatment and follow-up were continued for unrecovered adverse events until NCI CTC AE V5 grade 1 or complete recovery.

The following items should be observed and evaluated during the out-group visit:

- Vital signs, physical examination, ECOG PS score, blood pressure, electrocardiogram, blood routine, blood biochemistry, urine routine, coagulation function test, tumor markers, thyroid function, stool routine, microcirculation change detection;
- Imaging examination and evaluation: when the subject is out of the group for any reason, the imaging examination is required in time (±7 days, if the previous examination time is not more than 21 days from the termination of treatment, the examination is not required again when leaving the group). In addition to radiologically confirmed disease progression, patients who discontinued study treatment for other reasons underwent imaging at protocol-defined frequencies, if possible, until documented disease progression, initiation of a new antitumor therapy, or death.
- Concomitant medications and adverse events were recorded.
- Other tests as determined by the investigator according to the patient's condition.

6.3.2 Safety Follow-up Visit

A safety follow-up visit will occur no later than 30 ing to the patient's conditions not more than 21 days, it isanticancer therapy within 30 days after the last treatment, then this safety follow-up visit should be performed within a 30-day window before initiation of the new anticancer therapy. If a patient is unable to return to the study center before starting a new treatment, the study center may collect any new safety information that appears during the end of treatment visit and the start of the new treatment by calling the follow-up phone.

The following observations and assessments should be completed for safety follow-up:

- Vital signs, physical examination, ECOG PS, blood pressure, electrocardiogram, blood routine, blood biochemistry, urine routine, coagulation function test, thyroid function and urine routine;
- Concomitant medication and adverse event;

6.3.3 Survival Follow-up

Survival follow-up will be performed once every 6 months. Clinical or telephone follow-up will be performed to record survival status and subsequent anti-tumor treatment. The date of death will be collected if the subject dies.

7 Efficacy evaluation

Primary end point: Objective Response Rate (ORR, CR + PR)

Secondaryend endpoints: (1) Progression-free survival (PFS);

(2) Disease control rate (DCR, CR + PR + SD);

(3) duration of response (DoR);

(4) Overall survival (OS);

(5) The conversion rate of liver metastases;

(6) Safety.

7.1 Tumor Evaluation Criteria

- Measurable lesions:

Measurable visceral disease: Lesions that can be accurately measured in at least one dimension with the use of a CT scan with a slice thickness of 5mm or less and that most often have a diameter (required documentation) of 10mm or more or that are at least twice the slice thickness with the use of a CT or MRI scan with a slice thickness of more than 5mm.

Measurable pathological lymph nodes: Lymph nodes with pathologic enlargement, a high suspicion of metastasis, and a short axis measurement of 15mm or more must be considered malignant on CT evaluation. The short axis refers to the longest linear dimension perpendicular to the longest diameter of the lymph node evaluated in the same plane as the acquisition scan. Only measurable lesions could be selected as target lesions.

- Non-measurable lesions include:

1. Small visceral metastatic lesions less than 10 mm in the longest dimension or twice the slice thickness if the slice thickness is greater than 5 mm.
2. Abnormal and suspicious metastatic lymph nodes with short axis ≥ 10 mm and < 15 mm;
3. Lesions that are truly non-measurable (e.g., ascites carcinomatosis). All non-measurable lesions can only be selected as non-target lesions.

- Target lesions

All measurable lesions, up to two per organ, and a total of 5 lesions representing all involved organs/tissues should be considered as target lesions;

Target lesions (organs with longest diameter and lymph nodes with short axis measurement) should be selected based on lesion size. Target lesions should be representative of all involved organs/tissues, and lesions with reproducible repeated measurements should be selected;

When recording tumor measurements, the most common diameter of each non-nodal target lesion should be recorded. For measurable pathological lymph nodes that can be considered as target lesions, It’s short-axis measurement value should be used in combination with the measurement value of non-nodular (i.e., organ lesion) target lesions. Therefore, when a complete response (CR) occurs in an abnormal lymph node that is a target lesion, the sum of its diameters will not decrease to a value of zero.;

1. Target lesions were followed and measured at each subsequent time point.
2. The sum of diameters for all target lesions will be calculated and recorded. The baseline sum will be used as a reference value to further characterize the objective tumor assessment of lesions in the measurable dimension.

Assign a single measurement to all target lesions, regardless of size. If no measurement value can be assigned, "too small to measure" will be provided Options. A value of 0 is assigned only when there is a complete response.

"Not Evaluable" lesions options are only used for those lesions that cannot be read for technical reasons, e.g: 1. CT artifacts. 2. patient position resulting in obstruction or inability to see the lesion. 3. The whole lesion cannot be seen due to the thick CT slice. If a lesion is divided into two lesions, the longest diameters of the fragmented portions should be added together to calculate the target lesion sum. If two lesions are fused, there may be a plane left between them that helps to obtain the largest diameter measurement for each individual lesion. If these lesions truly coalesce and are no longer distinguishable, the vector of the longest diameter in this instance should be used as the "coalescing lesion" of the longest diameter.

- Non-Target Lesions

Non-target lesions include all non-measurable and measurable lesions that are not selected as target lesions. Lymph nodes with short axis < 10 mm are considered non-pathological and should not be recorded;

Any indeterminate lesion without a definite diagnosis (e.g., an unspecified solitary pulmonary nodule without biopsy, an unspecified thyroid mass without needle aspiration biopsy) that cannot be distinguished from a benign lesion can be considered a non-target lesion;

All other diseases (or lesion location) including pathological lymph nodes should be considered non-target lesions and recorded at baseline. Although measurements are not required, their presence, disappearance, or unequivocal progression should be followed during the study;

It is possible to record multiple non-target lesions involving the same organ as one item in the eCRF.

7.2 Primary end point

The efficacy is evaluated by the investigators according to the RECIST 1.1 criteria. For patients with CR or PR in the first evaluation, the tumor lesions should be examined again within a specified time to confirm the efficacy. These patients returne to the hospital regularly until disease progression. Patients who are medically operable and who undergo surgery are withdrawn from the trial after the last preoperative imaging assessment, and the time for imaging assessment is used as the corresponding indicator.

- ORR

The proportion of patients whose tumors shrink by a certain amount and remain for a certain period of time, including CR and PR. Objective tumor response is assessed according to RECIST 1.1. Participants must have measurable tumor at baseline, and the response is assessed according to RECIST 1.1 criteria as complete response (CR), partial response (PR), stable disease (SD), and progressive disease (PD).

7.3 Secondary end points

- PFS

Progression-Free Survival (PFS) is defined as the last date of confirmed progression-free status for a subject who do not experience disease progression during the trial. Data from subjects who discontinue participation in the trial (without subsequent imaging) for reasons unrelated to disease progression, as well as those who received post-trial therapy, were censored based on either the time of discontinuation or the initiation of post-trial therapy. Incidents of other tumors were not classified as events indicative of disease progression and thus were not censored.

In cases where imaging studies and evaluations indicated progression, the date recorded for progression was not when imaging first suggested it but rather when definitive confirmation was obtained through imaging. If disease progression was diagnosed via alternative clinical methods, that diagnosis date served as the official date of disease progression. For patients eligible for surgery who underwent operative procedures, the timing of their last preoperative imaging constituted the final date marking their progression-free survival and served as data censoring.

- DCR

Percentage of confirmed cases including complete response, partial response and stable disease among efficacy evaluable patients.

- DoR

Time from the first tumor assessment of CR or PR to the first assessment of PD or death from any cause.

- OS

Time from the first day of starting anlotinib to death due to any cause. If no subject die or is lost to follow-up during the trial or follow-up period, the date of the last follow-up is used as the data censoring;

- Conversion rate of liver metastases:

Refers to the proportion of patients whose initial liver metastases are assessed as unresectable and whose liver metastases are converted to resectable after translational therapy.

8 Safety evaluation

8.1 Definitions

- Adverse events (AEs)

Adverse events are adverse medical events that occur after a patient is admitted to a clinical trial. This period lasts from when the patient signed informed consent and accepted the test drug treatment to 1 month after the ending of treatment. Any adverse medical events were recorded, irrespective of the causal relationship with the test drug.

The researcher should record in detail any adverse events that occur in the patient. The record of adverse events includes description of adverse events and all related symptoms, time of occurrence, severity, duration, measures taken, final results, and outcome.

- **Adverse Drug Reaction (ADR)**

All toxic and unintentional reactions to a drug associated with any dose should be considered as adverse drug reactions (ADRs). A response to a drug implies that there is at least a reasonable possibility of a causal relationship between the drug and AE, which means that such a relationship cannot be excluded.

- **Serious Adverse Events (SAEs)**

SAE refers to all adverse events that occur at any drug dose: resulting in death, life threatening. Note: "Serious" and "life threatening" were defined as the risk of death when the adverse event occurred. Rather than assuming that a more severe adverse event would have killed the patient. Hospitalizations due to signs and symptoms of disease progression should not be reported as serious adverse events. During the trial or safety reporting period, if the final outcome of cancer was death, the event leading to death had to be reported as a serious adverse event.

SAEs include:

A. Death or life threatening

B. Hospitalization or prolonged hospitalization

C. Permanent disability

D. Carcinogenesis

E. Teratogenicity

Other events that should be addressed as SAEs: drug exposure during pregnancy/lactation. In principle, pregnancy and lactation were inclusion and exclusion criteria. If a pregnancy occurred during the study, the patient was asked to withdraw from the study immediately, to notify the investigator immediately, and to be followed throughout the pregnancy and after the birth. Even if both mother and child are completely normal without any adverse events, consequences should be documented. Even if the pregnancy is not an SAE, it should be reported using the SAE report form.

8.2 Recording and assessing of adverse events

AEs are described in medical terms, and all aes should be documented in the appropriate section of the Case Report Form (CRF), in addition to the completed SAE report form (including initiation or follow-up reports)." All cases participating in the trial should be included in the summary, and the reasons for withdrawal or exclusion should be explained. If there is a death or severe toxicity case, a detailed case report should be made. In the cases of death, the causes of death should be determined and the relationship between the causes of death and the test drugs should be investigated. Nonresolved adverse events were followed, and all adverse events should be followed until resolved or the patient's condition is stable.

The following aspects of each event should be recorded in the CRF

- Onset time (start time), recovery time (end time)
- AEs will be assessed by the investigator according to NCI CTC v5.0 for assessment and grading:

Grade I (mild): uncomfortable feeling, but not affecting normal daily activities;

Grade II (moderate): discomfort enough to reduce or affect the normal daily activities;

Grade III (severe): incapable of working or normal daily activities;

Grade IV: Life-threatening or disabling;

Grade V: Death.

- Criteria for judging the relevance of drugs to adverse events

|  | 1 | 2 | 3 | 4 | 5 |
| --- | --- | --- | --- | --- | --- |
| Definitely Related | + | + | + | + | + |
| Probably Related | + | + | + | + | ? |
| Possibly Related | + | + | ± | ± | ? |
| Unlikely Related | + | -- | ± | ± | ? |
| Unrelated | -- | -- | -- | -- | -- |

Note: + affirmative, - negative, ± difficult to affirm or negative, ? indicating that the situation is unknown

- Measures taken for the study drug (none, discontinuation of treatment, dose reduction, delay of treatment, slowing of the rate of intravenous infusion), other measures (none, concomitant medication, need for or extension of hospitalization, surgery, delay of chemotherapy, discontinuation of chemotherapy, and dose reduction of chemotherapy)
- Consequences are defined as follows: cured with sequelae, cured without sequelae, not cured but without treatment, not cured requiring treatment, and death. Change in toxicity grade/severity is serious: yes or no. If the patient presents with the same AE several times, each time it must be recorded and reevaluated.

Criteria for determining whether an abnormal objective test result should be reported as an adverse event are as follows:

- the test result is related to (and/or) the accompanying symptoms;
- The findings warrant other diagnostic tests or therapeutic measures/surgical interventions (and/or);
- The results of the test lead to a change in the subject's drug dose or discontinuation of the trial, the need for the addition of other concomitant drugs, or other treatment (and/or)
- The findings were considered by the investigator to be reported as an adverse event.

Merely to repeat testing for an abnormality, but not meeting any of the above criteria, does not constitute an adverse event. Any abnormal test result judged to be an error was not required to be reported as an adverse event.

8.3 Reporting System and Procedures for Serious Adverse Events

Any SAEs in the clinical trial must be immediately informed to the Ethics Committee and the sponsor (below). The investigator should complete and submit the report of SAEs within 24 h to the Ethics Committee, upstream authorities, and the sponsor. The contents of the written report include the time of SAEs, severity, duration, measures, and outcome.

| Reporting unit | Contact number |
| --- | --- |
| Ruijin Hospital affiliated to Shanghai Jiao Tong University School of Medicine | 021-34188900 |
| Division of Pharmacovigilance, Chia Tai Tianqing Pharmaceutical Group Co., Ltd. | Email: cttqpv@163.com |
| Shanghai Municipal Food and Drug Administration | Fax: 021-63558718 |
| Jiangsu Food and Drug Administration | Fax: 025 - 83273714 |
| China Food and Drug Administration | Fax: 010-88363228 |

The researchers and the main investigators at each center decide whether the patient with SAE should be unblinded. The emergency letter is prepared by a specialist in the Statistics Department and distributed to the main investigators of each research center for record purposes. If unblinding is necessary, the project manager of sponsor and inspectors from each center should be informed. Once unblinded, the case should be removed from the trial

8.4 Symptomatic treatment of common adverse reactions Advice

For dose modifications or discontinuations of drugs following adverse reactions refer to 5.3.

8.4.1 Palmar-plantar erythrodysesthesia syndrome

For patients with level 1 toxicity, support treatment is not required. Patients with level 2 or more toxicity consider the following symptomatic supportive treatment, including strengthening skin care, keeping the skin clean, avoiding secondary infection, avoiding stress or friction, using moisturizer or lubrication agents, topical use of urea and corticosteroid ingredients of the emulsion or lubricant, and the use of local anti-fungal or antibiotic treatment if necessary (dose adjustment principles for capecitabine refer to 5.3.3).

8.4.2 Hypertension

Hypertension staging and routine treatment recommendations

Hypertension refers to pathological increased blood pressure, with repeated measurement giving values of >140/90 mmHg.

Severity Rating:

Level 1: early stage of high blood pressure: (systolic blood pressure 120–139, diastolic blood pressure 80–89 mmHg) with no indications for the use of antihypertensive drugs only to monitor the blood pressure.

Level 2: first stage of hypertension (systolic blood pressure 140–159 mmHg, diastolic blood pressure 90–99 mmHg), requires medical intervention, repeated or lasting (≥24 h) symptomatic systolic blood pressure increased by >20 mmHg or the past normal range >140/90 mmHg; monotherapy is required to monitor the blood pressure at the same time; thiazide diuretics are mostly used; however, the use of ACEIs, angiotensin receptor blockers (ARB), β-blockers, and calcium channel blockers are also considered.

Level 3: stage 2 hypertension (systolic blood pressure ≥ 160 mmHg, diastolic blood pressure ≥ 100 mmHg); requires medical intervention; requires multiple medications, usually hiazide diuretics with ACEI or β-blockers or calcium channel blockers.

Level 4: life-threatening (such as malignant hypertension, transient or persistent nerve damage, and high blood pressure crisis) conditions require emergency treatment. Presently, there is no uniform classification worldwide; however, based on the recent perspective of clinical treatment, hypertension can be divided into 2 types:

（1）Hypertension emergencies, diastolic blood pressure of >120 mmHg, with acute or progressive target organ damage, such as cerebral infarction, intracranial or subarachnoid hemorrhage, and hypertensive encephalopathy, of which, progressive or acute hypertension based on chronic primary hypertension is the most common (approximately 40%–50%);(2) Hypertension urgencies (hypertension urgencies), diastolic blood pressure of >120 mmHg with or exhibits only minor organ damage.

Sodium nitroprusside or nifedipine are used to rapidly decrease blood pressure. Diazepam and phenobarbital are used to stop convulsions. Furosemide and mannitol are used to hydrate, reduce the sodium and reduce the intracranial pressure;

Once the patient has a high blood pressure crisis, the medication should be terminated and the patient should quit the clinical study.

8.4.3 Management of Diarrhea

The supportive care can be given for the presence of level 1-2 diarrhea, such as the use of loradine in the earliest episode (for example, oral administration of 4 mg and 2 mg orally every 2 hours until diarrhea is relieved).

8.4.4 Management of gastrointestinal bleeding

In the case of gastrointestinal bleeding, including fecal occult blood (++) or hematemesis, symptomatic treatment should be administered. In the case of upper gastrointestinal bleeding, fasting should be performed; acid suppression, gastric mucosa protection, hemostasis (Acidum Tranexamicum and Batroxobin), blood transfusion, and supportive treatment are administered. If necessary, octreotide could be used. In the case of lower gastrointestinal bleeding, hemostasis, blood transfusion, and supportive care are given. If the bleeding cannot be controlled, surgery is essential.

8.4.5 Recommendations for Management of Proteinuria

During the entire treatment period, all patients were closely monitored for proteinuria, particularly those with a history of hypertension, and 24 h urinary protein was measured for 2 consecutive urine proteins ≥++. After the appearance of proteinuria, the principle of dose adjustment is followed based on the relevant information in the dosing regimen.

8.4.6 Management of Hyperlipidemia and Hyperglycemia

The treatment of hyperlipidemia should consider the patient’s pre-treatment state and eating habits. In addition to diet control, high levels of hypercholesterolemia (≥7.75 mmol/L) at ≥grade 2 or hypertriglyceridemia of ≥level 2 (≥2.5-fold of normal upper limit), HMG-CoA reductase inhibitors (atorvastatin), or appropriate lipid lowering drugs are administered.

8.4.7 Gastrointestinal Toxicity

Oxaliplatin, cisplatin, etc. can cause nausea, vomiting, and diarrhea, which are sometimes severe. If dehydration begins, fluids and electrolytes should be replaced immediately. Preventive and/or therapeutic antiemetic/antidiarrheal medications are recommended. At present, 5-HT3 receptor antagonists ondansetron, granisetron and ramosetron are widely used in clinical practice for chemotherapy-induced vomiting.

8.4.8 Renal Toxicity

Prevention and treatment of nephrotoxicity: drinking water or infusion in advance, in order to reduce the deposition of platinum in the kidney and reduce the nephrotoxicity of platinum drugs, drinking water or infusion 1 ~ 2 liters before administration.

Administration of sodium thiosulfate: Sodium thiosulfate, when combined with cisplatin, reduces the degree of activation of cisplatin, and its high concentration in the kidney can reduce the reabsorption of cisplatin in the renal tubules.

Give cytoprotective drugs: some cytoprotective drugs such as amifastine, organic selenium preparations, glutathione, vitamin C, etc., can play a role in protecting renal tubules and reducing the renal toxicity of platinum drugs**.**

8.4.9 Hematotoxicity

Paclitaxel, oxaliplatin and cisplatin have certain hematotoxicity. When used alone, it can cause the following adverse effects: anemia, leukopenia, granulocytopenia, thrombocytopenia, sometimes up to grade 3 or 4.

Prevention and treatment of Hematologic toxicity

Correction of anemia: the destruction and suppression of the hematopoietic system by tumors and the bone marrow damage caused by chemotherapy can lead to anemia. When the patient's peripheral blood hemoglobin is less than 110 g/L in men and less than 100 g/L in women, erythropoietin can be used, and it is recommended to use 150 IU/kg three times a week. After 4 weeks, the red blood cell count increased less than 0.1 g/L, and the dosage could be increased to 300 IU/kg, 3 times a week. After 8 weeks, there was no effect.

Correction of leukopenia: This process can be divided into two stages of prevention and treatment, which can be applied granulocyte colony-stimulating factor (G-CSF) and granulocyte macrophage colony-stimulating factor (GM-CSF) respectively.

Correction of thrombocytopenia: 1L-11 can be used to improve platelet cytokines, the recommended dose is 50 μg/ (kg.d), generally used at least 6-24 h after chemotherapy, there is a risk of toxic water and sodium retention.

8.4.10 Neurotoxicity

Sodium channel blockers carbamazepine and gabapentin can be used to reduce the occurrence of neurotoxicity, and some neurotrophic drugs such as vitamin B1, vitamin B6 and vitamin C can be used to improve the symptoms of poisoning. In addition, interferon can reduce the delay and prevent the neurotoxicity caused by cisplatin. For patients with serious adverse reactions, it is necessary to reduce the dose or stop the drug in time and deal with symptoms.

8.4.11 Anaphylaxis

Severe anaphylaxis can lead to death and can occur within minutes after administration and in any dosing cycle, such as rash, urticaria, erythema, pruritus, rarely bronchospasm, and hypotension. These reactions can usually be managed with standard epinephrine, glucocorticoids, and antihistamines.

8.4.12 Hepatotoxicity

The commonly used clinical indicators to detect liver function include transaminase, bilirubin, alkaline phosphatase, serum albumin and clotting time. When hepatotoxicity occurs, hepatoprotective treatment should be actively carried out and liver function should be closely monitored. If the liver toxicity of antineoplastic drugs is obvious, the combination of anti-inflammatory, detoxification and liver protection drugs can be used as appropriate.

8.4.13 Cardiotoxicity

Electrocardiogram abnormalities, particularly QT prolongation, have been observed with anlotinib in clinical studies. Electrocardiogram and cardiac function should be closely monitored during medication. Dose modifications may refer to 5.3.1.2.

Prophylaxis/treatment with cardioprotective agents, such as angiotensin converting enzyme inhibitors/angiotensin II receptor blockers in combination with beta-blockers, may be given at the discretion of the patient.

9 Data Management and Statistical Analysis

9.1 Case Report Form

The investigator or authorized designee should complete relevant information in the electronic case report form by using the electronic entry system (EDC) within the specified time. All relevant data of each follow-up visit of each subject during the trial should be timely and truly recorded, and confirmed and signed. To guarantee the patient's right to privacy, the patient's name will be coded.

9.2 Database establishment

The data manager designated by the statistician should complete the electronic case report form document and system in advance, and prompt the investigator or authorized person to verify and modify the questionable data in time. The database was reviewed correctly and the data were locked by the principal investigator, data manager, statistician and monitor. Data cannot be entered and modified by irrelevant personnel to ensure data security. Electronic case report data must be backed up. Any data changes can be made only after the consent form is signed by the principal investigator, statistician and data manager.

9.3 Data Lock

The automatic verification system checks the data deviation in the eCRF and generates the corresponding query table, allowing the study site personnel to modify and verify the entered data. The system automatically saves all data modification trajectories. It is then transmitted to the data statistics unit through a secure virtual private network. When the principal investigator, co-organizer, statistical analyst and data management personnel are present at the same time, the analysis dataset shall be determined, the reviewed data shall be locked, and the locked data files shall not be changed in principle.

9.4 Selection of Statistical Analysis Data

- Full Analysis Set (FAS): Enrolled patients who used the drug at least once according to the intention-to-treat (ITT) principle.
- Per Protocol Set (PPS): Patients who have completed the treatment for more than 6 cycles (including 6 cycles), comformed to the trial protocol, have good compliance, have not used prohibited drugs during the trial, and complete the content specified in the CRF.
- Safety Analysis Set (SAS): All patients who used the experimental drug at least once and had safety record after treatment.

9.5 Dropouts

All patients who completed the informed consent form and are screened as eligible for the trial had the right to withdraw from the clinical trial at any time. No matter when and why the subjects withdraw from the study, as long as they do not complete a cycle of clinical trials and cannot be evaluated for safety and efficacy, they are all drop-outs (After enrollment, patients who progressed with clear medical evidence are not considered as drop-outs, and imaging are required. Patients who terminates study treatment because of intolerable toxicity after enrollment are not considered drop-outs). When a patient dropped out, the investigator must fill in the reason for dropout in the CRF, complete all the evaluation items that can be completed, and carefully fill in the visit record in the CRF. Patients who only undergo screening but withdraw from the study without obtaining the drug will not be considered as dropouts.

9.6 Statistical Analysis Plan

9.6.1 Analysis of Subject Characteristics

- Age, height and weight: described by mean, standard deviation, maximum, minimum and median;
- Gender, ECOG PS, tumor history and previous anti-tumor treatment history: the constituent ratio will be calculated;

9.6.2 Efficacy Analysis

- ORR

Calculate the proportion of patients with PR + CR in the FAS and the 95% confidence interval.

- DCR

Calculate the proportion of patients with PR + CR+SD in the FAS and the 95% confidence interval.

- DOR

Median time will be estimated using Kaplan-Meier method and event and its 95% confidence interval will be presented.

- OS

OS (months) = (Date of death from any cause-Date of first day on anlotinib + 1) / 30.4375

Median time will be estimated using Kaplan-Meier method and event and its 95% confidence interval will be presented.

- PFS

PFS (months) = (Date of disease progression/death-Date of first day on anlotinib + 1) / 30.4375

Median time will be estimated using Kaplan-Meier method and event and its 95% confidence interval will be presented.

- Conversion rate of liver metastases

The proportion of the number of resectable patients with liver metastases to the total number of cases was calculated.

- **Safety analysis**

Descriptive statistical analysis should be the main method to describe AEs in this trial. Laboratory test results describe the conditions that are normal before the test but abnormal after treatment and the relationship with the test drug in case of abnormal changes.

9.7 Estimation of Sample Size

This is an exploratory single-arm trial, with ORR as the primary study endpoint; α=0.05, β=0.2, and NCSS&PASS 15.0 software are used for calculation.

Cohort A: Colorectal cancer with liver metastases

According to available data: in the NO16966 study, the ORR was 47% in patients with advanced colorectal cancer treated with CAPEOX/FOLFOX4 in combination with bevacizumab as first-line therapy. Based on the preliminary study results of anlotinib in the treatment of patients with colorectal cancer and current clinical practice requirements, assuming that first-line and maintenance therapy with anlotinib in combination with CAPEOX regimen could improve the ORR from 47% to 70%, a sample size of 36 patients is required, and the drop-out rate is calculated as 20%, a total of 45 patients are required to be enrolled in this cohort.

Cohort B: Esophageal squamous cell carcinoma with liver metastasis

According to available data, the ORR of paclitaxel combined with first-line treatment in patients with advanced esophageal squamous cell carcinoma was 48.6%. Based on the preliminary study results of anlotinib in the treatment of patients with advanced esophageal squamous cell carcinoma and current clinical practice requirements, assuming that first-line and maintenance therapy with anlotinib in combination with paclitaxel and cisplatin could increase the ORR from 48% to 75%, a sample size of 25 patients is required, and the drop-out rate is calculated as 20%, a total of 31 patients are required to be enrolled in this cohort.

Cohort C: Other gastrointestinal tumor with liver metastasis

According to available data, the ORR for the first-line standard chemotherapy regimen in the treatment of other advanced gastrointestinal malignancies (e.g., gastric cancer, biliary tract cancer cancer, pancreatic cancer, etc.) were 19.4% to 47.8%. Based on the preliminary study results of anlotinib in esophageal and colorectal cancer and current clinical practice requirements, assuming that anlotinib in combination with standard chemotherapy regimen for first-line and maintenance therapy could increase the ORR from 25% to 47%, a sample size of 33 patients is required, and a drop-out rate of 20% is calculated, a total of 40 patients are required to be enrolled in this cohort.

10 Preservation and intellectual property rights of data collected in the study

The subject's medical history will be kept at the study site for 5 years. Except for the national drug regulatory authority, the subject's data cannot be provided to others without the consent of the sponsor and the co-sponsor. Ownership of subject's medical history data and data or knowledge generated shall belong to the sponsor and the sponsor.

11 Responsibilities of sponsor and Investigator

11.1 Sponsor

1. Provide the investigator with materials and other support, and explain the protocol and filling of various materials to the investigator before the clinical initiation;
2. Dispatch a clinical research associate for regular monitoring visits;
3. The CRA should make sure that he/she can keep in contact with the investigator by phone, fax and mail at any time.
4. The CRA will supervise the investigator to carry out the clinical study in accordance with the approved protocol, check the distribution and recovery of investigational drugs according to relevant regulations, and ensure the consistency between the trial records in the clinical trial and the data in the original report.

11.2 Investigator

1. Have received training on GCP and this trial protocol, and have time to carry out this trial according to the study protocol.
2. Patients should be informed in detail about the study before enrollment, and consent should be obtained from the patients and informed consent should be signed.
3. The investigator is obliged to take necessary measures to ensure the safety of patients. In case of any adverse reaction, the investigator should deal with it and report to the principal investigator immediately according to relevant regulations. Serious adverse reactions were followed up.
4. Carefully fill in the case report form in a timely manner;
5. Actively cooperate with the CRA in regular visits;
6. Complete retention of laboratory test records, clinical records, and the patient's original medical records;
7. In order to ensure the evaluation and supervision of clinical trials by China Food and Drug Administration and the sponsor, the study site shall uniformly preserve all the study data, including the confirmation of all the patients (can effectively check different records), all the original informed consent forms with valid signatures, detailed original records of drug distribution, etc., and the preservation period is 5 years. The ownership of all the data of this clinical trial belongs to the sponsor and co-sponsor. Except for the national drug regulatory authority, the investigator should not provide it to the third party in any form without the written consent of the sponsor.

12 Ethical Guidelines and Informed Consent Form

This clinical trial must be conducted in accordance with the Declaration of Helsinki (2008 Edition) and the relevant clinical trial study specifications and regulations in China. The study protocol should be developed before the initiation of the clinical trial. The study protocol should be discussed and signed by the investigator and the sponsor, and submitted to the Ethics Committee of the hospital for approval before implementation. During the actual implementation of this clinical trial, if it is necessary to revise this protocol, the revised trial protocol should be submitted to the Ethics Committee for approval before implementation. If important new information involving the investigational drug is found, the informed consent form must be revised in writing and submitted to the ethics committee for approval before obtaining the patient's consent again.

Before the clinical trial, the investigator must provide the subjects with detailed information about the clinical trial, including the nature of the trial, trial objective, possible benefits and risks, and the rights and obligations of the patients. The clinical trial can only be started after the patient has fully understood the informed consent and signed the Informed Consent Form.

13 Quality Control and Quality Assurance

- The clinical research site must be a drug clinical research base with clinical research conditions determined by the China Food and Drug Administration;
- The investigators must be physicians trained in clinical trials and work under the guidance of senior professionals;
- The examination of clinical wards before the test must meet the standardization requirements to ensure that the rescue equipment is complete;
- The professional nursing staff will give medication to the subjects to have a detailed understanding of the medication and ensure the compliance of the subjects;
- Each study site must conduct the study in strict accordance with the study protocol and truthfully fill in the case observation form;
- The monitor should supervise the conduct of the clinical trial in accordance with the standard operating procedures, confirm that all data records and reports are correct and complete, and that all case report forms are correctly filled in and consistent with the original data, so as to ensure that the trial is conducted according to the clinical study protocol;
- In case of any SAE, the monitor shall timely report to all the study sites, and temporarily stop the study when necessary;
- All study sites participating in the trial should be audited by the sponsor and drug regulatory authorities. It is particularly important that the investigators and relevant personnel provide convenience and time for monitoring and auditing.

14 Study site and personnel

14.1 Study Site, Site Number, and Principal Investigator (Sort by Site Number)

| Study site | Site No. | Investigator | Title |
| --- | --- | --- | --- |
| Ruijin Hospital affiliated to Shanghai Jiao Tong University School of Medicine | 01 | Zhang Jun | Chief Physician |
| Tongji Hospital | 02 | Jiang Hong | Chief Physician |
| Affiliated Hospital of Gangnam University | 03 | Qi Yong | Chief Physician |
| Wuxi Branch of Shanghai Ruijin Hospital | 04 | Tang Xinyu | Attending Physician |
| Nanjing First Hospital | 05 | Wei Xiaowei | Associate Chief Physician |
| Shanghai Jiading District Central Hospital | 06 | Yan Jun | Chief Physician |
| Jiangsu Cancer Hospital | 07 | Zhu Liangjun | Chief Physician |
| Jiangsu Province Hospital | 08 | Zhu Lingjun | Chief Physician |

14.2 Leading Site and principle investigator

Study Site: Ruijin Hospital affiliated to Shanghai Jiao Tong University School of Medicine

Address: No. 197, Ruijin Er Road, Huangpu District, Shanghai

Post code: 200020

Principle investigator: Jun Zhang

Tel: 021-64370045

E-mail: junzhang@188.com

14.3 Sponsor and responsible personnel

Chia Tai Tianqing Pharmaceutical Group CO., LTD.

Address: Fuying Road, Jiangning District, Nanjing No. 1099

Post code: 210000

Responsible personnel: Ting Yu, Qian Liu

Tel: 15316302615, 15051839375

E-mail: [TING.YU@CTTQ.COM](mailto:TING.YU@CTTQ.COM), Q IANLIU2CTTQ.COM

15 Discussion, Approval and Modification of Study Protocol

The above clinical study plan and clinical study case report form are jointly determined by the principal investigators participating in the clinical trial of each unit after discussion, and can be implemented after being approved by Chia Tai Tianqing Pharmaceutical Group Co., Ltd. and approved by the Ethics Committee of the leading site. During the clinical trial, any amendment to the trial protocol should be reported to the Ethics Committee for approval or filing.

16 Test Summary

The principal investigator should summarize the results of statistical analysis of the trial in an objective and detailed manner, and actively complete the summary report of the clinical trial so as to meet the requirements of NMPA for unified regulation of clinical review of new drugs. Each participating unit should complete the sub-site summary.

17 REFERENCES

1. Cassidy J, Clarke S, Díaz-Rubio E, et al. XELOX vs FOLFOX-4 as first-line therapy for metastatic colorectal cancer: NO16966 updated results. 2011 Jun 28; 105 (1): 58-64.

2. Zhang X, Shen L, Li J, et al. A phase II trial of paclitaxel and cisplatin in patients with advanced squamous-cell carcinoma of the esophagus. Am J Clin Oncol. 2008 Feb; 31 (1): 29-33.

3. Markus Moehler, Kohei Shitara, Marcelo Garrido, et al. Nivolumab plus chemotherapy versus junction as first-line treatment for advanced gastric cancer/gastroesophageal cancer/esophageal adenocarcinoma: first results of the CheckMate 649 study. ESMO 2020; LBA6.

4. N. Boku, M.H. Ryu, D.-Y. Oh, et al. Nivolumab plus versus chemotherapy alone in patients with previously untreated advanced or recurrent gastric/gastroesophageal junction (G/GEJ) cancer: ATTRACTION-4 (ONO-4538-37) study. ESMO 2020; LBA7.

5. Morizane C, Okusaka T, Mizusawa J, et al. Combination gemcitabine plus S-1 versus gemcitabine plus cisplatin for advanced/recurrent biliary tract cancer: the FUGA-BT (JCOG1113) randomized phase III clinical trial. 2019 Dec 1; 30 (12): 1950-1958.

6. Phelip JM, Edeline J, Blanc JF, et al. Modified FOLFIRINOX versus CisGem first-line chemotherapy for locally advanced non resectable or metastatic biliary tract cancer (AMEBICA) -PRODIGE 38: Study protocol for a randomized controlled multicenter phase II/III study. Dig Liver Dis. 2019 Feb; 51 (2): 318-320.

7. Vogel A, Römmler-Zehrer J, Li JS, et al. Efficacy and safety profile of nab-paclitaxel plus gemcitabine progression in patients with metastatic pancreatic cancer treated to disease: a subanalysis from a phase 3 trial (MPACT). BMC Cancer. 2016 Oct 21; 16 (1): 817.

8. Sung H, Ferlay J, Siegel RL, Laversanne M, et al. Global cancer statistics 2020: GLOBOCAN estimates of incidence and mortality worldwide for 36 cancers in 185 countries. CA Cancer J Clin. 2021 Feb 4.

9. Zheng Rongshou, Sun Kexin, Zhang Xin, et al. Analysis of the prevalence of malignant tumors in China in 2015. Chinese Journal of Oncology, 2019, 41 (1): 19-28.

10. Chinese Guidelines for the Diagnosis and Comprehensive Treatment of Colorectal Liver Metastases (2018 Edition). Chinese Journal of Gastrointestinal Surgery, 2018, 17 (6): 527-539.

11. Ai D, Zhu H, Ren W, et al. Patterns of distant organ metastases in esophageal cancer: a population-based study. J Thorac Dis. 2017 Sep; 9 (9): 3023-3030.

12. Chinese Expert Consensus on Diagnosis and Comprehensive Treatment of Liver Metastasis of Gastric Cancer (2019 Edition). Chinese Journal of Practical Surgery May 2019, Vol. 39, No. 5.

13. (USA) Niederhuber (J.E.) And other original works; Clinical Oncology (Fifth Edition) [M]. Yan Sun. Beijing: People's Military Medical Press, 2016.

14. Sun Yan, Advanced Tutorial in Clinical Oncology [M]. Beijing: People's Military Medical Press, 2014.

15. Li SC, Lee CH, Hung CL, et al. Surgical resection of metachronous hepatic metastases from gastric cancer improves long-term survival: a population-based study. PloS One, 2017, 2 (7): e0182255.

16. Oki E, Tokunaga S, Emi Y, et al. Surgical treatment of liver metastasis of gastric cancer: A retrospective cohort multicenter study (KSCC1302). Gastric Cancer, 2016, 19 (3): 968-976.

17. Tsujimoto H, Ichikura T, Ono S, et al. Outcomes for patients following hepatic resection of metastatic tumors from gastric cancer. Hepatol Int, 2010, 4 (1): 406-413.

18. Early Diagnosis and Early Treatment Group of Chinese Society of Oncology. Expert consensus on early diagnosis and early treatment of pancreatic cancer by Chinese Society of Oncology. Chinese Journal of Oncology, 2020, 42 (9): 706-712.

19. Kamisawa T, Wood LD, Itoi T, et al. Pancreatic cancer Lancet, 2016, 388 (10039): 73-85.

20. Witkowski ER, Smith JK, Tseng JF. Outcomes following resection of pancreatic cancer. J Surg Oncol, 2013, 107 (1): 97-103.

21. Tang W, Ren L, Liu T, et al. Bevacizumab Plus mFOLFOX6 Versus mFOLFOX6 Alone as First-Line Treatment for RAS Mutant Unresectable Colorectal Liver-Limited Metastases: The BECOME Randomized Controlled Trial. J Clin Oncol. 2020, 38:3175-3184.

22. Ye LC, Liu TS, Ren L, et al. Randomized controlled trial of cetuximab plus chemotherapy for patients with KRAS wild-type unresectable colorectal metastases liver-limited. Journal of Clinical Oncology. 2013, 31 (16): 1931-1938.

23. Gruenberger T, Bridgewater J, Chau I, et al. Bevacizumab plus mFOLFOX-6 or FOLFOXIRI in patients with initially unresectable liver metastases from colorectal cancer: the OLIVIA multinational randomised phase II trial. Ann Oncol. 2015 Apr; 26 (4): 702-708.

24. Yasuno M, Uetake H, Ishiguro M, et al. mFOLFOX6 plus bevacizumab to treat liver-only metastases of colorectal cancer that are unsuitable for upfront resection (TRICC0808): a multicenter phase II trial comprising the final analysis for survival. Int J Clin Oncol. 2019 May; 24 (5): 516-525.

25. Ken Kato, et al. Pembrolizumab Plus Versus Chemotherapy as First-Line Therapy in Patients With Advanced Esophageal Cancer: The Phase 3 KEYNOTE-590 Study. ESMO 2020. LBA8.

26. Markus Moehler, et al. Nivolumab (NIVO) Plus Chemotherapy (Chemo) Versus Chemo as First-Line (1L) Treatment for Advanced Gastric Cancer/Gastroesophageal Junction Cancer (GC/GEJC)/Esophageal Adenocarcinoma (EAC): First Results of the CheckMate 649 Study. ESMO 2020; LBA6.

27. N.Boku, et al. Nivolumab plus versus chemotherapy alone in patients with previously untreated advanced or recurrent gastric/gastroesophageal junction (G/GEJ) cancer: ATTRACTION-4 (ONO-4538-37) study. ESMO 2020; LBA7.

28. Valle J, Wasan H, Palmer DH, et al. Cisplatin plus gemcitabine versus gemcitabine for biliary tract cancer. N Engl J Med, 2010, 362 (14): 1273-1281.

29. Goldstein D, El-Maraghi RH, Hammel P, et al. nab-Paclitaxel plus gemcitabine for metastatic pancreatic cancer: long-term survival from a phase III trial. J Natl Cancer Inst, 2015, 107 (2).

3 0. Yihebali Chi, Yongqian Shu, Yi Ba, et al. The efficacy and safety of anlotinib in refractory colorectal cancer: a double-blinded, placebo controlled, randomized phase III ALTER0703 trial. ASCO-GI 2021. Poster Highlight Session, P65.

3 1. J. Yan, L zhang, Y jin, Et al. Efficacy and safety of anlotinib plus XELOX regimen as first-line treatment followed by maintenance monotherapy of anlotinib for patients with Mcrc: A single arm, multi-center, Phase II clinical trial (ALTER-C-001) . ASCO-GI 2021. Poster Session, P70.

32. K. Ding, Y. Liu, J. Chen, et al. Anlotinib combined with CAPEOX in first-line treatment of patients with RAS and BRAF wild-type unresectable mCRC: a single-arm, phase II study (ALTER-C-002 trial). ASCO-GI 2021. Poster Session, P75.

3 3. Yan Song, Juxiang Xiao, Jie He, et al. Anlotinib in chemotherapy-refractory metastatic esophageal squamous cell carcinoma (ESCC): a randomized, double-blind, multicenter phase II trial. Journal of Clinical Oncology 2019 37:4suppl, 95-95.

3 4. Jun-Sheng Wang, Su-Xia Luo, Ning Li, et al. Update results of paclitaxel and cisplatin in combination with anlotinib as first-line regimen for advanced esophageal squamous cell carcinoma (ESCC): a multicenter, single-arm, open-label phase II clinical trial. ASCO-GI 2021. Poster Session, 181.
